# Reversible Mechanical Interlocking via Stimuli‐Triggered Nonhomeomorphic Topology Transformation Enables Highly Efficient Rotaxane Synthesis

**DOI:** 10.1002/anie.202513783

**Published:** 2025-09-12

**Authors:** Chunlin Xiao, Xue Li, Naohiro Okamoto, Yuichiro Kobayashi, Tomohiko Nishiuchi, Yosuke Tani, Hiroyasu Yamaguchi

**Affiliations:** ^1^ Department of Macromolecular Science Graduate School of Science The University of Osaka Toyonaka Osaka 560‐0043 Japan; ^2^ Innovative Catalysis Science Division Institute for Open and Transdisciplinary Research Initiatives (ICS‐OTRI) The University of Osaka Suita Osaka 565‐0871 Japan; ^3^ Forefront Research Center Graduate School of Science The University of Osaka Toyonaka Osaka 560‐0043 Japan; ^4^ Department of Chemistry Graduate School of Science The University of Osaka Toyonaka Osaka 560‐0043 Japan; ^5^ Institute of Transformative Bio‐Molecules (WPI‐ITbM) Nagoya University Furo, Chikusa Nagoya 464‐8601 Japan

**Keywords:** [2+2] Photocycloaddition, Macrocycles, Mechanically interlocked molecules, Rotaxanes, Topology

## Abstract

Rotaxanes, as one of the most fundamental mechanically interlocked molecules (MIMs), have attracted considerable attention in many fields. However, a method that reconciles convenient, efficient, and “high‐value‐added” (such as reversibility, higher‐order structures, and diverse topologies) synthesis of rotaxanes, remains challenging. Here, we report a threading‐and‐shrinking strategy to quantitatively prepare [2]rotaxane and bis[2]rotaxane (higher than 99% conversion and 92% isolated yield for [2]rotaxane; 96% conversion, 80% isolated yield for bis[2]rotaxane). The rotaxanes with a chair‐like and an orthogonal geometry were synthesized in one pot by 365 nm UV light irradiation via shrinkage of macrocycle driven by reversible topology transformation between nonhomeomorphic structures, requiring no extra addition of reagents like stoppers and catalysts. The heat‐triggered reverse topology transformation quantitatively converts the chair‐like rotaxanes into *pseudo*‐rotaxanes to achieve mechanical unlocking. In contrast, rotaxanes with an orthogonal geometry exhibit thermal stability and remain mechanically interlocked upon heating.

## Graphical abstract

We design the macrocycles capable of stimuli‐triggered, reversible transformation between nonhomeomorphic topologies (one‐hole ring versus two‐hole figure‐eight ring). By leveraging the ring size change associated with the topology transformation, we achieve reagent‐free, highly efficient, reversible, and one‐pot synthesis of [2]rotaxane and bis[2]rotaxanes. This strategy simultaneously enables convenient, efficient, and functional rotaxane construction, providing new insight into the synthesis of mechanically interlocked molecules and the design of thermally or photochemically gated units in molecular machines.

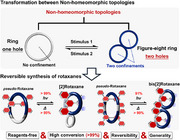

## Introduction

Mechanically interlocked molecules (MIMs) are molecular architectures in which the components are not connected via conventional covalent bonds but are interlocked through their topological arrangement. Rotaxanes, one of the most fundamental MIMs, have demonstrated tremendous potential in fields including molecular machines,^[^ ^1^, ^2^, ^3^, ^4^ ^]^ molecular recognition,^[^ ^5^, ^6^, ^7^ ^]^ and supramolecular materials.^[^ ^8^, ^9^, ^10^, ^11^ ^]^ However, convenient (fewer reagents, reactions, and purification steps), efficient (high conversion and yield), and functional (reversibility, various topologies, or higher‐order structures) synthesis of rotaxanes remains challenging. Since the first statistical synthesis of rotaxane reported by Harrison,^[^ ^12^ ^]^ some strategies such as threading‐and‐stoppering, clipping, and active template synthesis have been established and widely used.^[^ ^13^, ^14^, ^15^, ^16^, ^17^, ^18^ ^]^ However, aside from the axle and wheel molecules that comprise rotaxanes, these methods typically require addition of auxiliary reagents such as large excesses of stoppers, metal ions, or catalysts, thereby complicating both reaction and purification. Other than these methods, without extra addition, Chiu et al. reported the threading‐and‐shrinking and threading‐and‐swelling protocols to obtain rotaxane with isolated yields of 28% and 86%, respectively, where the rotaxanes form via the irreversible transition of homeomorphic topology (rings with different cavities).^[^ ^19^, ^20^ ^]^ Leigh's group first reported the synthesis of [2]rotaxanes driven by stabilization of the axle‐forming transition state with isolated yield of 50%.^[^ ^21^ ^]^ Although these reagent‐free methods rely solely on the axle and wheel molecules, the yields in most cases remain low to moderate. Moreover, expanding these methods to achieve “high‐value‐added” rotaxanes—such as those featuring various topologies, higher‐order structures ([*n*]rotaxanes) or reversible mechanical interlocks—is particularly challenging due to the specific molecular structure imposed on the macrocycles and irreversible change on system's potential energy surface during rotaxanation. Therefore, a refined strategy that reconciles the convenient, efficient, and functional synthesis of rotaxanes is still lacking.

**Scheme 1:**
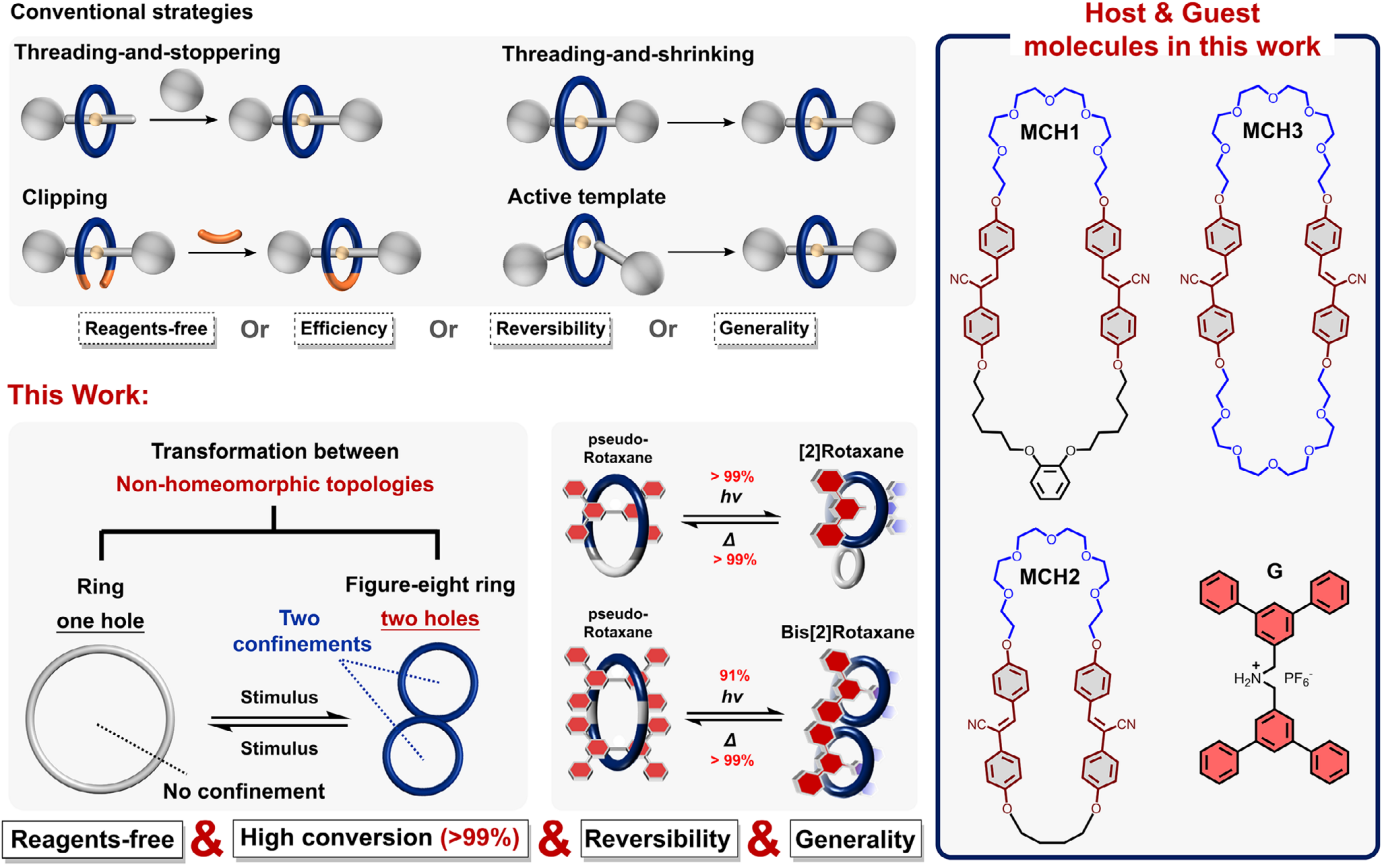
Conventional strategies for rotaxane synthesis (top) and reversible synthesis of rotaxane via nonhomeomorphic topology transformation in this work (bottom).

Here we report a threading‐and‐shrinking strategy for the one‐pot synthesis of [2]‐ and bis[2]rotaxane containing diverse geometries with excellent efficiency (up to 99% conversion and 92% isolated yield for [2]rotaxane; up to 96% conversion and 80% isolated yield for bis[2]rotaxane), in which mechanical interlock is reversible by expansion of macrocycles under heating (Scheme 1). The shrinkage and expansion of macrocycles are achieved by the stimuli‐triggered transformation of nonhomeomorphic topologies, eliminating the need for additional reagents such as stoppers or catalysts during rotaxanation and de‐rotaxanation. First, we design a series of macrocycles incorporating cyano‐stilbene moieties and crown ether fragments in their backbones. The cyano‐stilbene moieties undergo intramolecular [2+2] photocycloaddition upon 365 nm UV irradiation. This photoreaction transforms the macrocycles’ topology from regular ring into the figure‐eight structure containing two smaller rings fused by cyano‐substituted cyclobutane, where two rings are fused in chair‐like or orthogonal conformation. Upon dissolving dibenzylammonium‐containing molecules and the macrocycles in solution, *pseudo*‐rotaxane complexes form wherein the macrocycles act as wheels and the cationic guests as axles. Under 365 nm UV irradiation, the photoreaction of the cyano‐stilbene moieties transforms the complexes from pseudo‐rotaxanes with regular macrocycles into rotaxanes containing chair‐like or orthogonal wheels. Moreover, rotaxanes with chair‐like or orthogonal geometries exhibit totally different stability: under heating, chair‐like rotaxanes convert back to *pseudo‐*rotaxane, while orthogonal rotaxanes maintain their mechanical interlock. Additionally, the rate of this heat‐triggered mechanical unlocking is controllable by adjusting the macrocycle structure. Notably, this approach represents the first demonstration of employing a reversible transformation between nonhomeomorphic topologies (one‐hole‐ring versus two‐hole‐figure‐eight structure) to synthesize rotaxanes and tune their mechanical states. By strategically leveraging the molecular topologies, this work may provide new insight into the synthesis of MIMs with higher‐order mechanical interlocks and the design of thermally or photochemically gated units in molecular machines.

## Results and Discussion

### Reversible Topology Transformation Between Macrocycles and Figure‐Eight Ring Structures

**Figure 1:**
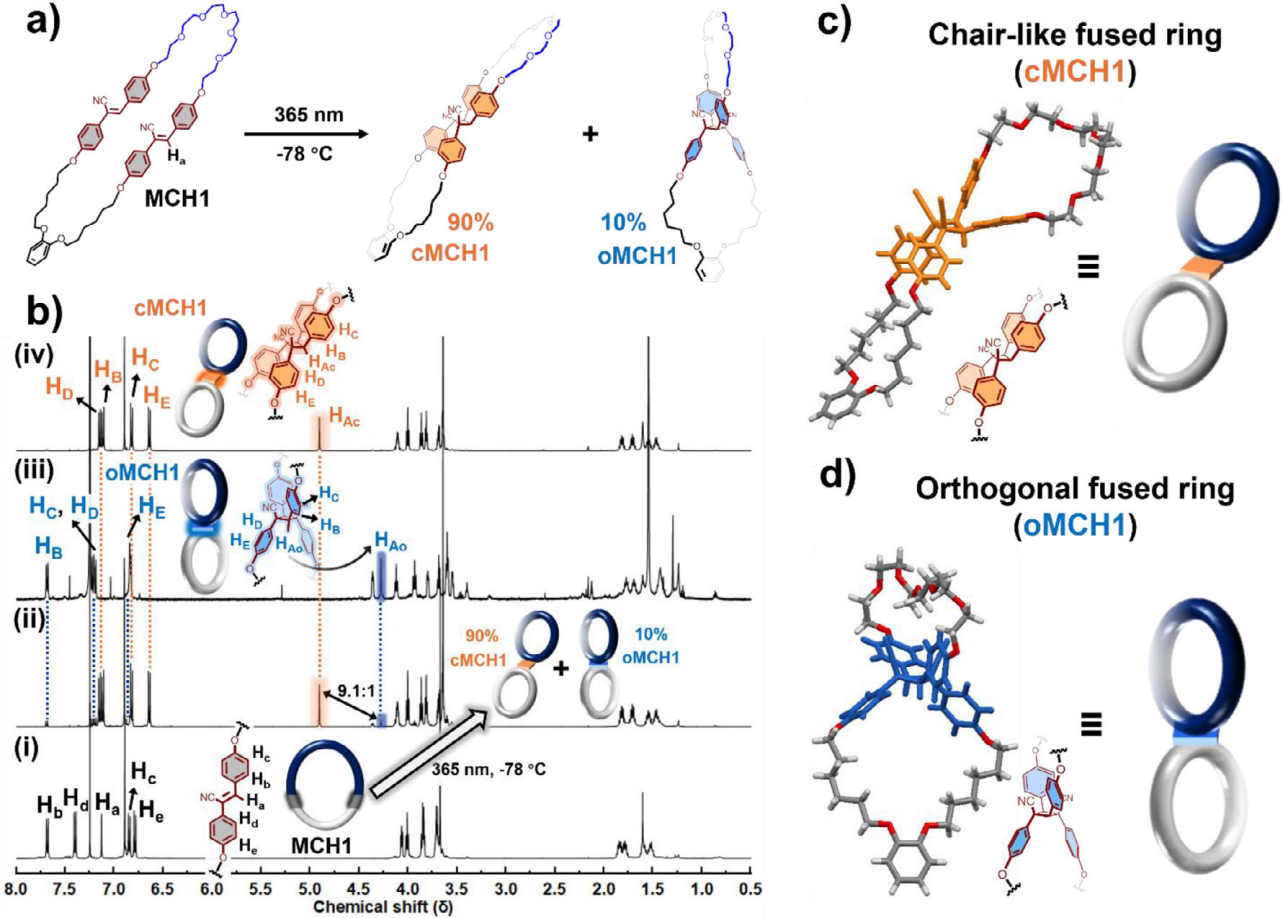
Topology transformation of **MCH1** from a regular macrocyclic structure to a figure‐eight fused ring: a) photo‐induced topology transformation of **MCH1**. b) ^1^H NMR spectra of **MCH1** i), mixture after irradiating **MCH1** ii), isolated **oMCH1** iii), and isolated **cMCH1** iv), CHCl_3_‐d, 500 MHz, 298 K. The structures of c) **cMCH1** (CCDC 2443528) and d) **oMCH1** (CCDC 2443529) determined by single‐crystal X‐ray diffraction. Note: The irradiation temperature was set at −78 °C to maintain consistency in the analysis of subsequent rotaxanation experiments.

Three macrocycles (**MCH*n***, ***n* = 1–3**) were synthesized, each incorporating the same hexaethylene glycol as the crown ether fragment and cyano‐stilbene moiety as the switch for topology transformation, but differing in the structure of the other half of the ring (Scheme 1). The structure of **MCH*n*** is analyzed by ^1^H, HSQC, HMBC, and COSY spectra (**MCH1**: Figures S1–S4; **MCH2**: Figures S15–S18; and **MCH3**: Figures S24–S27). In **MCH1**, the macrocycle transforms to the two smaller rings fused via cyano‐substituted cyclobutene (Figure 1a). The ^1^H NMR spectrum of irradiated **MCH1** shows that all protons (H_a_ to H_e_) of **MCH1** disappeared and the protons of cyclobutane appeared, indicating the completion of [2+2] photocycloaddition (Figure 1b). Two isomers with distinct fused‐linkage (cyclobutane) were obtained: **cMCH1** (**c‐** refers to chair‐like) as the major product and **oMCH1** (**o‐** refers to orthogonal) as the minor product, with a diastereomeric ratio (d.r.) of 9.1, determined by ^1^H NMR integration (Figure S5). Notably, topology transformation from macrocycle to the figure‐eight fused ring structures proceeds quantitatively without any side products (Figure 1b). **cMCH1** and **oMCH1** were both isolated via chromatography. The proton signals in ^1^H NMR spectra of both isomers were assigned according to HSQC, HMBC, and COSY measurements (Figures S6–S9 and S11–S14). Single crystal X‐ray diffraction analysis revealed these two fused‐ring products exhibit completely different molecular geometry: in **cMCH1**, the two small rings adopt a chair‐like geometry, whereas in **oMCH1**, they are arranged in an orthogonal manner (Figures 1c,d). The association between **cMCH1** and a dibenzylammonium cation (**DBA^+^**) was investigated by NMR titration, giving an association constant (*K*_a_) of 325 M^−1^, indicative of moderate complexation (Figure S10).^[^ ^22^ ^]^

For **MCH2**, topology transformation resulted in no orthogonal fused‐ring compound and 100% conversion to the chair‐like fused‐ring (**cMCH2**) as the only product (Figure S19). Similar to the case of **MCH1**, irradiation of **MCH3** also yielded two isomers, chair‐like **(cMCH3)** and orthogonal **(oMCH3)**, with the ratio of **oMCH3** being higher than that observed for **MCH2** (d.r. = 5.0) (Figure S28). Owing to two crown‐ether‐like fragments, **cMCH3** is capable of forming 1 to 2 complexes with **DBA^+^**, where *K*_a1_ and *K*_a2_ are determined as 248 and 256 M^−1^, respectively, by NMR titration experiments. The cooperativity factor (α) is calculated to be 4.13, indicating positive cooperativity (Figure S33).

This isomerism observed in the photoreaction of **MCH1** and **MCH3** originates from the distinct reaction pathways of two cyano‐stilbene moieties. Specifically, [2+2] photocycloaddition between two parallel head‐to‐head arranged cyano‐stilbene moieties leads to the formation of a chair‐like fused‐ring isomer (**cMCH1–3**), whereas an antiparallel head‐to‐tail arrangement results in the formation of an orthogonal fused‐ring isomer (**oMCH1** and **oMCH3**) (Scheme S9). The ^1^H NMR spectra of **MCH1–3** all show single set of proton signals of cyano‐stilbene moiety, indicating the exchange of their arrangement in solution is transient on the NMR time scale. For **MCH3**, the increased flexibility of the hexaethylene glycol fragment may facilitate conformational adjustments that allow **MCH3** to avoid entering the kinetic reaction pathway, thereby favoring the formation of the thermodynamic product (**oMCH3**) in a higher ratio (vide infra). In contrast, irradiation of **MCH2** exclusively yields the chair‐like isomer (**cMCH2**), which is due to the insufficient length of the four‐carbon alkyl linker. This short tether hinders the two cyano‐stilbene moieties from adopting an antiparallel head‐to‐tail arrangement, thereby preventing the formation of the orthogonal isomer via [2+2] photocycloaddition.

Additionally, all chair‐like fused‐ring products (**cMCH1**–**3**) exhibit topological reversibility upon heating, quantitatively converting back to their corresponding macrocycles (**MCH1**–**3**).^[^ ^23^ ^]^ According to the ^1^H NMR spectra recorded at different heating times, the intensity of the cyclobutane proton signals decreased when a chloroform solution of **cMCH1–3** was heated at 80 °C under a nitrogen atmosphere (Figures S38, S40, and S41). Concurrently, signals corresponding to the cyano‐stilbene moieties reappeared, indicating a topology transformation from a chair‐like figure‐eight structure to a regular macrocyclic structure. Among the three compounds, **cMCH1** showed the fastest transformation rate, suggesting that its cyclobutane unit is the most thermally labile. **cMCH3** converted slightly more slowly than **cMCH1**, while **cMCH2** transformed much more slowly than both. In contrast, both orthogonally fused‐ring compounds (**oMCH1** and **oMCH3**) retained their structural integrity, showing no cyclobutane cleavage under heating at 80 °C (Figures S39 and S42). Overall, the thermally stable orthogonal fused‐ring compounds were obtained as minor products, whereas the thermally cleavable chair‐like compounds were the major products, indicating that the intramolecular [2+2] photocycloaddition in macrocycles proceeds under kinetic control.

### Rotaxanation via Topology Transformation from Macrocycles to Figure‐Eight Ring Structures

**Scheme 2:**
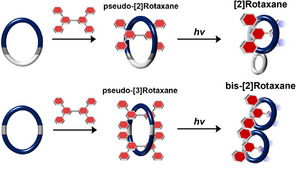
Rotaxanation via the shrinkage of macrocycle driven by topology transformation between nonhomeomorphic structures.

Since [2+2] photocycloaddition is typically chemically orthogonal to supramolecular complexation, resulting in no dissociation between host and guest molecules, the photoactive units in **MCH1–3** can serve as effective switches for ring‐size change, thereby enabling their application in the construction of MIMs.^[^ ^24^, ^25^, ^26^, ^27^, ^28^ ^]^ Subsequently, compound **G**, bearing a dibenzylammonium cation, was employed as the axle molecule to form pseudo‐rotaxane complexes with each macrocycle in solution. Upon simple dissolution of **G** and the macrocycles in acetone, followed by UV irradiation at −78 °C, efficient rotaxanation occurred by the intramolecular [2+2] photocycloaddition between two cyano‐stilbene moieties on macrocycles. The formation of the mechanical bond between the wheel and axle directly arises from a decrease in ring size, in which the shrinkage of macrocycles is driven by a topology transformation between nonhomeomorphic structures (Scheme 2). Additionally, as discussed above, the resulting chair‐like figure‐eight structure is topologically reversible, reverting to macrocyclic structure upon heating. This reverse transformation enables reversible mechanical unlocking and allows subsequent dynamic dethreading (vide infra).

**Figure 2:**
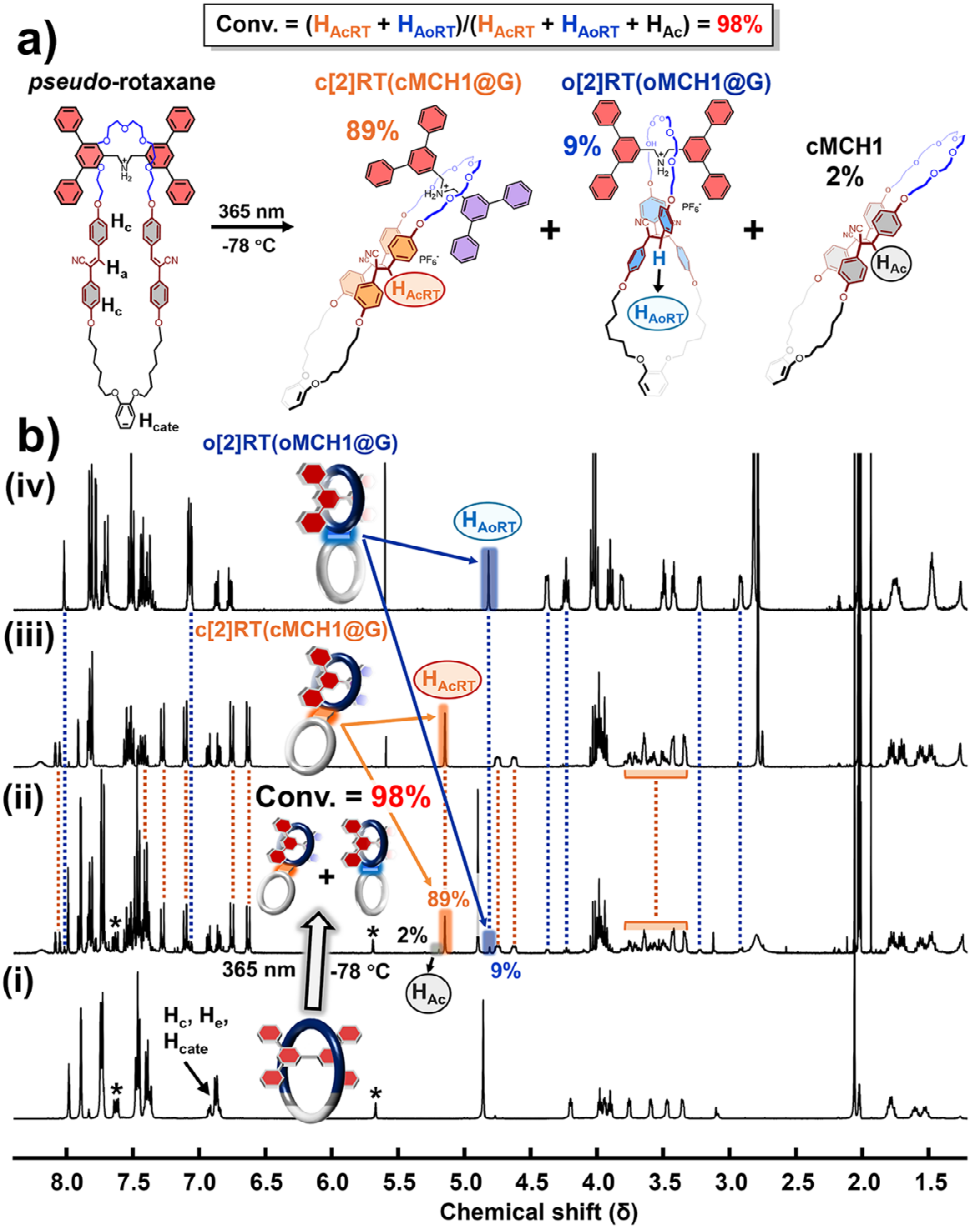
Synthesis of [2]rotaxanes via topology transformation of **MCH1**: a) Photo‐induced rotaxanation and molecular structures. b) ^1^H NMR spectra of mixture of **G** (3 equiv) and **MCH1** (1 equiv) i), crude after irradiating the mixture solution ii), isolated **c[2]RT(cMCH1@G)** iii), isolated **o[2]RT(oMCH1@G)** iv), acetone‐*d*_6_, 500 MHz, 298 K. Note: the asterisks in spectra i) and ii) denote impurities derived from **G** (see figure S46). Note: Acetone is applied as the solvent for all rotaxanation due to the poor solubility of **G** in chlorinated solvents such as dichloromethane and chloroform.

First, we synthesized, isolated, and characterized the rotaxanes using **MCH1** and **G**. A solution of **MCH1** (1 equiv) and **G** (3 equiv) in acetone was degassed with N_2_, followed by irradiation with 365 nm UV light at −78 °C overnight. As shown in the ^1^H NMR spectra (Figure 2b), all proton signals of cyano‐stilbene moieties disappeared, and new signals corresponding to the cyclobutane rings were observed. Similar to the topology transformation of **MCH1** in the absence of an axle molecule, two types of cyclobutane with chair‐like or orthogonal topology were produced in a d.r. of 9.9:1, as determined by ^1^H NMR integration (Figure S45). The observation of two types of cyclobutane indicates the presence of geometrical isomerism in the obtained rotaxanes. Importantly, based on the integration of cyclobutane signals, 98% of the macrocycles were converted into rotaxanes, with only a small portion forming free **cMCH1**. In agreement with the NMR spectra, two rotaxane isomers were successfully isolated by chromatography, both showing identical *m*/*z* values within a few ppm deviation (see synthesis section for all rotaxanes in Supporting Information). One is **c[2]RT(cMCH1@G)**, which exhibits a chair‐like geometry, and the other is **o[2]RT(oMCH1@G)**, exhibiting an orthogonal geometry. The proton signals in ^1^H NMR spectra of two rotaxane isomers are assigned relying on HSQC, HMBC, COSY, and ROESY spectra (**c[2]RT(cMCH1@G)**: Figures S47–S51; **o[2]RT(oMCH1@G)**: Figures S52–S56). The isolated yield of rotaxanes is 92% and the isolated d.r. was determined to be 13:1.

**Figure 3:**
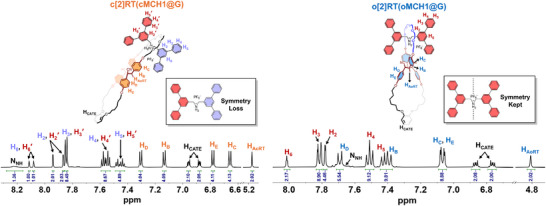
Aromatic region of ^1^H NMR spectra of **c[2]RT(cMCH1@G)** (left) and **o[2]RT(oMCH1@G)** (right), acetone‐*d*_6_, 500 MHz, 298 K.

The geometry difference between **c[2]RT(cMCH1@G)** and **o[2]RT(oMCH1@G)** is clearly revealed by their ^1^H NMR spectra. According to the ^1^H NMR spectra, protons H_1_, H_2_, and H_6_ of axle **G** become spectroscopically nonequivalent in the chair‐like rotaxane, each exhibiting distinct splitting into two sets of signals (H_1_/H_1_', H_2_/H_2_', and H_6_/H_6_') (Figure 3). In contrast, all protons of axle **G** remain chemically equivalent in **o[2]RT(oMCH1@G)**, showing no unexpected splitting (Figure 3). This spectral difference arises from the distinct spatial environments of the axle in the two rotaxanes. In **c[2]RT(cMCH1@G)**, the two cyano groups are located on the same face of the cyclobutane ring (and thus on the same face of the crown ether), which may form weak hydrogen bonds with nearby aromatic protons (H_2_/H_3_/H_4_⋯NC), in a manner analogous to the intermolecular hydrogen bonding observed in the single‐crystal structure of **cMCH1**. In contrast, the orthogonal geometry of **o[2]RT(oMCH1@G)** leads to a symmetric spatial arrangement, with the two cyano groups symmetrically located on opposite sides of the crown ether plane. This spatial symmetry preserves the chemical equivalence of all axle protons, resulting in no observable splitting.

**Figure 4:**
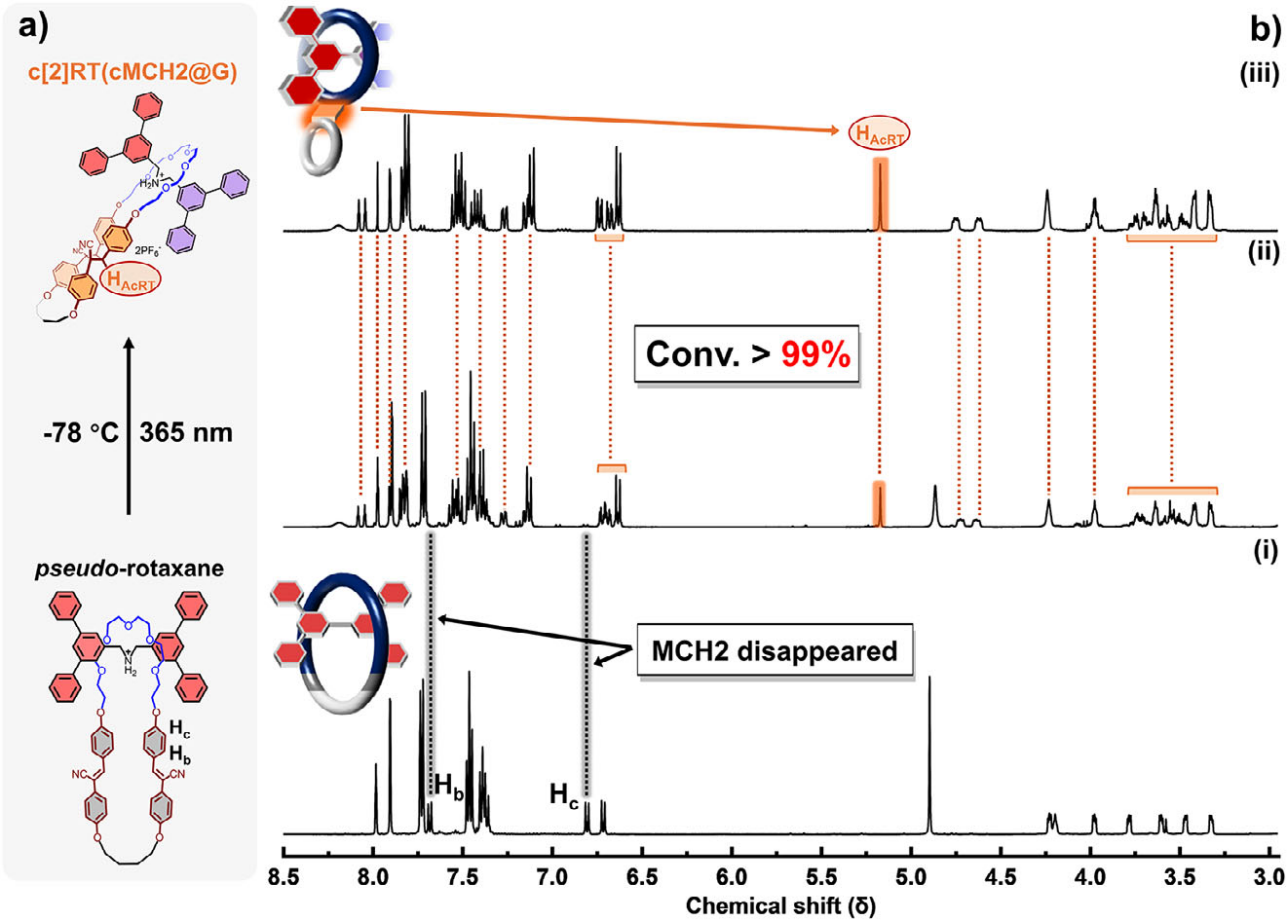
Synthesis of [2]rotaxanes via topology transformation of **MCH2**: a) Photo‐induced rotaxanation and molecular structures. b) ^1^H NMR spectra of mixture of **G** (3 equiv) and **MCH2** (1 equiv) i), crude after irradiating the mixture solution ii), and isolated **c[2]RT(cMCH2@G)** iii), acetone‐*d*_6_, 500 MHz, 298 K.

**Figure 5:**
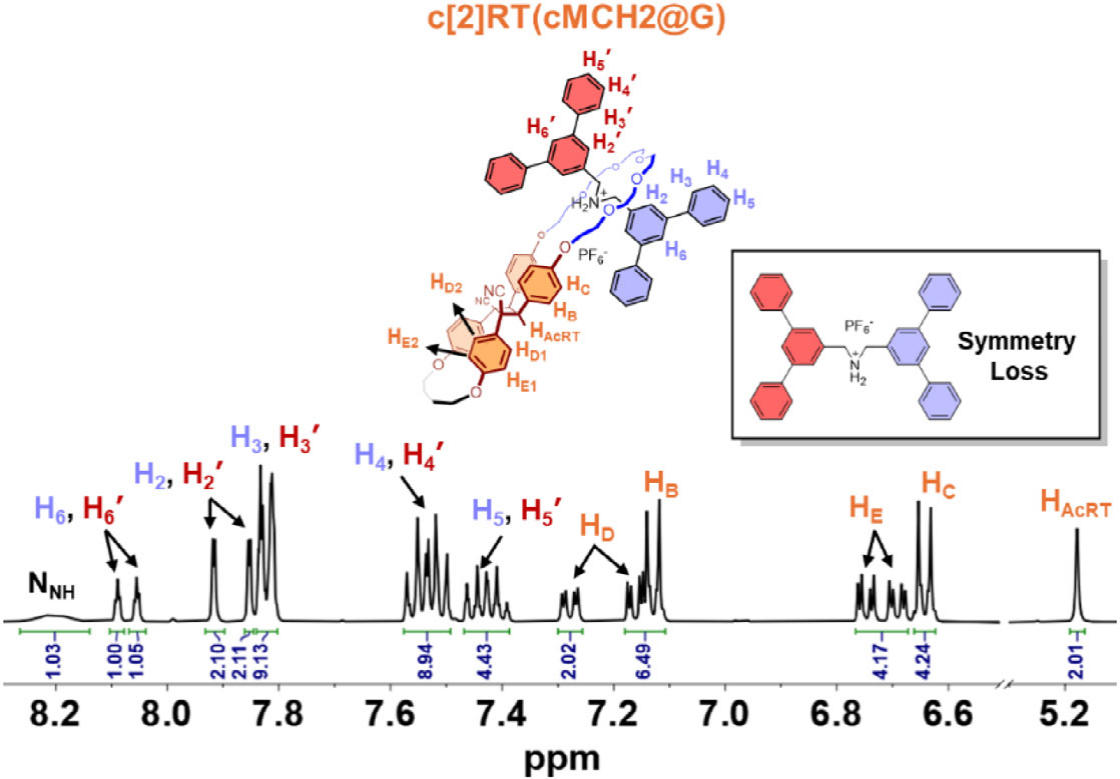
Aromatic region of ^1^H NMR spectrum of **c[2]RT(cMCH2@G)**, acetone‐*d*_6_, 500 MHz, 298 K.

Following the successful synthesis of rotaxanes from **MCH1** and **G**, we applied the same strategy to **MCH2** (Figure 4a). A solution of **MCH2** (1 equiv) and **G** (3 equiv) in acetone was degassed with N_2_, followed by irradiation with 365 nm UV light at −78 °C overnight. As shown in Figure 4b, all proton signals of the cyano‐stilbene moieties in **MCH2** disappeared upon 365 nm UV irradiation, and only signals corresponding to the chair‐like cyclobutane ring were observed, indicating the exclusive formation of a single geometric product. This outcome is consistent with the behavior of **MCH2** in the absence of axle molecules. Remarkably, the rotaxanation proceeded with nearly quantitative efficiency, with the conversion of macrocycles to rotaxanes exceeding 99%. According to the ^1^H NMR spectrum of the product mixture, only excess **G** and the rotaxane product were observed, which was further confirmed by comparison with the spectra of **cMCH2** and isolated **c[2]RT(cMCH2@G)** (Figure S58). The isolated yield of **c[2]RT(cMCH2@G)** is 92%. The ^1^H NMR spectrum shows that the proton signals of axles in **c[2]RT(cMCH2@G)** split in the same way as in **c[2]RT(cMCH1@G)**, indicating a similar chair‐like geometry (Figures 5 and S59–S63). Moreover, in the smallest fused ring formed by a four‐carbon chain, the aromatic protons appear as two distinct sets of signals (H_D1_, H_D2_ and H_E1_, H_E2_), which is similar to the proton splitting in **cMCH2** (Figures S20 and S59). This splitting is attributed to restricted rotation of the phenyl groups, which is likely caused by the limited conformational freedom imposed by the compact fused ring structure.

**Figure 6:**
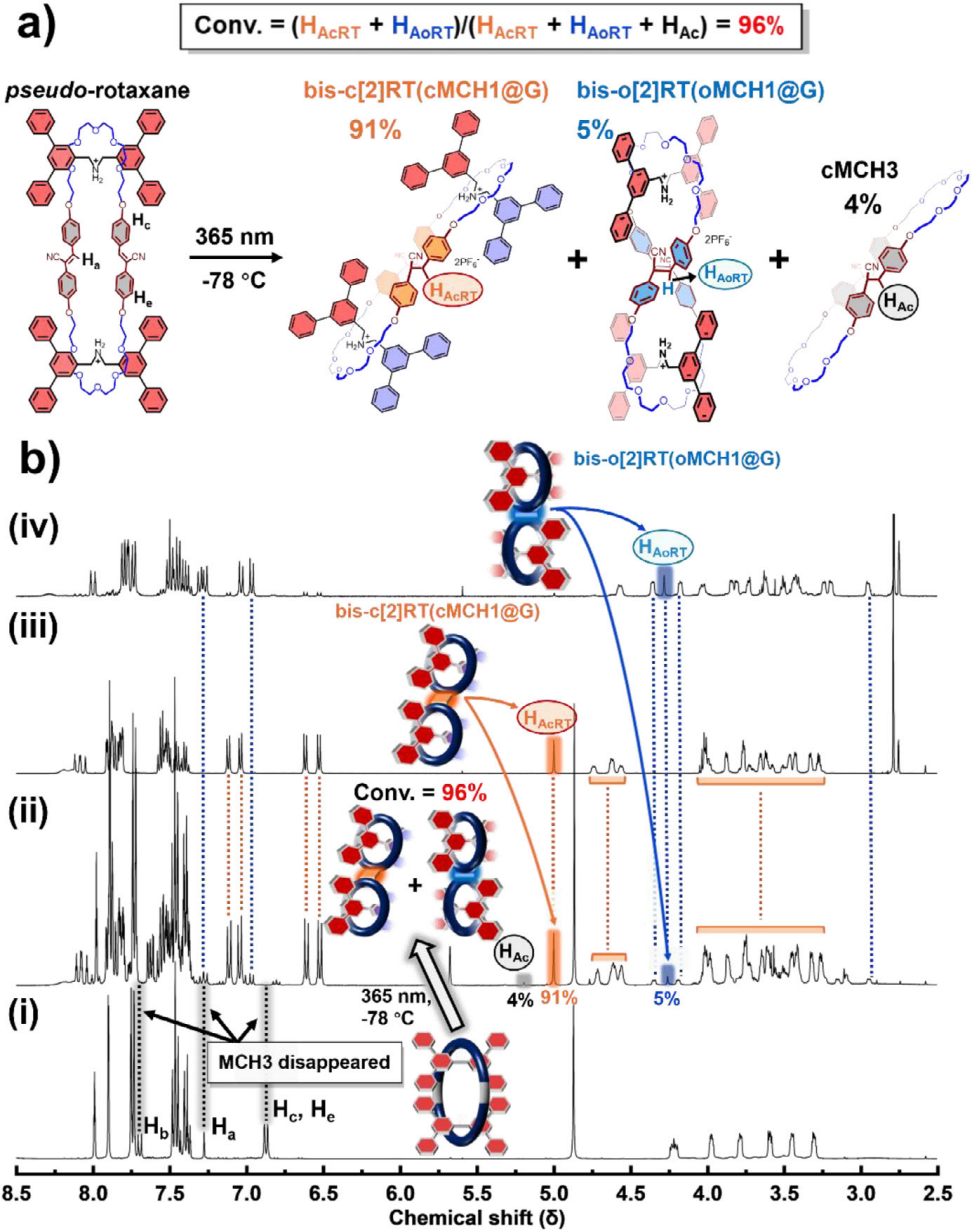
Synthesis of bis[2]rotaxanes via topology transformation of **MCH3**: a) Photo‐induced rotaxanation and molecular structures. b) ^1^H NMR spectra of mixture of **G** (4 equiv) and **MCH3** (1 equiv) i), crude after irradiating the mixture solution ii), isolated **bis‐c[2]RT(cMCH3@G)** iii), and isolated **bis‐o[2]RT(oMCH3@G)** iv), acetone‐*d*_6_, 500 MHz, 298 K.

**Figure 7:**
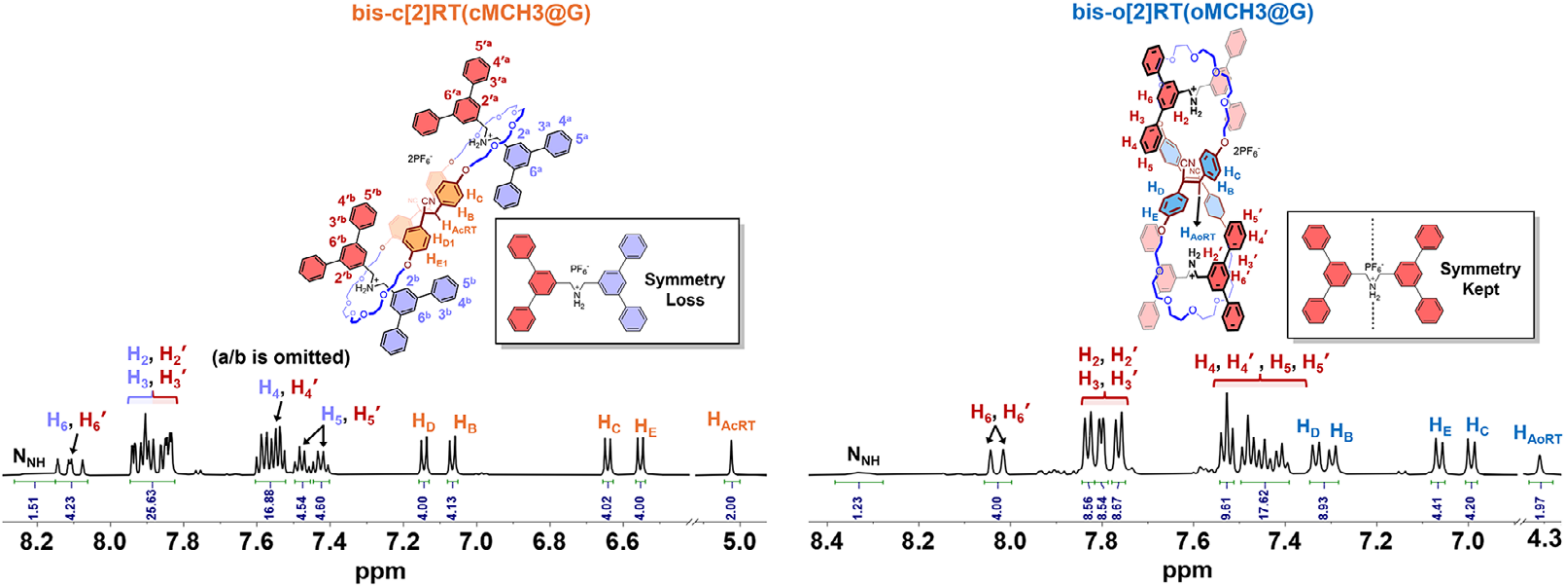
Aromatic region of ^1^H NMR spectra of **bis‐c[2]RT(cMCH3@G)** (left) and **bis‐o[2]RT(oMCH3@G)** (right), acetone‐*d*_6_, 600 MHz, 298 K.

Subsequently, we extended this strategy to the synthesis of MIMs with more complex mechanical interlocks, namely bis[2]rotaxanes, in which one macrocycle is mechanically interlocked with two axles. Using **MCH3** (1 equiv) (a macrocycle bearing two hexaethylene glycol fragments as recognition sites) and **G** (4 equiv), we obtained two types of bis[2]rotaxanes with chair‐like or orthogonal geometries, similar to the topological transformation observed for **MCH3** in the absence of axle molecules (Figure 6a). Rotaxane formation was confirmed by ^1^H NMR spectra, which showed trends consistent with those of former systems (Figures 6b and S65). The conversion of macrocycles to bis[2]rotaxanes reached 96%, with a diastereomeric ratio (d.r.) of 18:1 (chair‐like **bis‐c[2]RT(cMCH3@G)** versus orthogonal **bis‐o[2]RT(oMCH3@G)**). Both bis[2]rotaxanes were successfully isolated by chromatography, with an overall yield of 80% and an isolated d.r. of 30:1.^[^ ^29^ ^]^ According to the ^1^H NMR spectra, **bis‐c[2]RT(cMCH3@G)** exhibits the same splitting pattern of axle protons as the previous two chair‐like rotaxanes, indicating that the two axles adopt a parallel arrangement within the interlocked architecture (Figures 7 and S66–S70). In contrast, the NMR spectrum of **bis‐o[2]RT(oMCH3@G)** reflects the same spatial symmetry observed in **o[2]RT(oMCH1@G)**, suggesting that the two axles are arranged in an orthogonal fashion (Figures 7 and S71–S73). In this orthogonal bis[2]rotaxane, both the two axles and the two macrocyclic components adopt mutually orthogonal orientations, forming a unique geometry in which each structural element, whether axle or ring, is oriented orthogonally to its immediate neighbors. This spatial arrangement gives rise to a highly ordered interlocked architecture.

**Figure 8:**
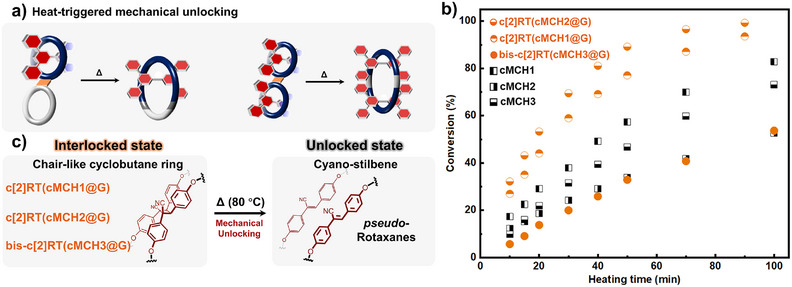
Mechanical unlocking of chair‐like rotaxanes via reverse topology transformation between nonhomeomorphic structures: a) Thermal‐induced transition from rotaxanes to *pseudo*‐rotaxanes. b) Conversion versus heating time curve for the reverse topology transformation of chair‐like figure‐eight structures. c) Schematic illustration of the variation in the system's potential energy surface.

By showing rotaxanation relying on topology transformation of three distinct macrocycles, we demonstrated the convenient, efficient, and structurally versatile synthesis of rotaxanes with diverse geometries and complex mechanical interlocks. Remarkably, the topological reversibility of the chair‐like fused rings endows the resulting rotaxanes with mechanical unlocking capabilities (Figure 8a). In the cases of three chair‐like rotaxanes, according to ^1^H NMR spectra at different heating times, the cyclobutane proton signals (H_AcRT_) progressively decreased upon heating, while the characteristic signals of the *trans*–cyano—stilbene moieties reappeared (Figures S74, S76, and S77). This spectral evolution indicates that the wheel components of the rotaxanes undergo a topology transition back to their original large‐cavity ring forms. As a result, the steric barrier imposed by the figure‐eight structure is eliminated, leading to mechanical unlocking and the conversion of rotaxanes into *pseudo*‐rotaxanes. Similar to the conversion rate of **cMCH1** and **cMCH2** to macrocycle, the conversion of **c[2]RT(cMCH2@G)** to pseudo‐rotaxane state is faster than that of **c[2]RT(cMCH1@G)** (Figures 8b and S78). Upon heating at 80 °C for 70 min, the conversion of the two chair‐like [2]rotaxane reached 97% and 87%, respectively (Figure 8b). For the chair‐like bis‐[2]rotaxane, **bis‐c[2]RT(cMCH3@G)**, 41% conversion of cyclobutane cleavage was reached after heating at 80 °C for 70 min. Complete conversion of all chair‐like rotaxanes was achieved after elongating the heating time or increasing temperature. By comparing the cyclobutane cleavage rates in all chair‐like compounds, we unexpectedly found significant differences in thermal conversion between noninterlocked fused ring structure and their corresponding rotaxanes. For example, the conversion of **c[2]RT(cMCH1@G)** proceeds much faster than that of **cMCH1** and similarly, **c[2]RT(cMCH2@G)** converts more readily than **cMCH2**. Additionally, the stability of **c[2]RT(cMCH1@G)** in DMSO at a temperature slightly above room temperature (40 °C) was confirmed, with no cyclobutane cleavage observed after 1 day (Figure S75). These results suggest that the mechanical interlock in [2]rotaxanes destabilizes the covalent cyclobutane linkage. In contrast, **bis‐c[2]RT(cMCH3@G)** shows a slower conversion than **cMCH3**, indicating that a more complex mechanical interlock can, counterintuitively, stabilize the covalent linkage. We speculate that in [2]rotaxanes, the asymmetric mechanical constraint may induce an uneven distribution of stress in molecular architecture upon heating, thereby facilitating the activation of covalent linkage. In contrast, the more symmetric interlocked structure of the bis[2]rotaxane may help distribute internal stress more evenly, thus stabilizing the architecture.^[^ ^30^, ^31^ ^]^ In contrast, the orthogonal bis[2]rotaxanes show thermal stability, which is similar to other orthogonal compounds (Figures S79 and S80). Based on the results in light‐triggered formation and heat‐triggered de‐formation of mechanical bonds, we expect this clean, efficient, and fully reversible transformation, triggered solely by stimuli and requiring no additional reagents, could serve as a conceptually useful framework for constructing thermally or photochemically gated units in molecular machines.

## Conclusion

In summary, we have developed a threading‐and‐shrinking strategy to achieve reversible mechanical interlocking and unlocking in rotaxanes through a reversible and stimuli‐triggered topology transformation between nonhomeomorphic structures. Upon UV irradiation, the macrocycle in a *pseudo*‐rotaxane undergoes a topological transition from a regular ring to a figure‐eight fused ring with a chair‐like conformation. The resulting smaller cavities prevent the ring from slipping over the axle termini, thereby locking the axle and forming a rotaxane. This one‐pot rotaxanation proceeds without the addition of external reagents such as catalysts or stoppers, achieving up to ∼100% conversion and 92% isolated yield. Conversely, the heat‐triggered reverse topology transformation enlarges the macrocycle cavity, thus triggering mechanical unlocking and converting the rotaxane back to a *pseudo*‐rotaxane. This topology‐transformation‐based mechanism also enables the synthesis of rotaxanes with diverse geometries and complex mechanical interlocks. Orthogonal rotaxanes, in which the fused rings adopt a mutually orthogonal arrangement, were also synthesized and isolated with tunable diastereomeric ratios under different macrocyclic designs. In addition, bis[2]rotaxane consisting of two macrocycles and two axles, featuring both chair‐like and orthogonal geometries, was also efficiently synthesized with a total conversion of 96% and isolated yield of 80%. This topology‐transformation‐based strategy for modulating mechanical interlocks in rotaxanes may provide a new insight for constructing complex mechanical interlocks in supramolecular architecture and thermally or photochemically gated units in molecular machines.

## Conflict of Interests

The authors declare no conflict of interest.

## Supporting information



Supporting Information

## Data Availability

The data that support the findings of this study are available in the Supporting Information of this article.
