# Supplementary material for: Reversible Mechanical Interlocking via Stimuli‐Triggered Nonhomeomorphic Topology Transformation Enables Highly Efficient Rotaxane Synthesis
Source: Angew Chem Int Ed Engl. 2025 Sep 12;64(44):e202513783. doi: 10.1002/anie.202513783 (PMC12559457; doi:10.1002/anie.202513783)
Supplement: Supplementary file 1 — Supporting Information [file ANIE-64-e202513783-s001.docx]

**Reversible mechanical interlocking via stimuli-triggered**

**non-homeomorphic topology transformation**

**enables highly efficient rotaxane synthesis**

Chunlin Xiao^1^, Xue Li^1^, Naohiro Okamoto^1^, Yuichiro Kobayashi^1,2,3*^, Tomohiko Nishiuchi^4^, Yosuke Tani^5^, Hiroyasu Yamaguchi^1,2,3*^.

^1^ Department of Macromolecular Science, Graduate School of Science, The University of Osaka, Toyonaka, Osaka 560-0043, Japan

^2^ Innovative Catalysis Science Division, Institute for Open and Transdisciplinary Research Initiatives (ICS-OTRI), The University of Osaka, Suita, Osaka 565-0871, Japan

^3^ Forefront Research Center, Graduate School of Science, The University of Osaka, Toyonaka, Osaka 560-0043, Japan

^4^ Department of Chemistry, Graduate School of Science, The University of Osaka, Toyonaka, Osaka 560-0043, Japan

^5^ Institute of Transformative Bio-Molecules (WPI-ITbM), Nagoya University, Furo, Chikusa, Nagoya 464-8601, Japan

**Table of contents**

[**1. Synthesis of compounds** 3](#_Toc206003339)

[**2. Topology transformation of MCH1, MCH2, and MCH3 from macrocycles to chair-like and orthogonal figure-eight structures** 52](#_Toc206003340)

[**2.1 MCH1** 52](#_Toc206003341)

[**2.2 MCH2** 61](#_Toc206003342)

[**2.3 MCH3** 65](#_Toc206003343)

[**3. Reverse topology transformation of cMCH1, cMCH2, and cMCH3 from figure-eight structures to macrocycles** 74](#_Toc206003344)

[**3.1 cMCH1** 75](#_Toc206003345)

[**3.2 cMCH2** 77](#_Toc206003346)

[**3.3 cMCH3** 78](#_Toc206003347)

[**3.4 Comparison of topology transformation rate among cMCH1-3** 79](#_Toc206003348)

[**4. Synthesis of chair-like and orthogonal rotaxanes via topology transformation** 80](#_Toc206003349)

[**4.1 Synthesis of c[2]RT(cMCH1@G) and o[2]RT(oMCH1@G) by using MCH1 and G** 80](#_Toc206003350)

[**4.2 Synthesis of c[2]RT(cMCH2@G) by using MCH2 and G** 88](#_Toc206003351)

[**4.3 Synthesis of bis-c[2]RT(cMCH3@G) and bis-oRT(oMCH3@G) by using MCH3 and G** 92](#_Toc206003352)

[**5. Transition from chair-like rotaxanes to *pseudo*-rotaxanes via reverse topology transformation** 98](#_Toc206003353)

[**5.1 Transition from c[2]RT(cMCH1@G) to *pseudo*-rotaxane** 99](#_Toc206003354)

[**5.2 Transition from c[2]RT(cMCH2@G) to *pseudo*-rotaxane** 101](#_Toc206003355)

[**5.3 Transition from bis-c[2]RT(cMCH3@G) to *pseudo*-rotaxane** 102](#_Toc206003356)

[**5.4 Comparison of topology transformation rate of all chair-like compounds** 103](#_Toc206003357)

[**5.5 Thermal stability of orthogonal rotaxanes** 103](#_Toc206003358)

[**6. References** 104](#_Toc206003359)

**Materials and Methods**

Materials

All solvents for synthesis and reactions were purchased from TCI. All other chemicals were purchased from TCI and Nacalai Tesque and used without further purification.

Instruments and Methods

The ^1^H, ^13^C NMR spectra were obtained using a JEOL JNM-ECS 400 and 500 MHz NMR spectrometer, and Agilent VNS 600 MHz NMR spectrometer. All ROESY spectra were obtained using an Agilent VNS 600 MHz NMR spectrometer. All NMR spectra were processed by MestReNova software. ESI-MS and MALDI-TOF-MS spectra were recorded on Bruker micrOTOF-QII and JEOL JMS-S3000 spectrometers, respectively. 2,5-Dihydroxybenzoic acid was used as a matrix in MALDI-TOF measurements. The UV irradiation was performed by using PER-365, Techno Sigma Co., Ltd.

**1. Synthesis of compounds**

**
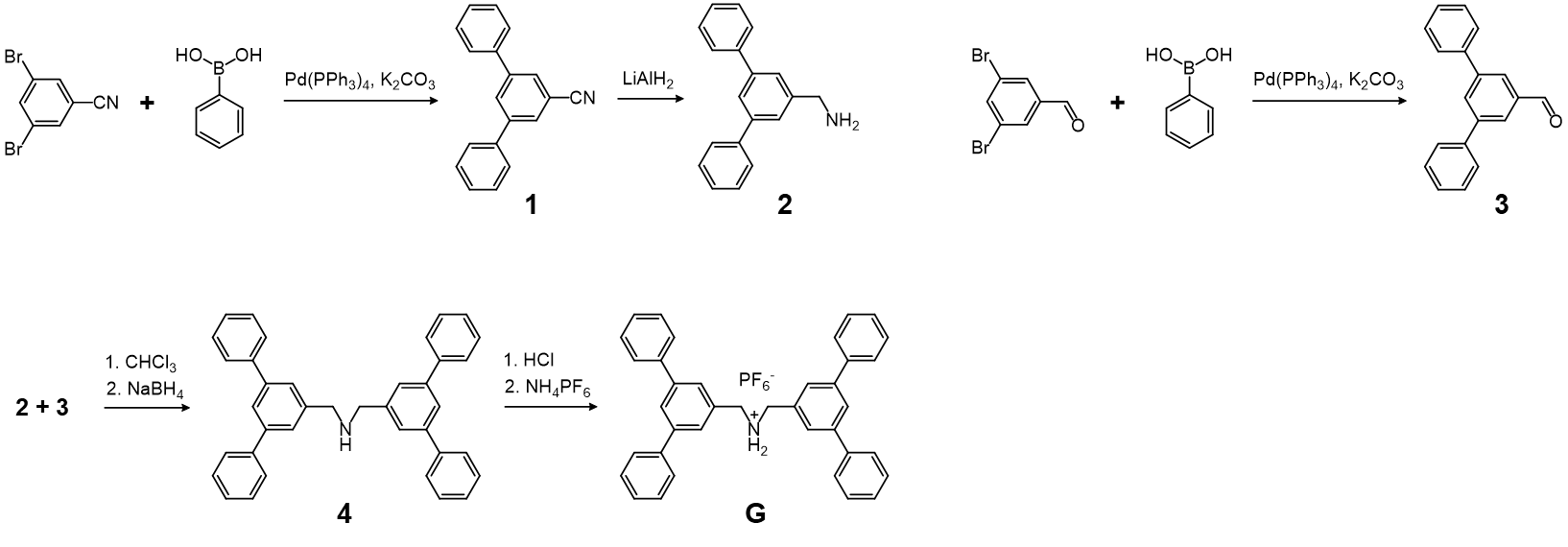
**

**Scheme S1.** Synthetic route of axle molecule **G**

**
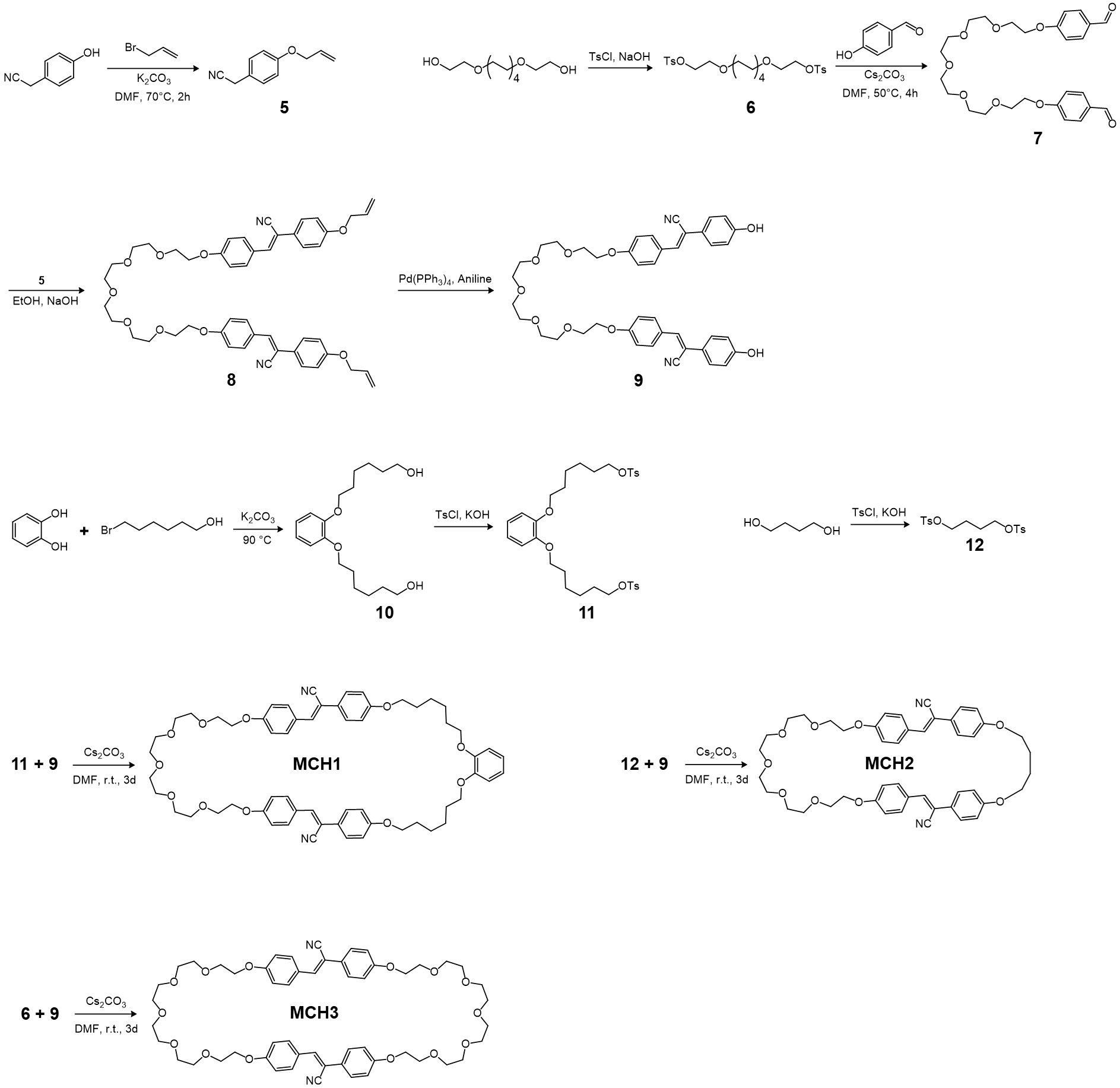
**

**Scheme S2.** Synthetic route of **MCH1**, **MCH1** and **MCH1**

**
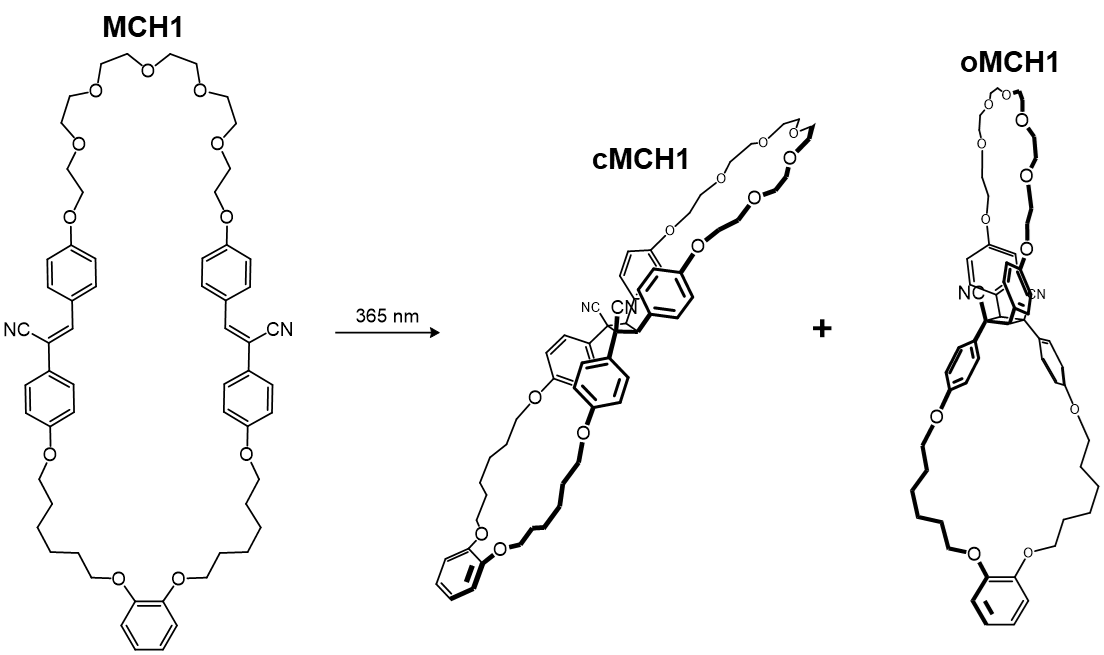
**

**Scheme S3.** Photoreaction of **MCH1** to produce **cMCH1** and **oMCH1**

**
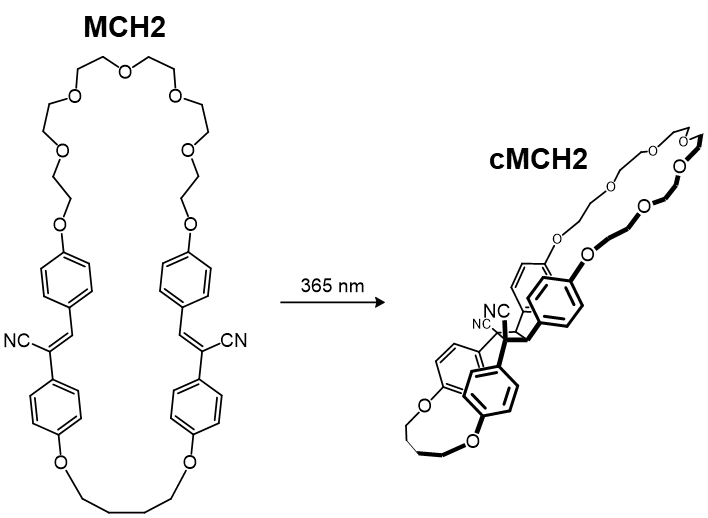
**

**Scheme S4.** Photoreaction of **MCH2** to produce **cMCH2**

**
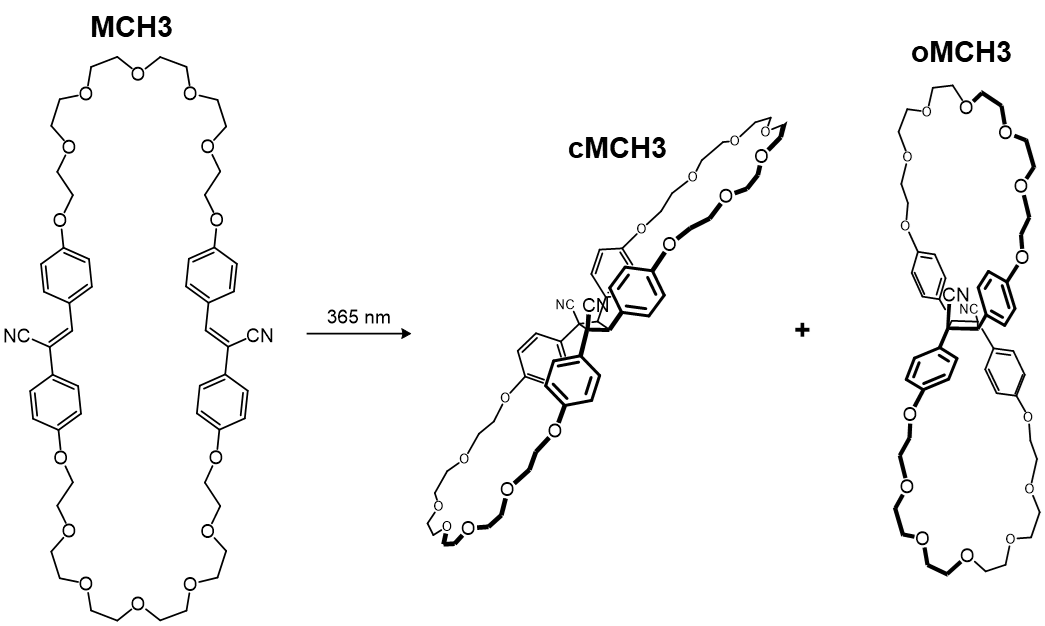
**

**Scheme S5.** Photoreaction of **MCH3** to produce **cMCH3** and **oMCH3**

**
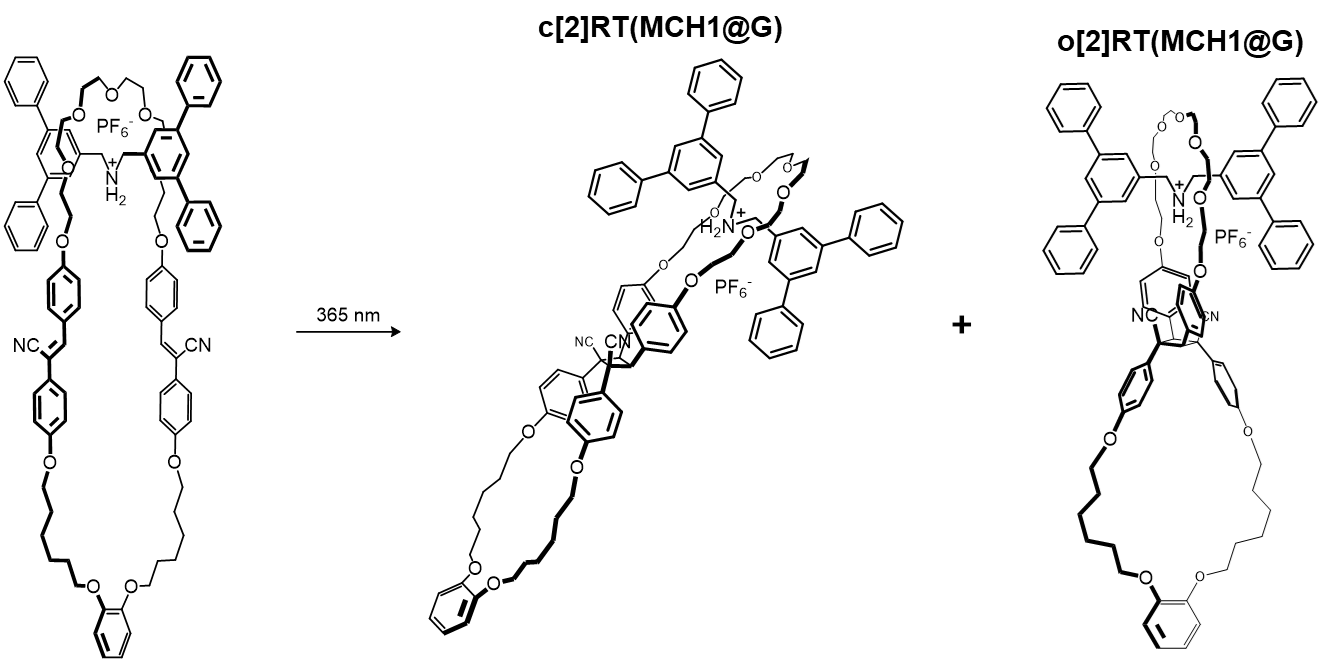
**

**Scheme S6.** Synthesis of **c[2]RT(cMCH1@G)** and **o[2]RT(oMCH1@G)**

**
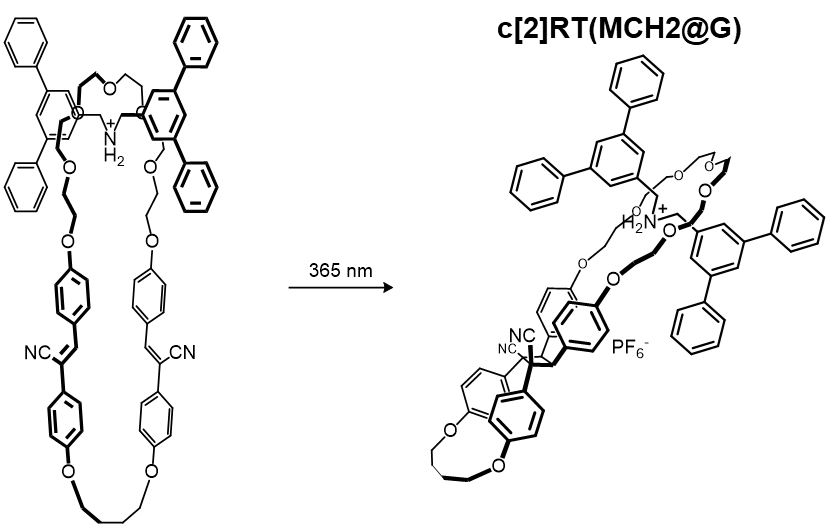
**

**Scheme S7.** Synthesis of **c[2]RT(cMCH2@G)**

**
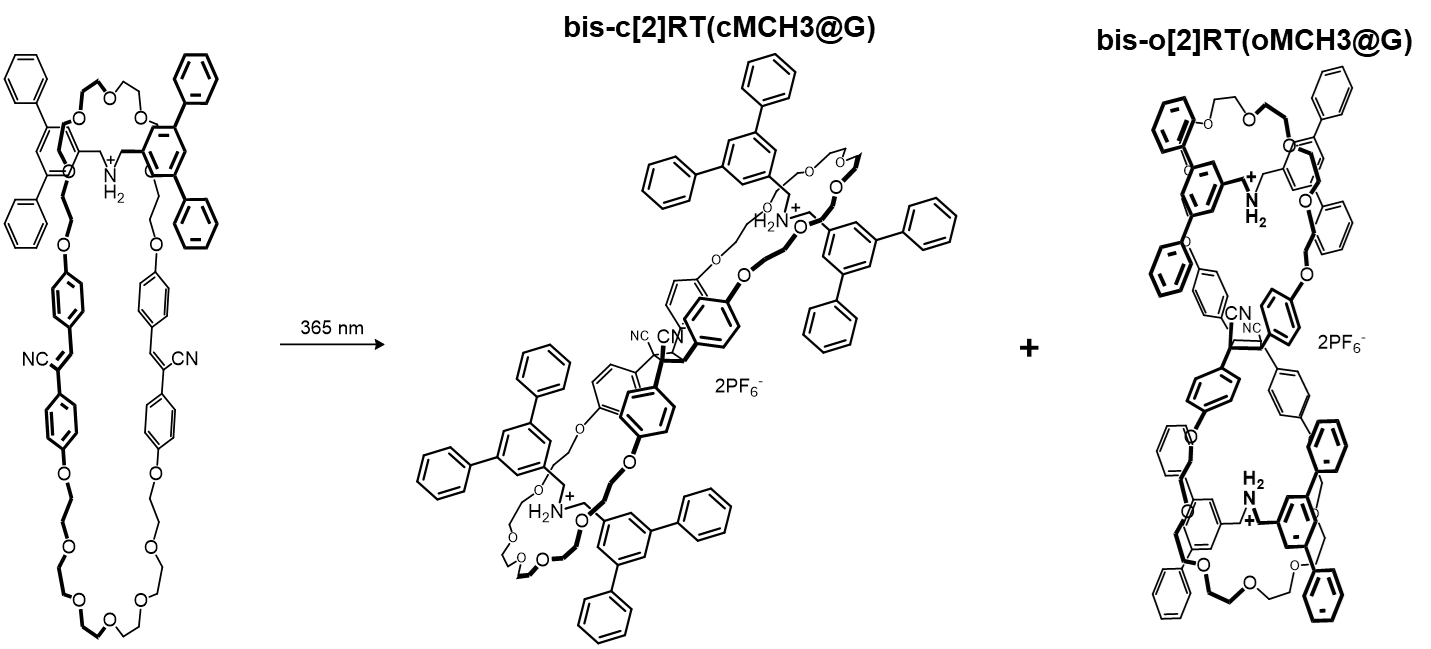
**

**Scheme S8.** Synthesis of **bis-c[2]RT(cMCH3@G)** and **bis-oRT(oMCH3@G)**

**Synthesis of 1**

3,5-Dibromobenzonitrile (1.5 g, 5.7 mmol), phenylboronic acid (1.75 g, 14.4 mmol) were dissolved in a mixture solvent of 1,2-dimethoxyethane and water (70 mL, v/v = 5/1). Pd(PPh_3_)_4_ (664 mg, 0.57 mmol) and Na_2_CO_3_ (1.83 g, 17.3 mmol)were added to the mixture under bubbling by N_2_. The mixture was reflux at 90 °C overnight. After cooling to room temperature, CH_2_Cl_2_ was added to the mixture and washed by water and then brine. The organic phase was separated, dried over Na_2_SO_4_ and evaporated to remove solvents under reduced pressure. The crude was purified through silica column chromatography (Hexane/Ethyl acetate) to give **1** as a white solid (Yield: 1.23 g, 84%). ^1^H NMR (396 MHz, CHLOROFORM-*D*) δ 7.99 (t, *J* = 1.8 Hz, 1H), 7.82 (d, *J* = 1.8 Hz, 2H), 7.66 – 7.56 (m, 4H), 7.52 – 7.46 (m, 4H), 7.45 – 7.39 (m, 2H).

Data was consistent with those reported in the literature ^1^.

^1^H NMR (400 MHz, CHCl₃-d, 298 K) spectrum of **1**


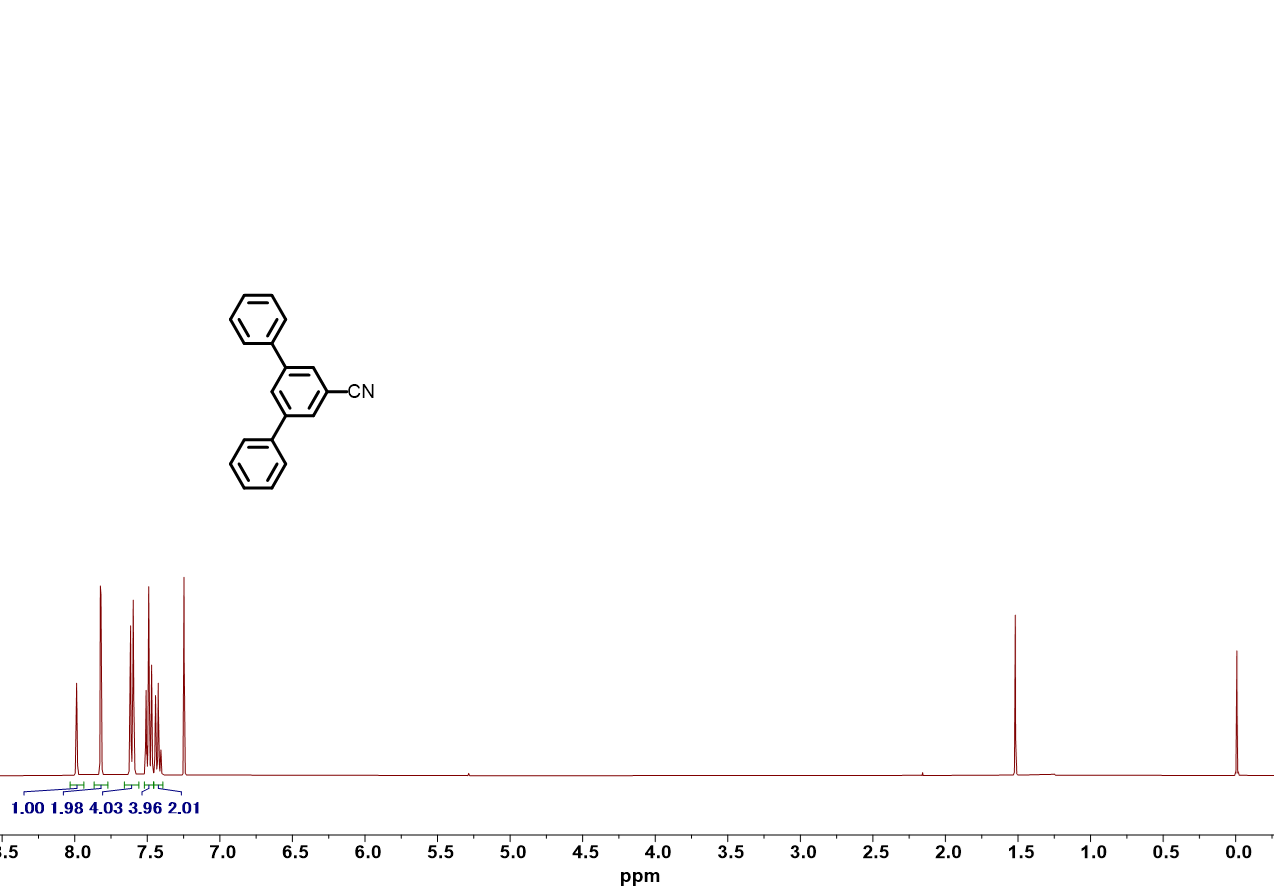


**Synthesis of 2**

**1** (1 g, 3.9 mmol) was dissolved in dry THF (20 mL). LiAlH_4_ (0.59 g, 15.7 mmol) was added to the solution in portions under ice bath. The reaction mixture was stirred at room temperature for 4 h. The reaction was quenched by dropwise addition of water under ice cooling until gas evolution ceased. The resulting mixture was diluted with CH₂Cl₂ (60 mL) and filtered. The filtrate was dried over Na₂SO₄, filtered, and concentrated under reduced pressure to give **2** as a white solid (0.94 g, 94%).^1^H NMR (400 MHz, CHLOROFORM-D) δ 7.69 (t, *J* = 1.7 Hz, 1H), 7.68 – 7.62 (m, 4H), 7.53 (dt, *J* = 1.7, 0.6 Hz, 2H), 7.49 – 7.42 (m, 4H), 7.40 – 7.33 (m, 2H), 4.01 (s, 2H). ^13^C NMR (126 MHz, CHLOROFORM-*D*) δ 144.49, 142.22, 141.25, 128.89, 127.52, 127.39, 125.06, 124.84, 46.76. HR-MS (ESI): calcd for , [C_19_H_17_N+H]^+^ , m/z = 260.1434, found m/z = 260.1436.

^1^H NMR (400 MHz, CHCl₃-d, 298 K) spectrum of **2**


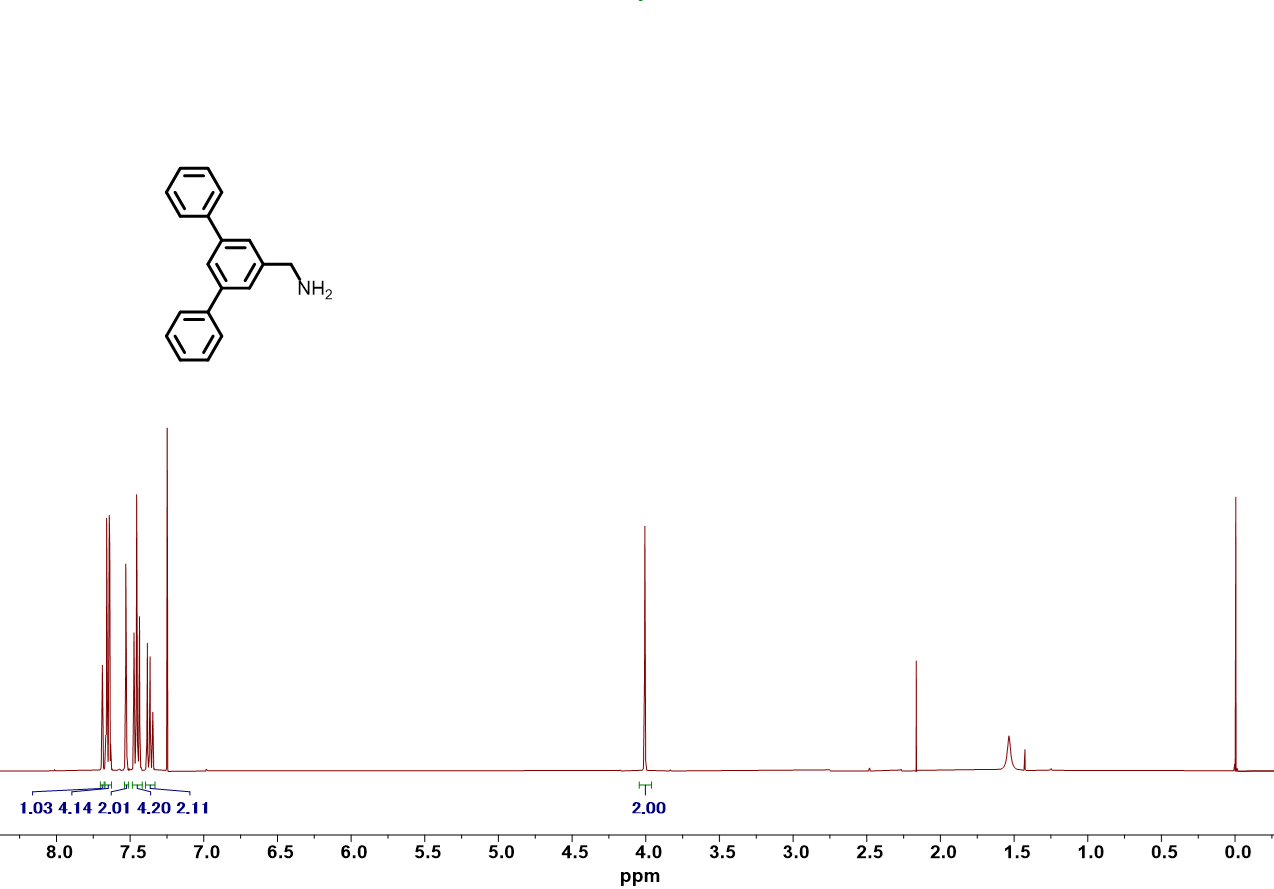


^13^C NMR (125 MHz, CHCl₃-d, 298 K) spectrum of **2**

**
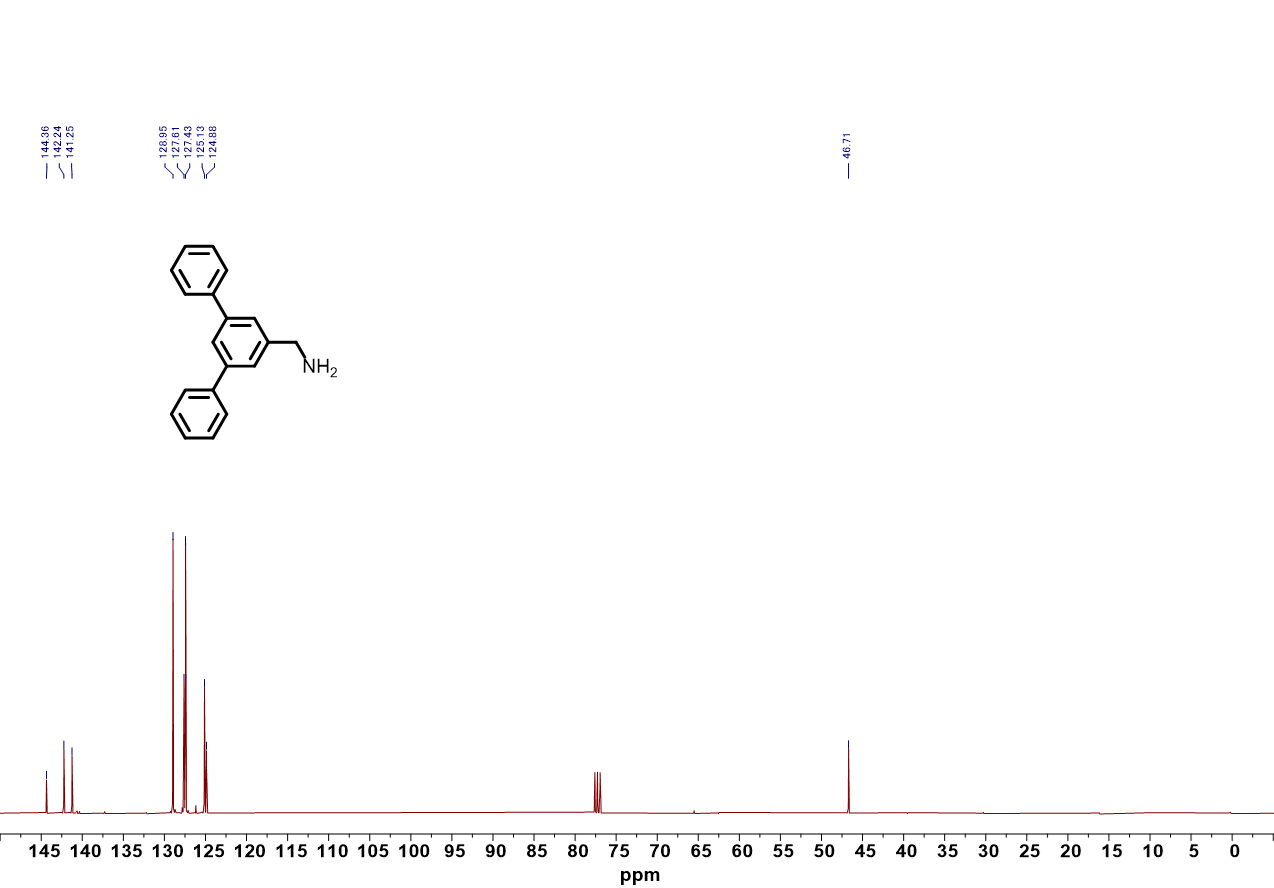
**

**Synthesis of 3**

3,5-Dibromobenzaldehyde (1.5 g, 5.7 mmol), phenylboronic acid (1.75 g, 14.4 mmol) were dissolved in a mixture solvent of 1,2-dimethoxyethane and water (v/v = 5/1). Pd(PPh_3_)_4_ (664 mg, 0.57 mmol) and Na_2_CO_3_ (1.83 g, 17.3 mmol)were added to the mixture under bubbling by N_2_. The mixture was reflux at 90 °C overnight. After cooling to room temperature, dichloromethane was added to the mixture and washed by water and then brine. The organic phase was separated, dried over Na_2_SO_4_, filtered and evaporated to remove solvents under reduced pressure. The crude was purified through silica column chromatography (Hexane/Ethyl acetate) to give **3** as a white solid (Yield: 1.23 g, 89%). ^1^H NMR (500 MHz, CHLOROFORM-*D*) δ 10.15 (s, 1H), 8.14 – 7.95 (m, 3H), 7.72 – 7.61 (m, 4H), 7.57 – 7.45 (m, 4H), 7.45 – 7.37 (m, 2H).

Data was consistent with those reported in the literature ^2^.

^1^H NMR (500 MHz, CHCl₃-d, 298 K) spectrum of **3**


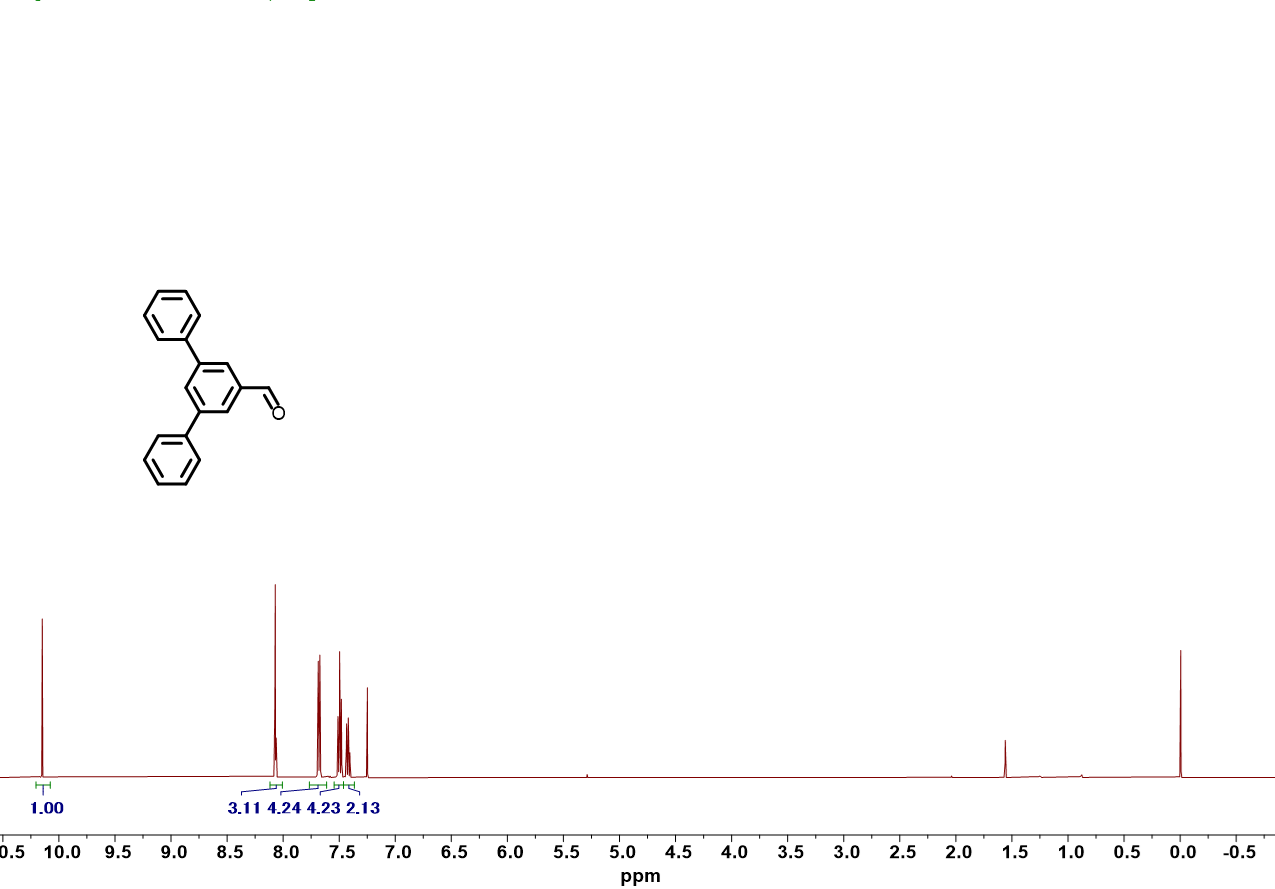


**Synthesis of 4**


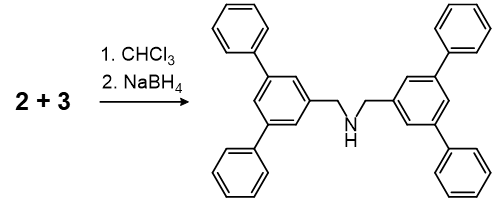


**2** (0.7 g, 2.7 mmol) and **3** (0.697 g, 2.7 mmol) were dissolved in dry CHCl_3_ (20 mL) and stirred at room temperature overnight. The solvent was removed under reduced pressure, and the residue was redissolved in a mixture solvent of THF and MeOH (30 mL, v/v = 2/1). NaBH_4_ (0.41 g, 10.8 mmol) was added to the solution in portions, and the mixture was stirred at room temperature for 3 h. The reaction was quenched by addition of water, followed by extraction with CH_2_Cl_2_ and washing with water and the brine. The organic layer was separated, dried over Na₂SO₄, filtered, and concentrated under reduced pressure to give compound **4** as a white solid (Yield: 1.21 g, 90%). ^1^H NMR (400 MHz, CHLOROFORM-*D*) δ 7.70 (t, *J* = 1.8 Hz, 2H), 7.67 – 7.64 (m, 8H), 7.59 (d, *J* = 1.8 Hz, 4H), 7.49 – 7.39 (m, 8H), 7.36 (m, 4H), 4.00 (s, 4H). ^13^C NMR (126 MHz, CHLOROFORM-*D*) δ 142.05, 141.42, 141.21, 128.89, 127.52, 127.40, 126.20, 125.00, 53.25. HR-MS (ESI): calcd for , [C_38_H_32_N+H]^+^ , m/z = 502.2530, found m/z =502.2534.

^1^H NMR (400 MHz, CHCl₃-d, 298 K) spectrum of **4**


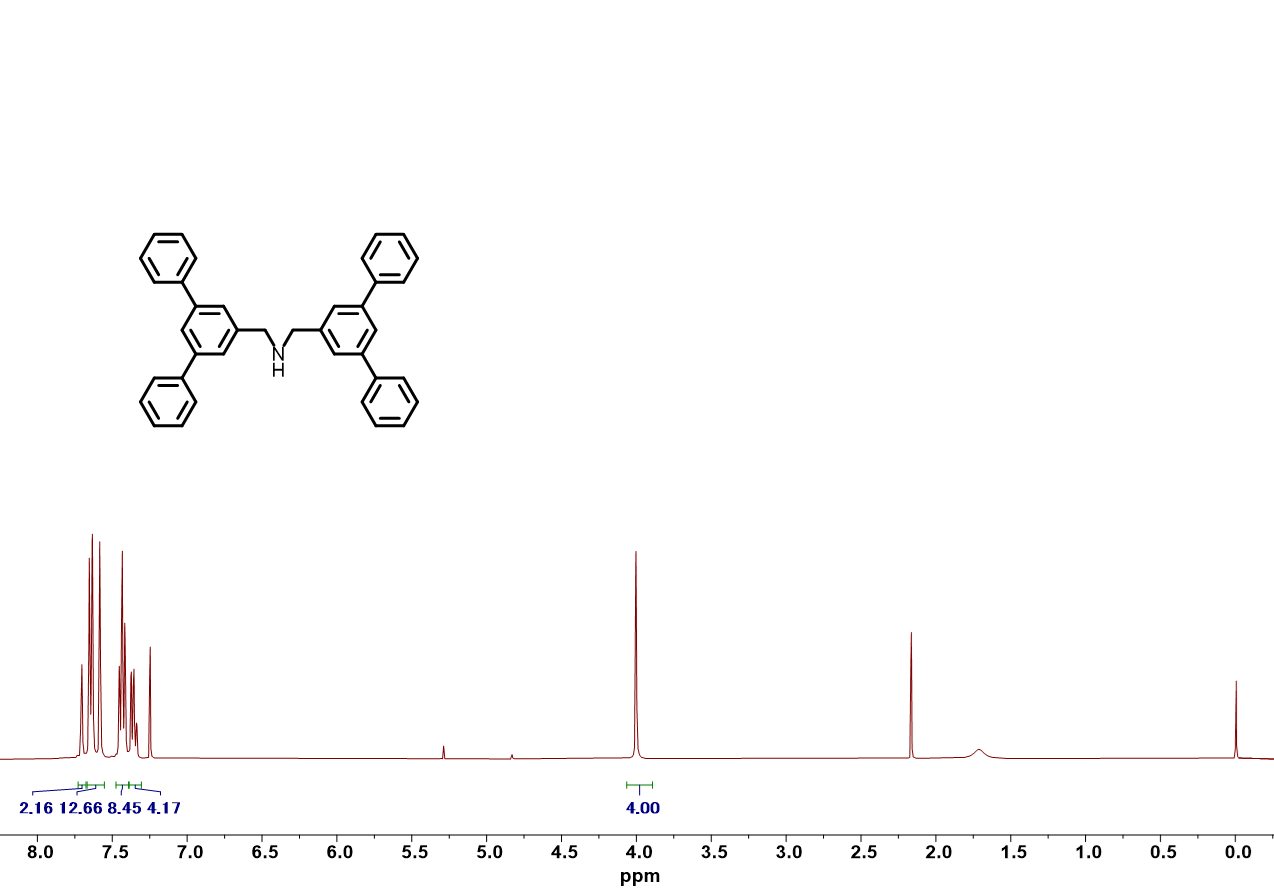


^13^C NMR (125 MHz, CHCl₃-d, 298 K) spectrum of **4**


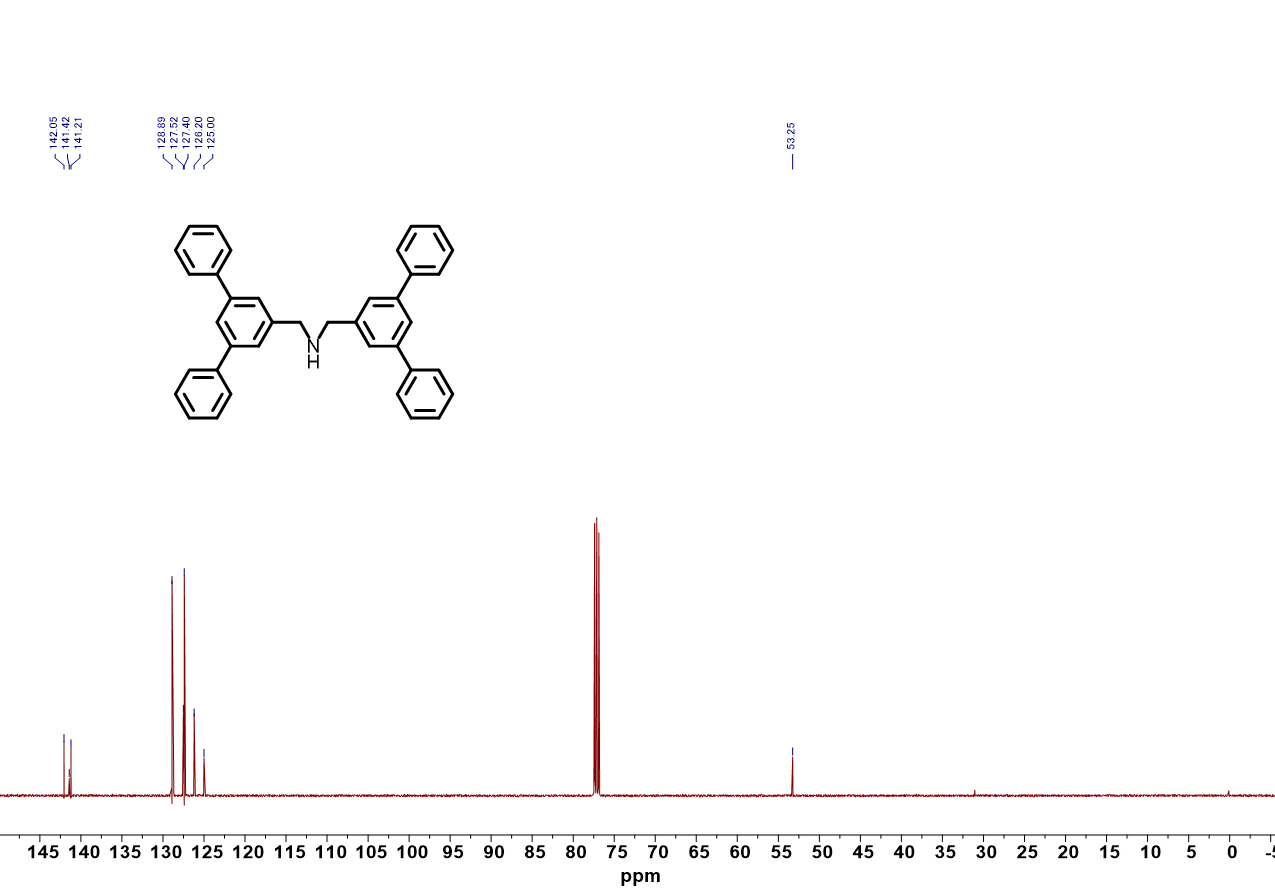


**Synthesis of G**

**4** (1 g, 2 mmol) was dissolved in CH_2_Cl_2_ (40 mL). 3 mL concentrated HCl was added to the solution and stirred at room temperature overnight. The white precipitate was collected via suction filtration, washed by water. The resulting solid was dispersed in acetone (50 mL), followed by addition of saturated aqueous solution of NH_4_PF_6_ (10 mL). The mixture was stirred at room temperature overnight and then concentrated under reduced pressure without heating to remove the acetone. The resulting suspension was diluted with deionized water and filtered by suction filtration. The solid was collected and dried under reduced pressure to obtain **G** as a white solid (Yield: 0.92 g, 71%). ^1^H NMR (400 MHz, ACETONE-*D*_6_) δ 8.85 (s, 2H), 8.00 (t, *J* = 1.7 Hz, 2H), 7.90 (d, *J* = 1.7 Hz, 4H), 7.80 – 7.67 (m, 8H), 7.51 – 7.44 (m, 8H), 7.43 – 7.37 (m, 4H), 4.92 (s, 4H). ^13^C NMR (126 MHz, ACETONE-*D*_6_) δ 142.57, 140.00, 132.53, 129.10, 128.09, 127.80, 127.13, 126.59, 52.05. HR-MS (ESI): calcd for , [C_38_H_33_N]^+^ , m/z = 502.2530, found m/z =502.2531.

^1^H NMR (400 MHz, CHCl₃-d, 298 K) spectrum of **G**


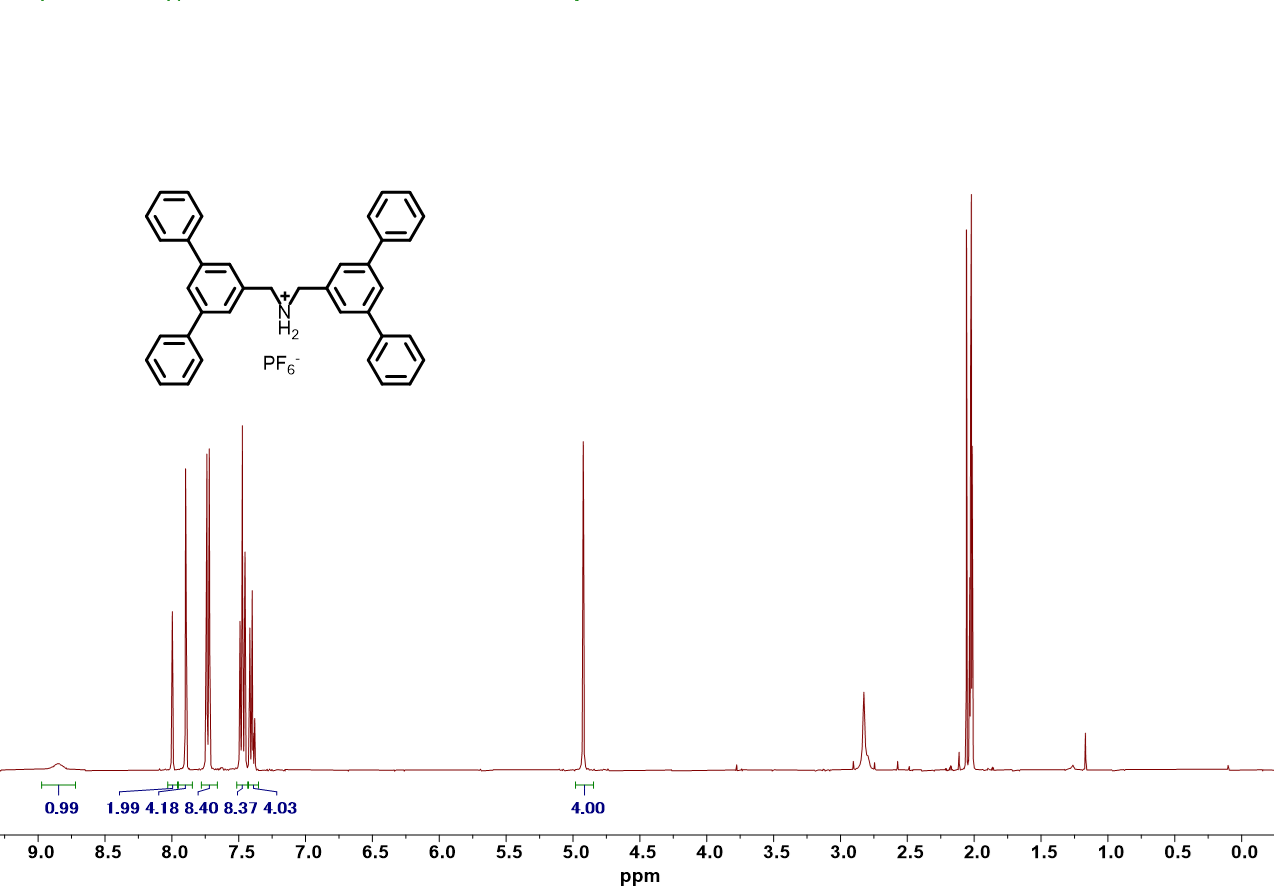


^13^C NMR (125 MHz, CHCl₃-d, 298 K) spectrum of **2**


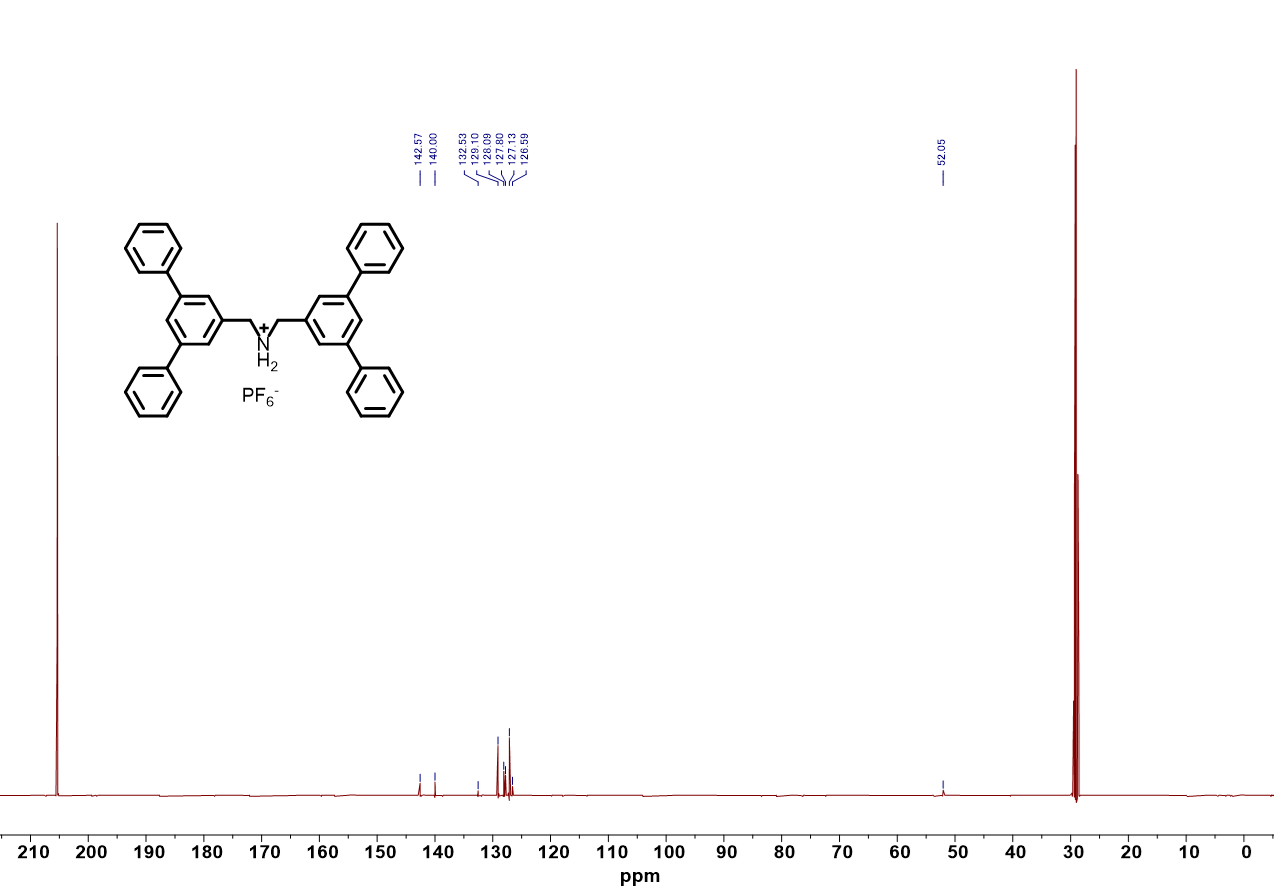


**Synthesis of 5**

To a solution of 4-hydroxybenzyl cyanide (1 g, 7.51 mmol) and allyl bromide (0.91 g, 7.51 mmol) in DMF (30 mL) was added K_2_CO_3_ (2.07 g, 15.02 mmol). After bubbling by N_2_ for 10 minutes, the mixture was stirred at 70 °C for 2 hours. The reaction mixture was poured into water and extracted with diethyl ether. The organic phase was washed with water (2x) and brine (1x), dried over MgSO_4_ and evaporated. **26** was obtained as orange oil without further purification (Yield: 1.21 g, 93%). ^1^H NMR (500 MHz, CHLOROFORM-*D*) δ 7.21 (d, *J* = 8.6, 2H), 6.90 (d, *J* = 8.6, 2H), 6.03 (m, 1H), 5.40 (dp, *J* = 17.2, 1.5 Hz, 1H), 5.29 (dp, *J* = 10.4, 1.3 Hz, 1H), 4.53 (dq, *J* = 5.4, 1.4 Hz, 2H), 3.67 (s, 2H).

Data was consistent with those reported in the literature ^3^.

^1^H NMR (500 MHz, CHCl₃-d, 298 K) spectrum of **5**


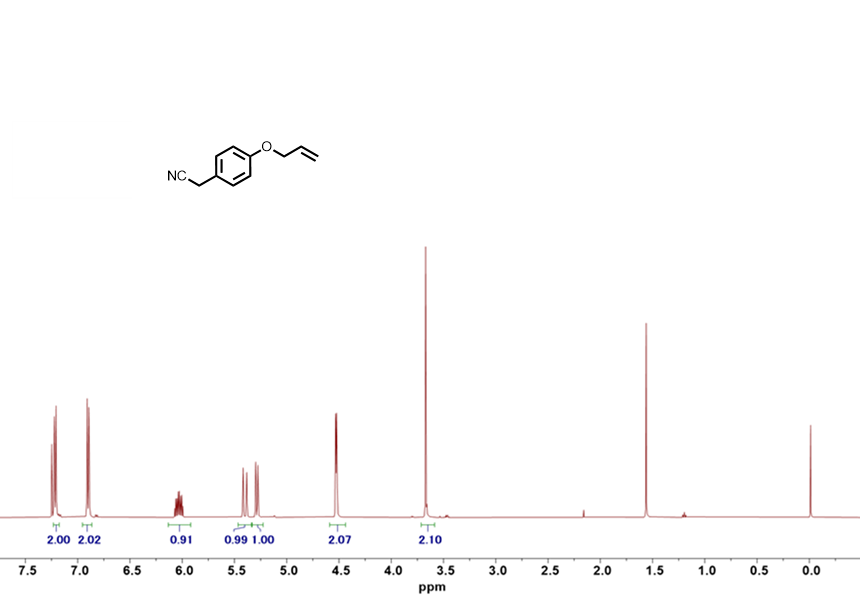


**Synthesis of 6**

Hexaethylene glycol (3 g, 10.6 mmol) and p-toluenesulfonyl chloride (4.25 g, 22.3 mmol) were dissolved in DCM (30 mL). KOH (4.76 g, 85 mmol) was added into the solution under ice bath in portions over 1 h. The ice bath was removed after addition and the mixture was stirred at room temperature overnight. Then the reaction mixture was poured into water and extracted with CH_2_Cl_2_ (3x). The combined organic phase was washed with water (2x) and brine (1x), dried over Na_2_SO_4_ and evaporated. **6** was obtained as a colorless oil without further purification (Yield: 5.65 g, 90%). ^1^H NMR (500 MHz, CHLOROFORM-*D*) δ 7.78 (d, *J* = 8.3 Hz, 4H), 7.33 (d, *J* = 8.3 Hz, 4H), 4.14 (t, *J* = 9.7 Hz, 4H), 3.67 (t, *J* = 9.7 Hz, 4H), 3.60 (s, 8H), 3.57 (s, 8H), 2.43 (s, 6H).

Data was consistent with those reported in the literature ^4^.

^1^H NMR (500 MHz, CHCl₃-d, 298 K) spectrum of **6**


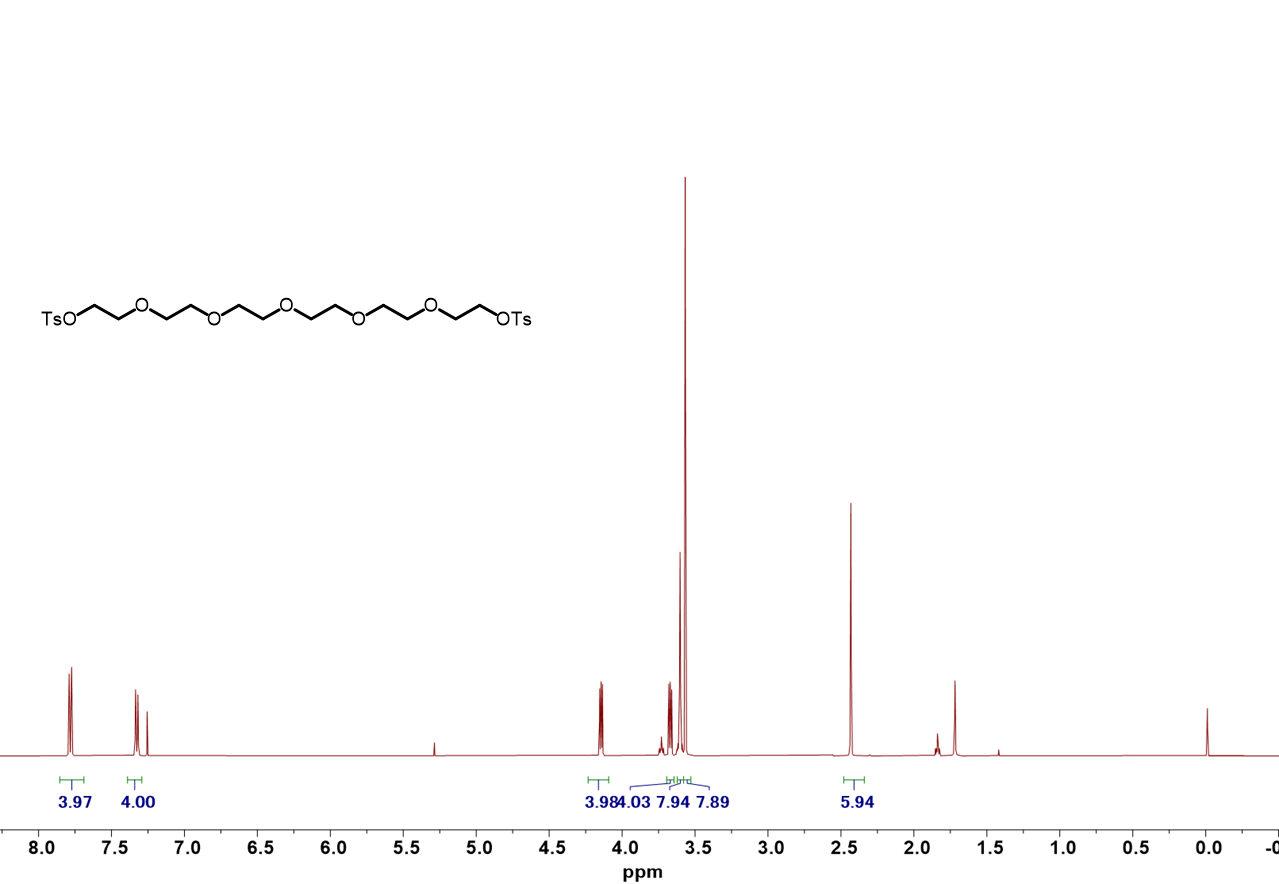


**Synthesis of 7**


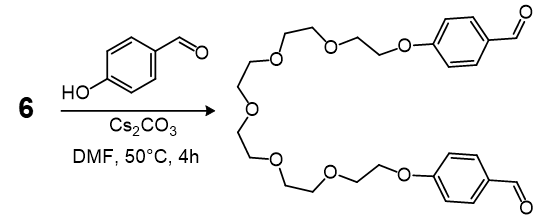


To a solution of **6** (1 g, 1.69 mmol) and 4-hydroxybenzaldehyde (0.41 g, 3.39 mmol) in DMF (40 mL) was added Cs_2_CO_3_ (1.65 g, 5.08 mmol). After stirring at 50 °C for 2 h, the reaction mixture was poured into water and extracted with ethyl acetate. The organic phase was washed with water (2x) and brine (1x), dried over MgSO_4_ and evaporated. **7** was obtained as a light-yellow oil without further purification (Yield: 0.83 g, 93%). ^1^H NMR (500 MHz, CHLOROFORM-*D*) δ 9.86 (s, 2H), 7.81 (d, *J* = 8.8 Hz, 4H), 7.00 (d, *J* = 8.8 Hz, 4H), 4.28 – 4.08 (m, 4H), 3.91 – 3.81 (m, 4H), 3.76 – 3.52 (m, 16H).

Data was consistent with those reported in the literature ^5^.

^1^H NMR (500 MHz, CHCl₃-d, 298 K) spectrum of **7**


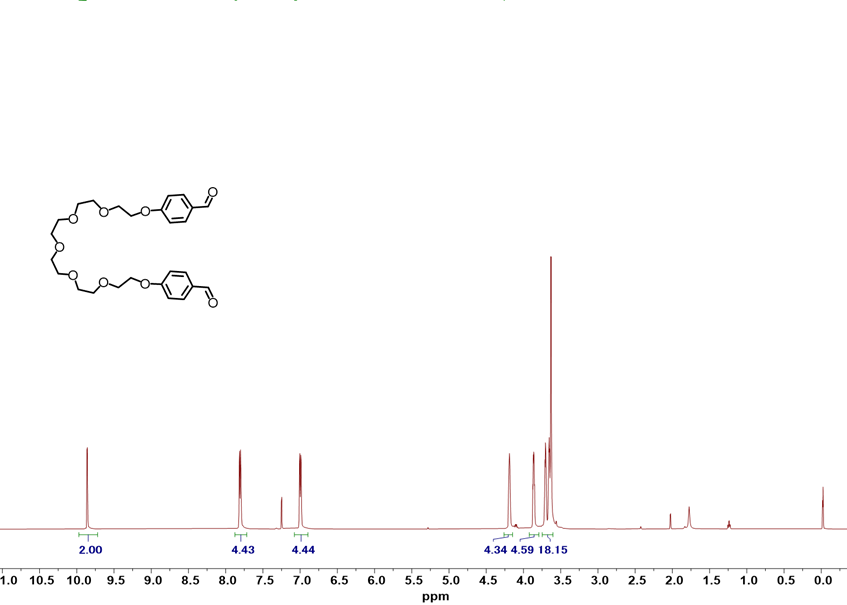


**Synthesis of 8**


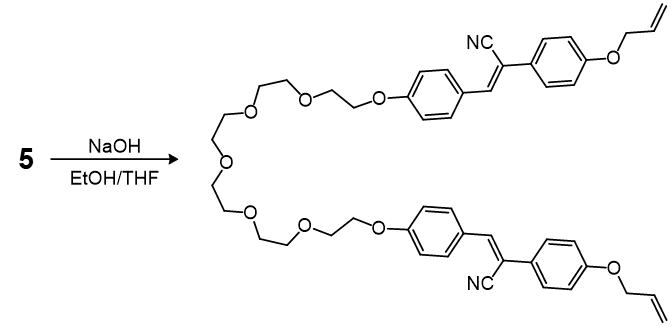


To a solution of **5** (1.23 g, 7.1 mmol) and **7** (1.66 g, 3.38 mmol) in dry ethanol (20 mL) and dry THF (15 mL) was added NaOH (0.14 g, 1.69 mmol) under N_2_ atmosphere. After stirring at room temperature overnight, the reaction mixture was poured into water and extracted with ethyl acetate. The organic phase was washed with water (1x) and brine (1x), dried over MgSO_4_ and evaporated. The crude was purified via the silica column chromatography (CH_2_Cl_2_/Ethyl acetate). **8** was obtained as yellow oil (Yield: 0.96 g, 70%). ^1^H NMR (500 MHz, CHLOROFORM-*D*) δ 7.82 (d, *J* = 8.9 Hz, 4H), 7.55 (d, *J* = 8.8 Hz, 4H), 7.33 (s, 2H), 6.96 (m, 8H), 6.05 (m, 2H), 5.42 (dq, *J* = 17.2, 1.6 Hz, 1H), 5.30 (dq, *J* = 10.5, 1.4 Hz, 1H), 4.57 (dt, *J* = 5.3, 1.5 Hz, 2H), 4.17 (t, *J* = 9.7 Hz, 4H), 3.86 (t, *J* = 9.7 Hz, 4H), 3.73 – 3.63 (m, 16H). ^13^C NMR (101 MHz, CHLOROFORM-D) δ 160.39, 159.16, 140.03, 132.92, 130.95, 127.55, 127.13, 126.93, 118.75, 118.11, 115.23, 115.01, 108.39, 70.96, 70.72, 70.67, 69.66, 68.99, 68.07, 67.62. HR-MS (ESI): calcd for , [C_48_H_52_N_2_O_9_+Na]^+^ , m/z = 823.3571, found m/z = 823.3578.

^1^H NMR (500 MHz, CHCl₃-d, 298 K) spectrum of **8**


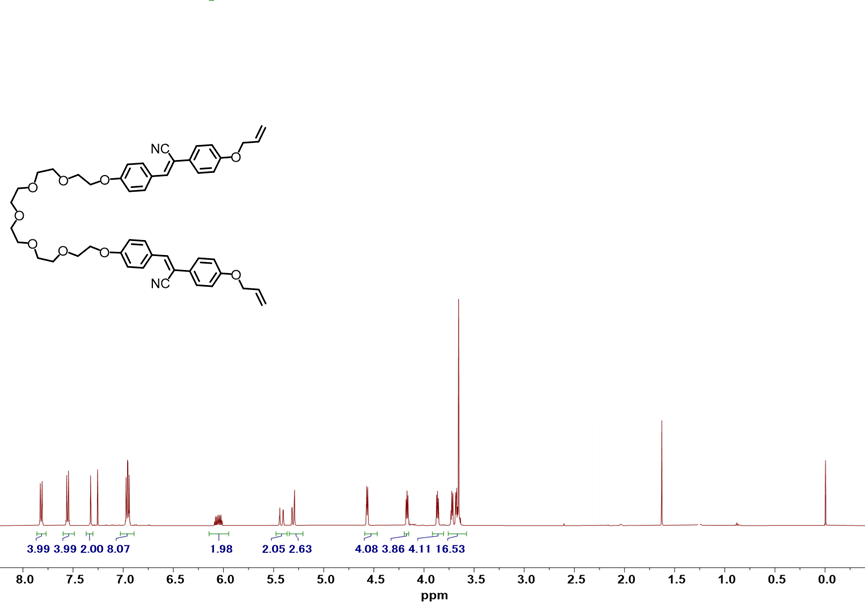


^13^C NMR (101 MHz, CHCl₃-d, 298 K) spectrum of **8**


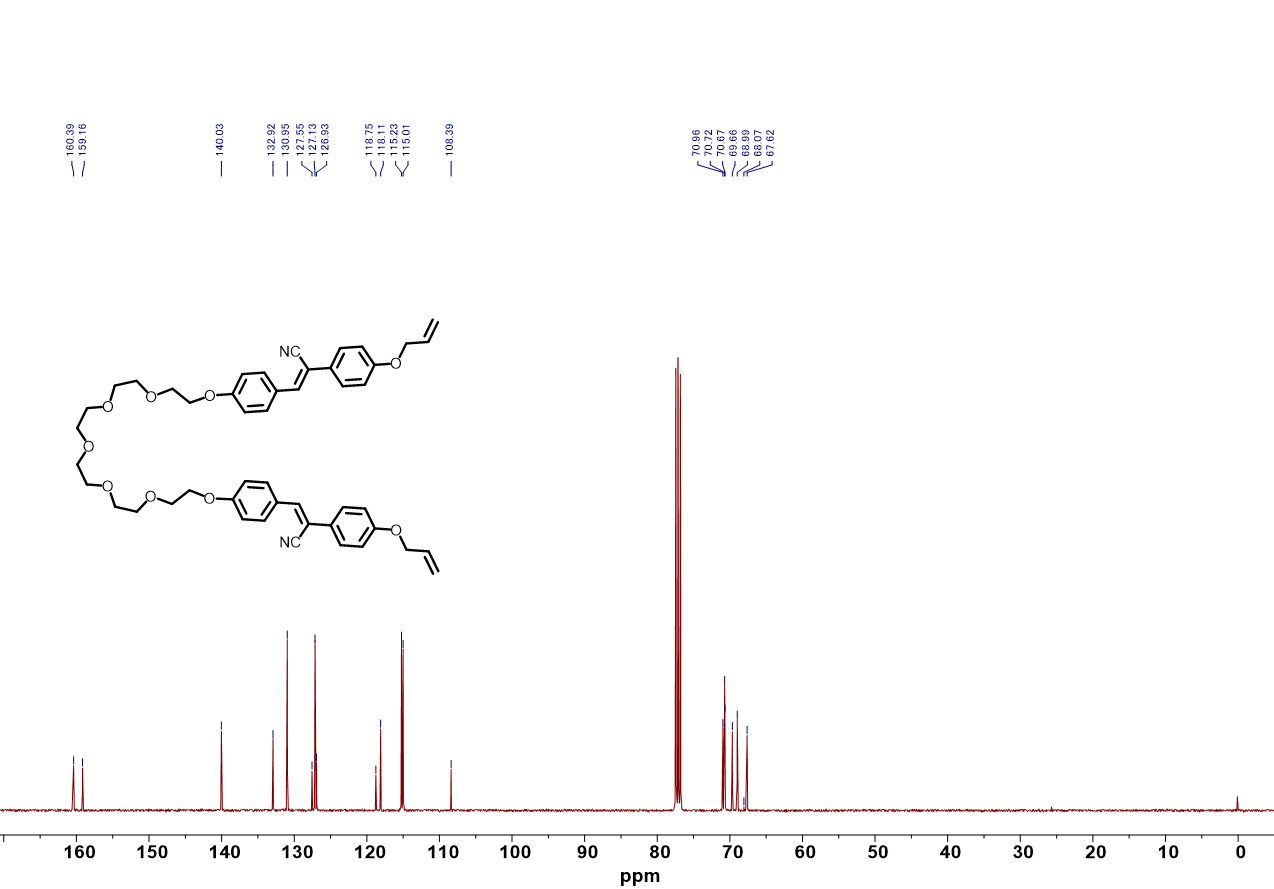


**Synthesis of 9**


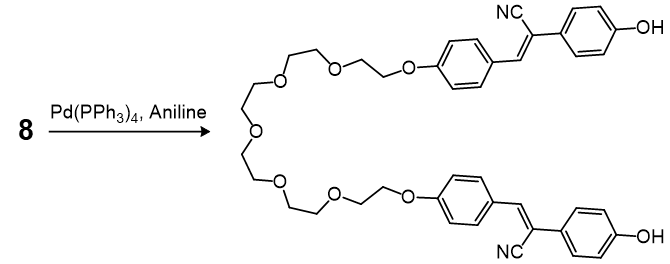


To a solution of **8** (0.96 g, 1.20 mmol) and aniline (126 mg, 1.32 mmol) in dry THF (10 mL) was added Pd(PPh_3_)_4_ (72 mg, 0.06 mmol) under N_2_ atmosphere. After stirring at room temperature overnight, the reaction mixture was poured into water and extracted with CH_2_Cl_2_. The organic phase was washed with water (1x), brine (1x), dried over MgSO_4_ and evaporated. The crude was purified via the silica column chromatography (CH_2_Cl_2_/Ethyl acetate). **9** was obtained as a light-yellow oil (Yield: 0.55 g, 64%). ^1^H NMR (500 MHz, CHLOROFORM-*D*) δ 7.67 (d, *J* = 8.8 Hz, 4H), 7.32 (d, *J* = 8.7 Hz, 4H), 7.12 (s, 2H), 6.83 (d, *J* = 8.8, 4H), 6.74 (d, *J* = 8.9 Hz, 4H), 3.99 (t, *J* = 9.6, 4H), 3.80 (t, *J* = 9.6, 5H), 3.72 – 3.63 (m, 16H). ^13^C NMR (101 MHz, CHLOROFORM-D) δ 160.07, 157.20, 139.41, 130.82, 127.05, 126.75, 126.45, 118.83, 116.16, 114.74, 108.11, 70.71, 70.65, 70.55, 70.48, 69.67, 67.26. HR-MS (ESI): calcd for , [C_42_H_44_N_2_O_9_+Na]^+^ , m/z = 743.2945, found m/z = 743.2947.

^1^H NMR (500 MHz, CHCl₃-d, 298 K) spectrum of **9**


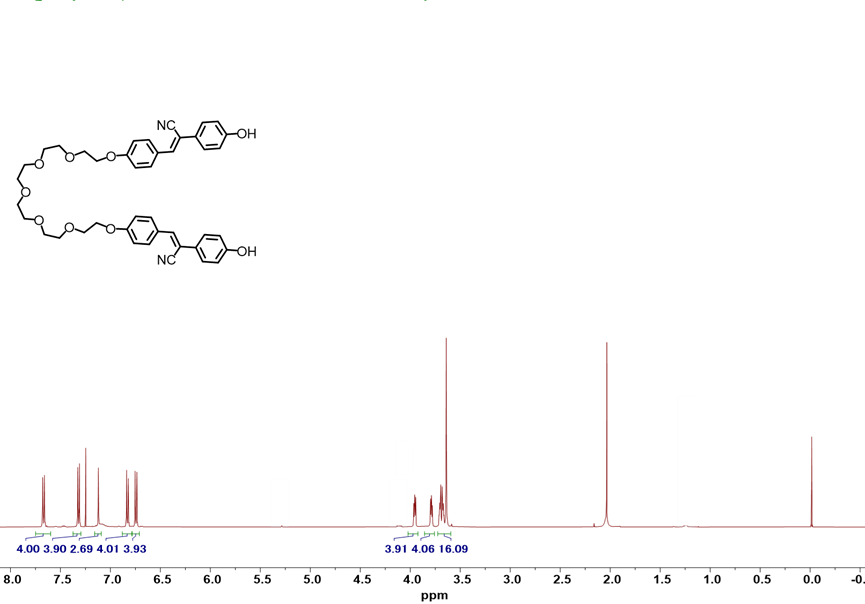


^13^C NMR (400 MHz, CHCl₃-d, 298 K) spectrum of **9**


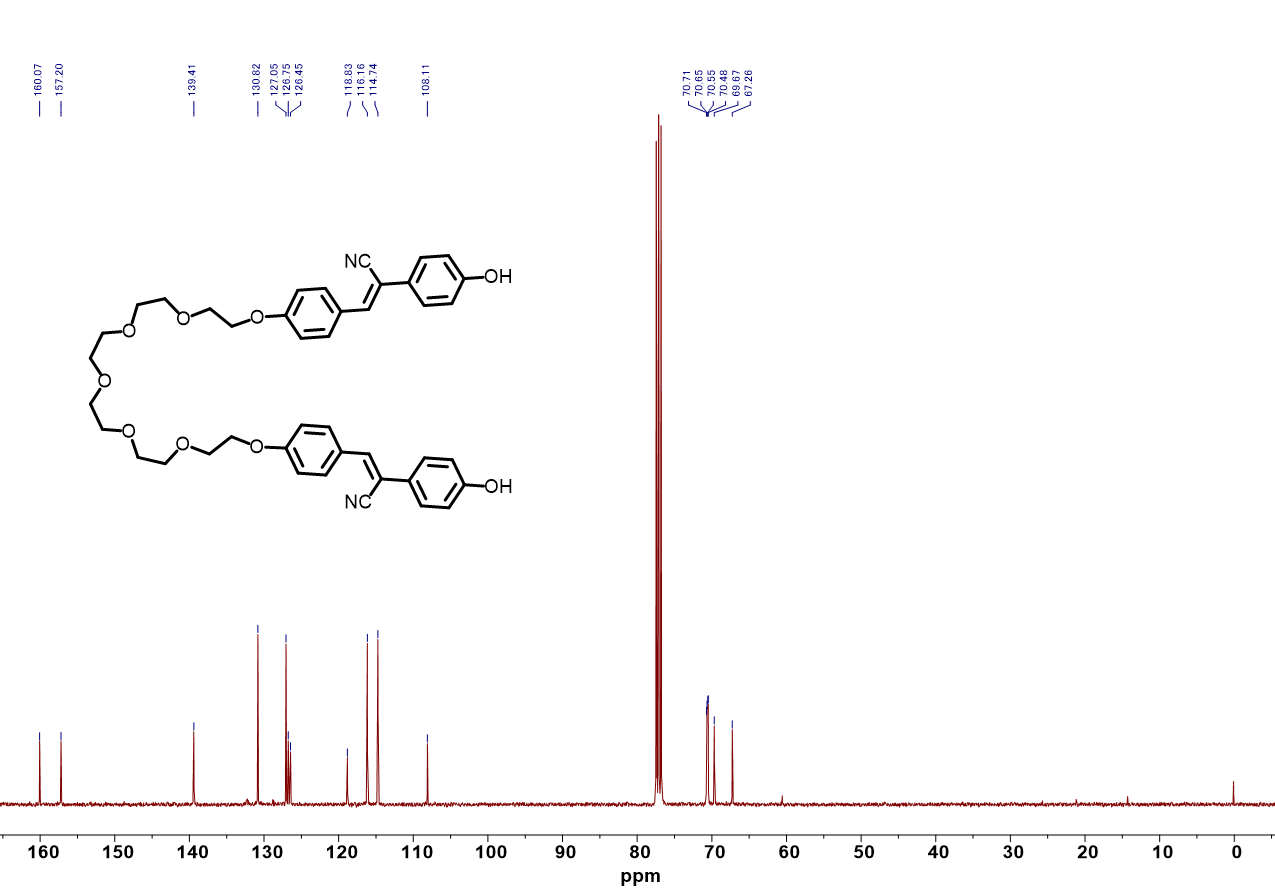


**Synthesis of 10**

To a solution of catechol (2 g, 18.2 mmol) and 6-bromohexan-1-ol (14.1 g, 45.4 mmol) in DMF was added K_2_CO_3_ under N_2_ atmosphere. After stirring at 90 °C overnight, the reaction mixture was poured into water and extracted with ethyl acetate. The organic phase was washed with water (3 x) and brine (1x), dried over MgSO_4_ and evaporated. The crude was purified via silica column chromatography (CH_2_Cl_2_/Ethyl acetate). **10** were obtained as white solid (Yield: 2.93 g, 52%). ^1^H NMR (396 MHz, CHLOROFORM-*D*) δ 6.87 (s, 4H), 3.98 (t, *J* = 6.4 Hz, 4H), 3.63 (t, *J* = 6.6 Hz, 4H), 1.89 – 1.75 (m, 4H), 1.61 – 1.33 (m, 12H).

Data was consistent with those reported in the literature ^6^.

^1^H NMR (400 MHz, CHCl₃-d, 298 K) spectrum of **10**


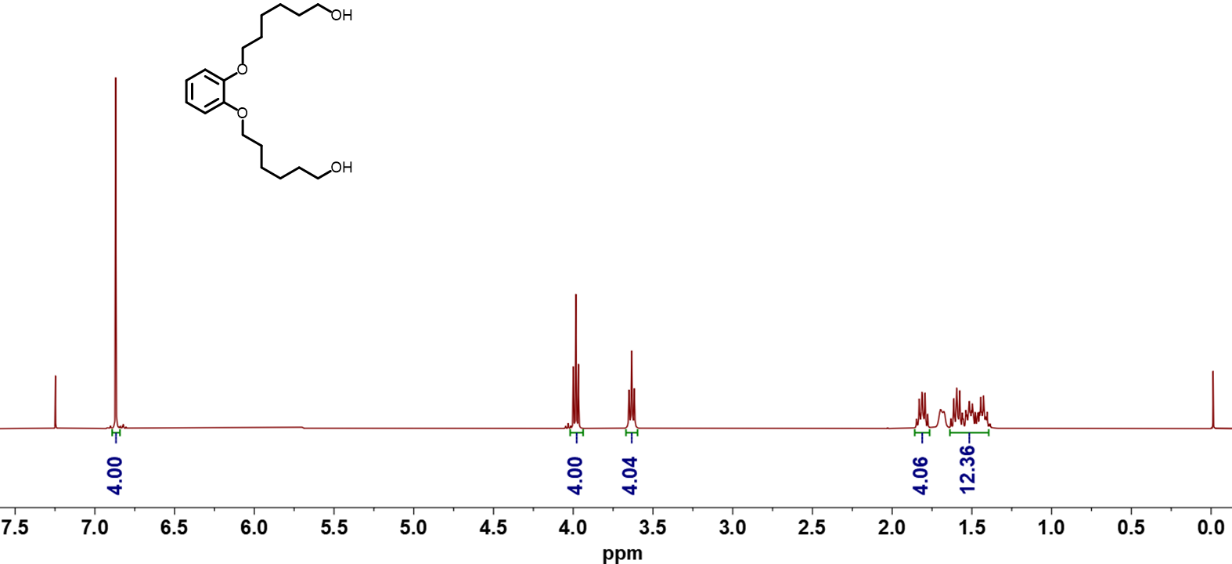


**Synthesis of 11**


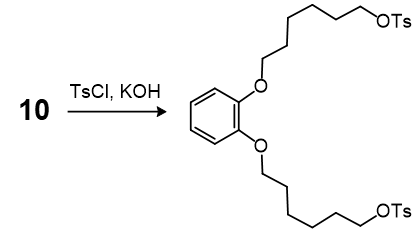


**10** (1 g, 3.2 mmol) and p-toluenesulfonyl chloride (1.79 g, 6.75 mmol) were dissolved in diethyl ether (40 mL). KOH (1.45 g, 25.8 mmol) was added into the solution under ice bath in portions over 1 h. The ice bath was removed after addition and the mixture was stirred at room temperature overnight. Then the reaction mixture was poured into water and extracted with CH_2_Cl_2_ (3x). The combined organic phase was washed with water (2x) and brine (1x), dried over Na_2_SO_4_ and evaporated. **6** was obtained as a colorless oil without further purification (Yield: 4.07 g, 86%). ^1^H NMR (500 MHz, CHLOROFORM-*D*) δ 7.77 (d, *J* = 8.3 Hz, 4H), 7.32 (d, *J* = 7.9, 0.6 Hz, 4H), 6.93 – 6.78 (m, 4H), 4.01 (t, *J* = 6.4 Hz, 4H), 3.92 (t, *J* = 6.5 Hz, 4H), 1.78 – 1.72 (m, 4H), 1.68 – 1.63 (m, 4H), 1.45 – 1.32 (m, 8H). ^13^C NMR (126 MHz, CHLOROFORM-*D*) δ 149.19, 144.75, 133.37, 129.91, 127.96, 121.27, 114.28, 70.60, 69.02, 29.17, 28.91, 25.54, 25.26, 21.69. HR-MS (ESI): calcd for , [C_32_H_42_O_8_S_2_+Na]^+^ , m/z = 641.2219, found m/z = 641.2219.

^1^H NMR (500 MHz, CHCl₃-d, 298 K) spectrum of **11**


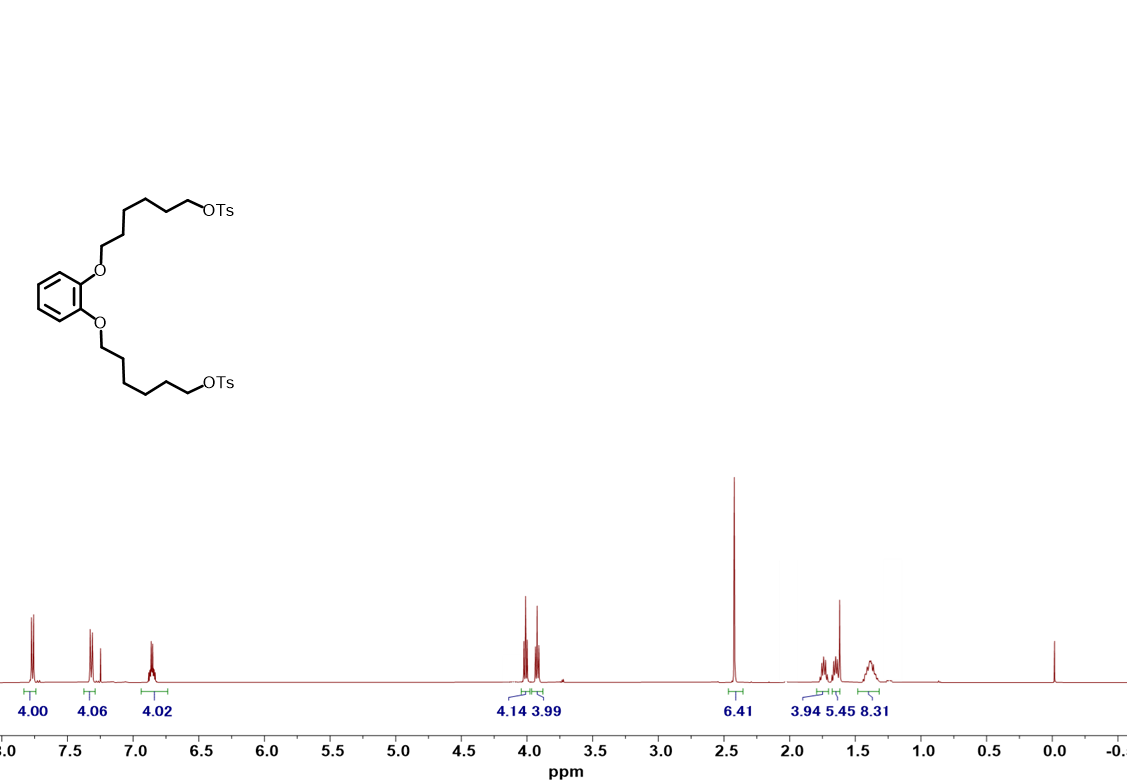


^13^C NMR (125 MHz, CHCl₃-d, 298 K) spectrum of **11**


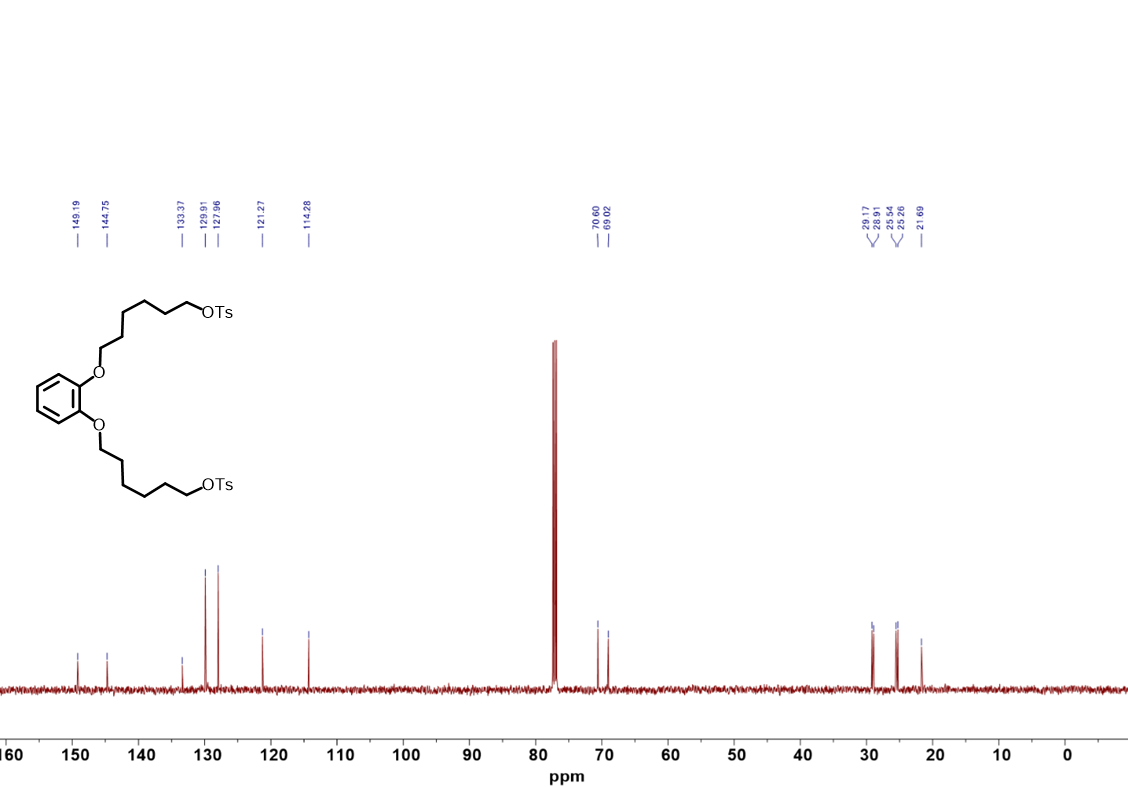


**Synthesis of 12**

Butane-1,4-diol (2 g, 22.2 mmol) and p-toluenesulfonyl chloride (8.88 g, 46.6 mmol) were dissolved in CH_2_Cl_2_ (60 mL). KOH (9.94 g, 17.8 mmol) was added into the solution under ice bath in portions over 1 h. The ice bath was removed after addition and the mixture was stirred at room temperature overnight. Then the reaction mixture was poured into water and extracted with CH_2_Cl_2_ (3x). The combined organic phase was washed with water (2x) and brine (1x), dried over Na_2_SO_4_ and evaporated. **6** was obtained as a white solid without further purification (Yield: 3.43 g, 86%). ^1^H NMR (396 MHz, CHLOROFORM-*D*) δ 7.82 – 7.68 (d, *J* = 6.5 Hz, 4H), 7.38 – 7.29 (d, *J* = 6.5 Hz, 4H), 3.99 – 3.96 (m, 4H), 2.44 (s, 6H), 1.70 – 1.67 (m, 4H).

Data was consistent with those reported in the literature^7^.

^1^H NMR (400 MHz, CHCl₃-d, 298 K) spectrum of **12**


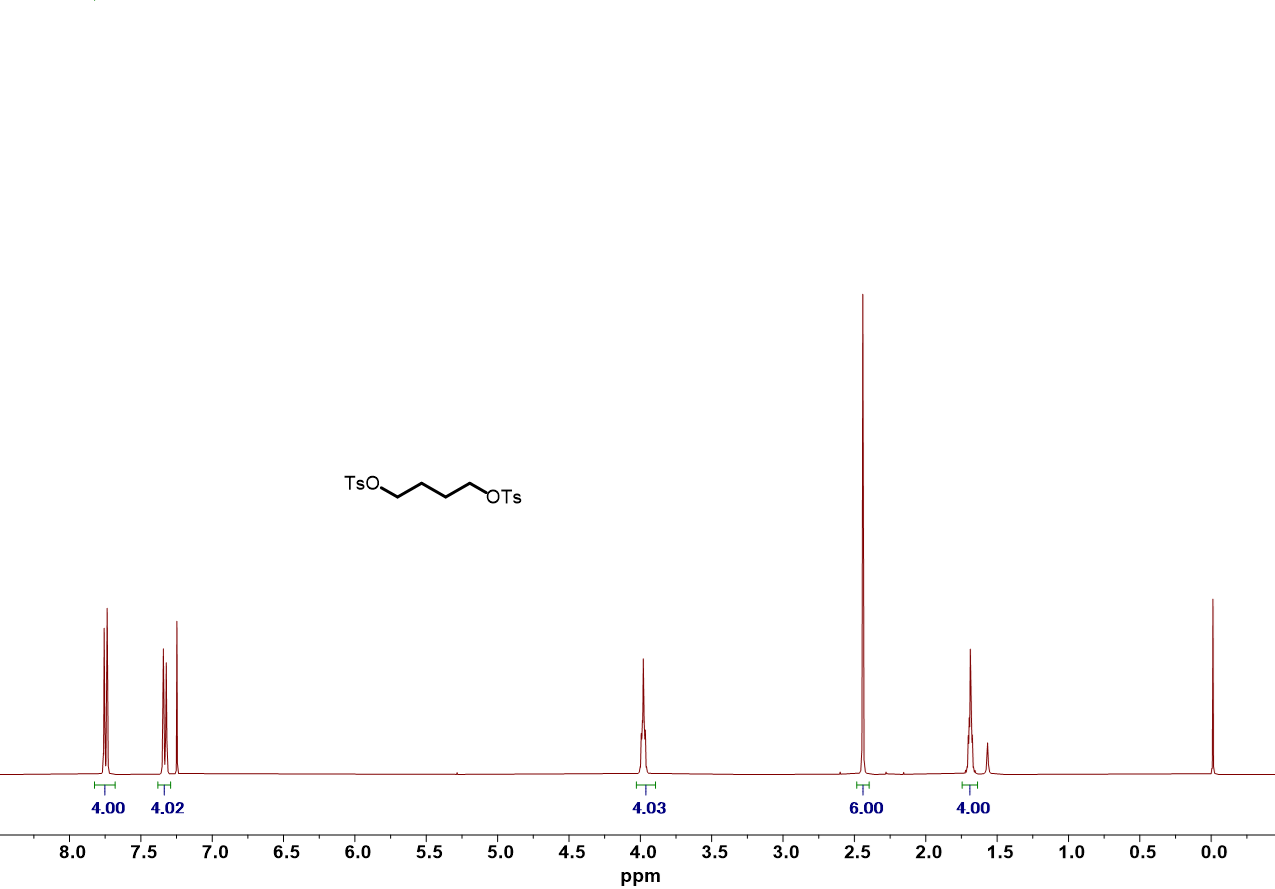


**Synthesis of MCH1**

**
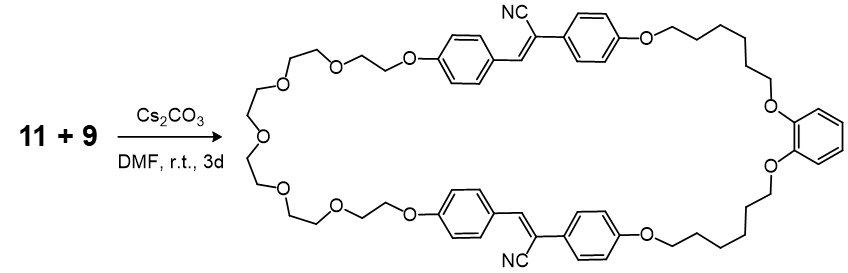
**

To a solution of **11** (0.6 g, 0.97 mmol) and **9** (0.7 g, 0.97 mmol) in DMF (80 mL) was added Cs_2_CO_3_ (1.89 g, 5.82 mmol). The mixture was stirred at room temperature for 3 days. Then the reaction mixture was poured into water and extracted with ethyl acetate. The organic phase was dried over MgSO_4_ and evaporated. The crude was purified via the silica column chromatography (CH_2_Cl_2_/ Ethyl acetate). **MCH1** was obtained as a yellow solid (Yield: 0.31 g, 32%). ^1^H NMR (500 MHz, CHLOROFORM-*D*) δ 7.68(d, *J* = 8.8 Hz, 4H), 7.39 (d, *J* = 8.8 Hz, 4H), 7.13 (s, 2H), 6.89 (s, 4H), 6.84 (d, *J* = 8.8 Hz, 4H), 6.78 (d, *J* = 8.8 Hz, 4H), 4.10 – 4.05 (t, *J* = 9.5 Hz, 4H), 4.01 (t, *J* = 11.9 Hz, 4H), 3.89 – 3.81 (m, 8H), 3.75 – 3.59 (m, 16H), 1.81 (m, 8H), 1.64 – 1.46 (m, 8H). ^13^C NMR (101 MHz, CHLOROFORM-*D*) δ 160.29, 159.63, 149.31, 139.57, 130.94, 126.95, 126.81, 121.20, 118.77, 114.81, 114.04, 108.09, 71.04, 70.86, 70.83, 69.65, 69.08, 68.08, 29.47, 26.36, 26.01. HR-MS (ESI): calcd for , [C_60_H_70_N_2_O_11_+Na]^+^ , m/z = 1017.4877, found m/z = 1017.4883.

^1^H NMR (500 MHz, CHCl₃-d, 298 K) spectrum of **MCH1**


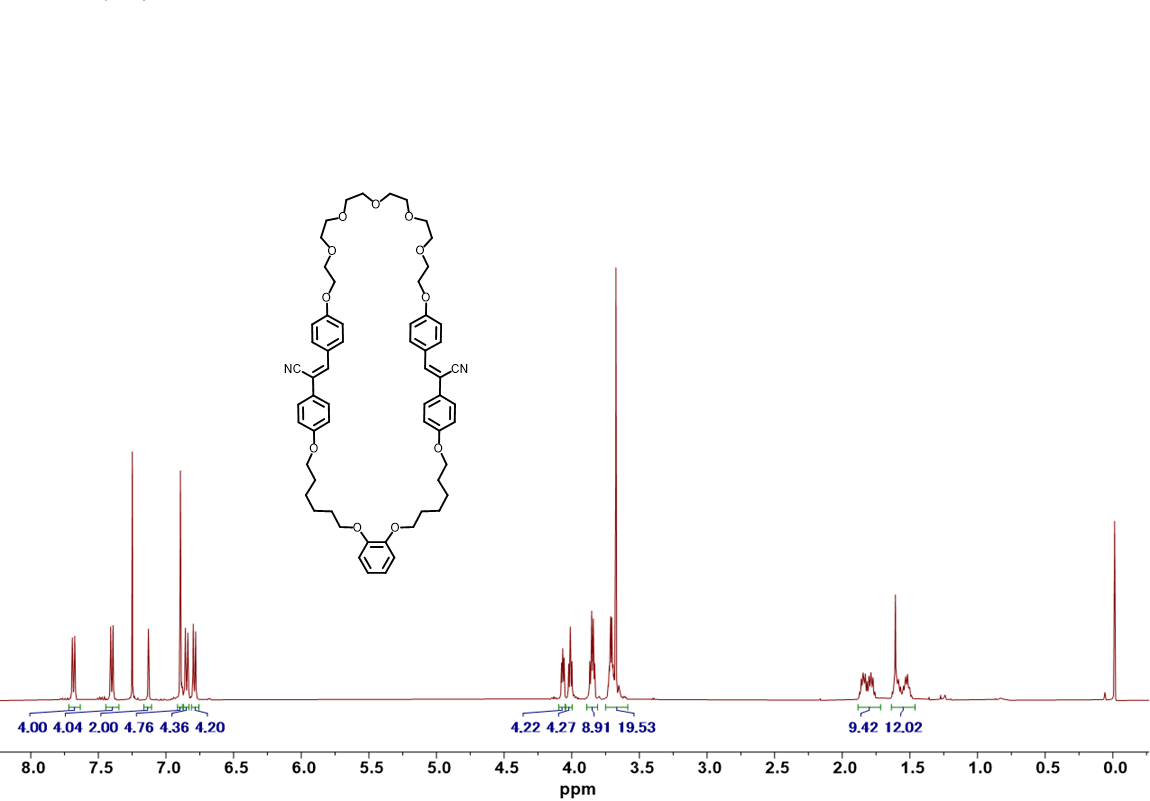


^13^C NMR (100 MHz, CHCl₃-d, 298 K) spectrum of **MCH1**


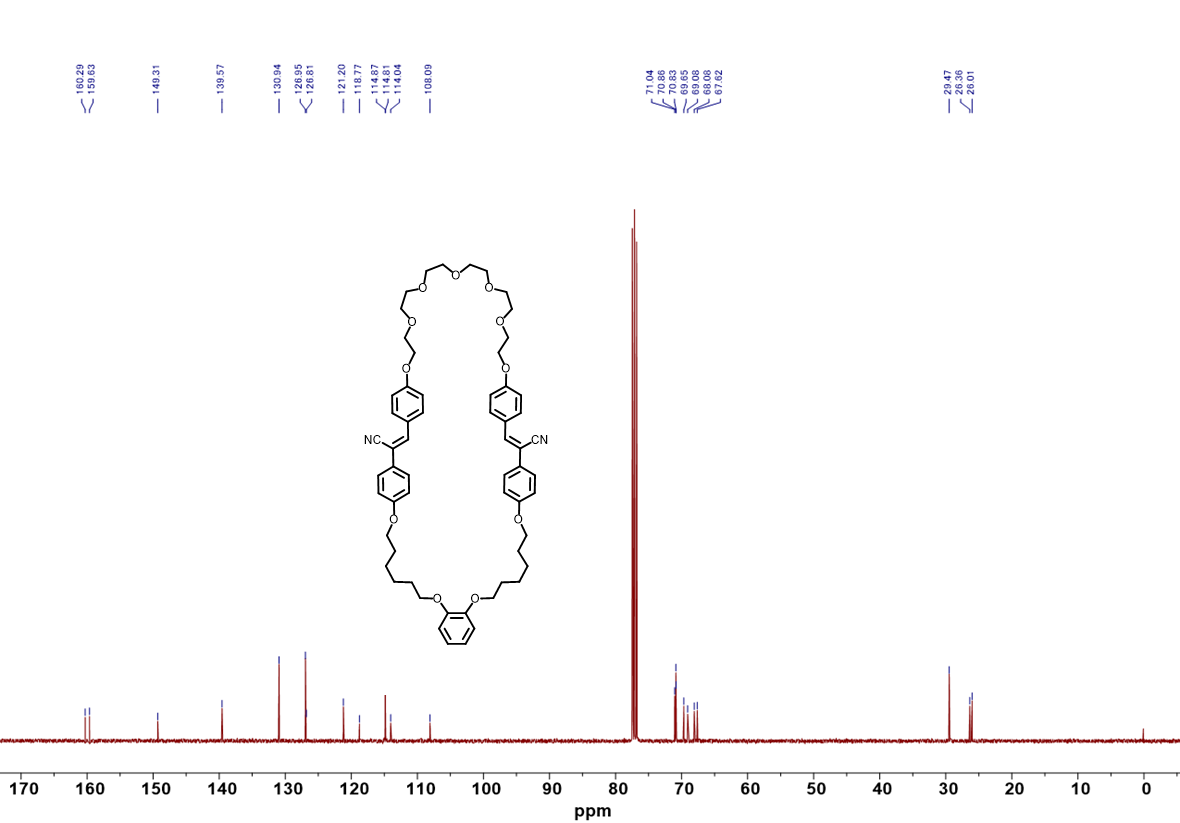


**Synthesis of MCH2**

**
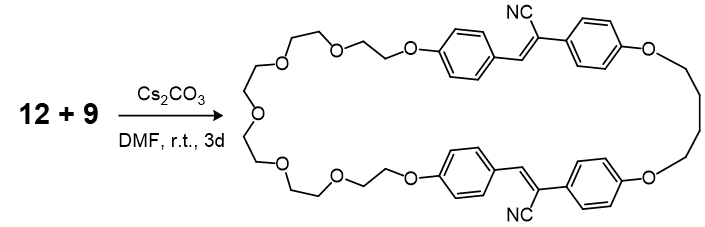
**

To a solution of **12** (0.386 g, 0.97 mmol) and **9** (0.7 g, 0.97 mmol) in DMF (80 mL) was added Cs_2_CO_3_ (1.89 g, 5.82 mmol). The mixture was stirred at room temperature for 3 days. Then the reaction mixture was poured into water and extracted with ethyl acetate. The organic phase was dried over MgSO_4_ and evaporated. The crude was purified via the silica column chromatography (CH_2_Cl_2_/ Ethyl acetate). **MCH2** was obtained as a yellow solid (Yield: 0.26 g, 35%). ^1^H NMR (500 MHz, CHLOROFORM-*D*) δ 7.70 (d, *J* = 8.7 Hz, 4H), 7.35 (d, *J* = 8.7 Hz, 4H), 7.19 (s, 2H), 6.84 (d, *J* = 8.7 Hz, 4H), 6.65 (d, *J* = 8.7 Hz, 4H), 4.23 – 4.06 (m, 8H), 3.88 (t, *J* = 9.5 Hz, 4H), 3.77 – 3.61 (m, 16H), 1.99 (br t, *J* = 8.3 Hz, 4H). ^13^C NMR (101 MHz, CHLOROFORM-*D*) δ 160.30, 158.93, 139.79, 130.97, 127.05, 126.80, 118.81, 115.14, 114.92, 108.05, 70.89, 70.86, 70.81, 69.67, 67.69, 67.47, 24.87. HR-MS (ESI): calcd for , [C_16_H_50_N_2_O_9_+Na]^+^ , m/z = 797.3414, found m/z = 797.3410.

^1^H NMR (500 MHz, CHCl₃-d, 298 K) spectrum of **MCH2**


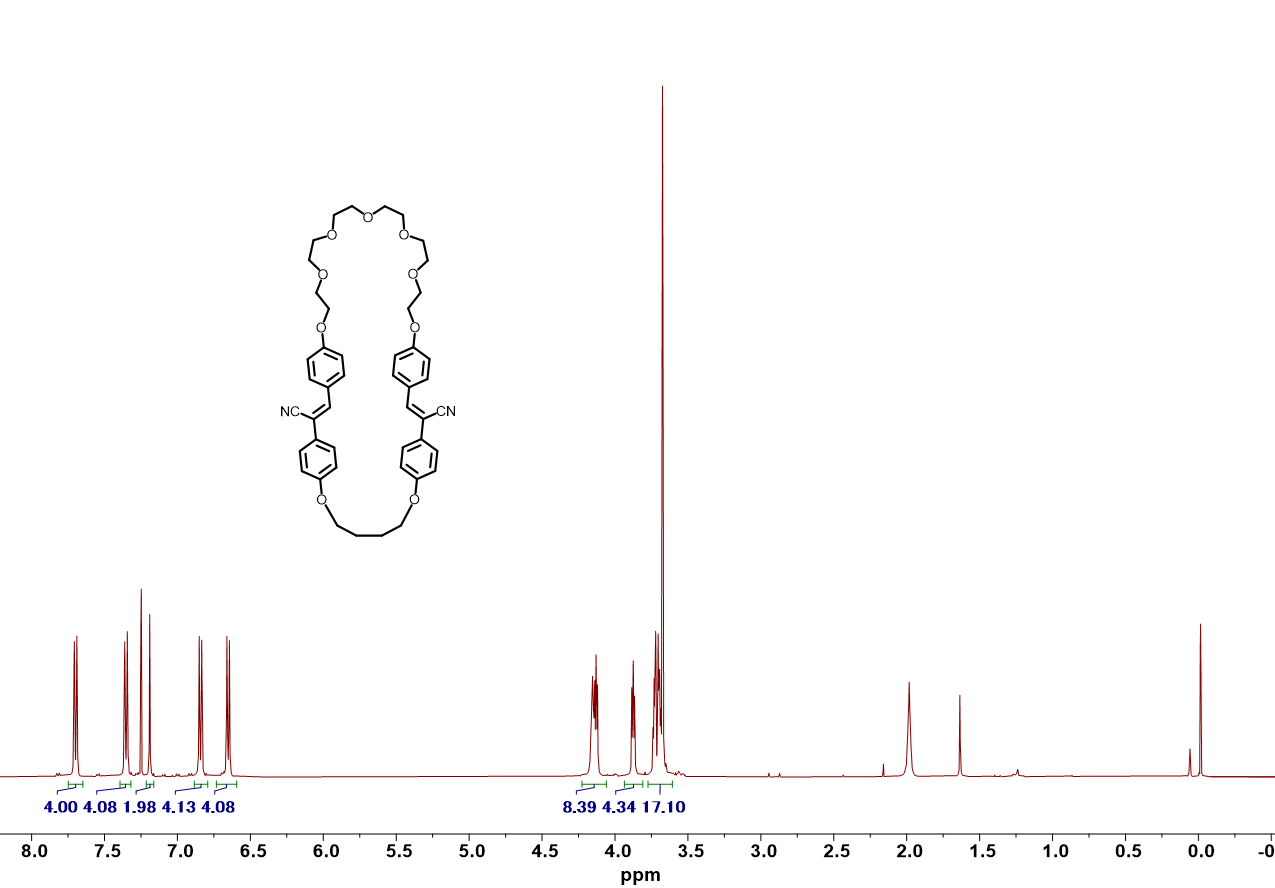


^13^C NMR (100 MHz, CHCl₃-d, 298 K) spectrum of **MCH2**


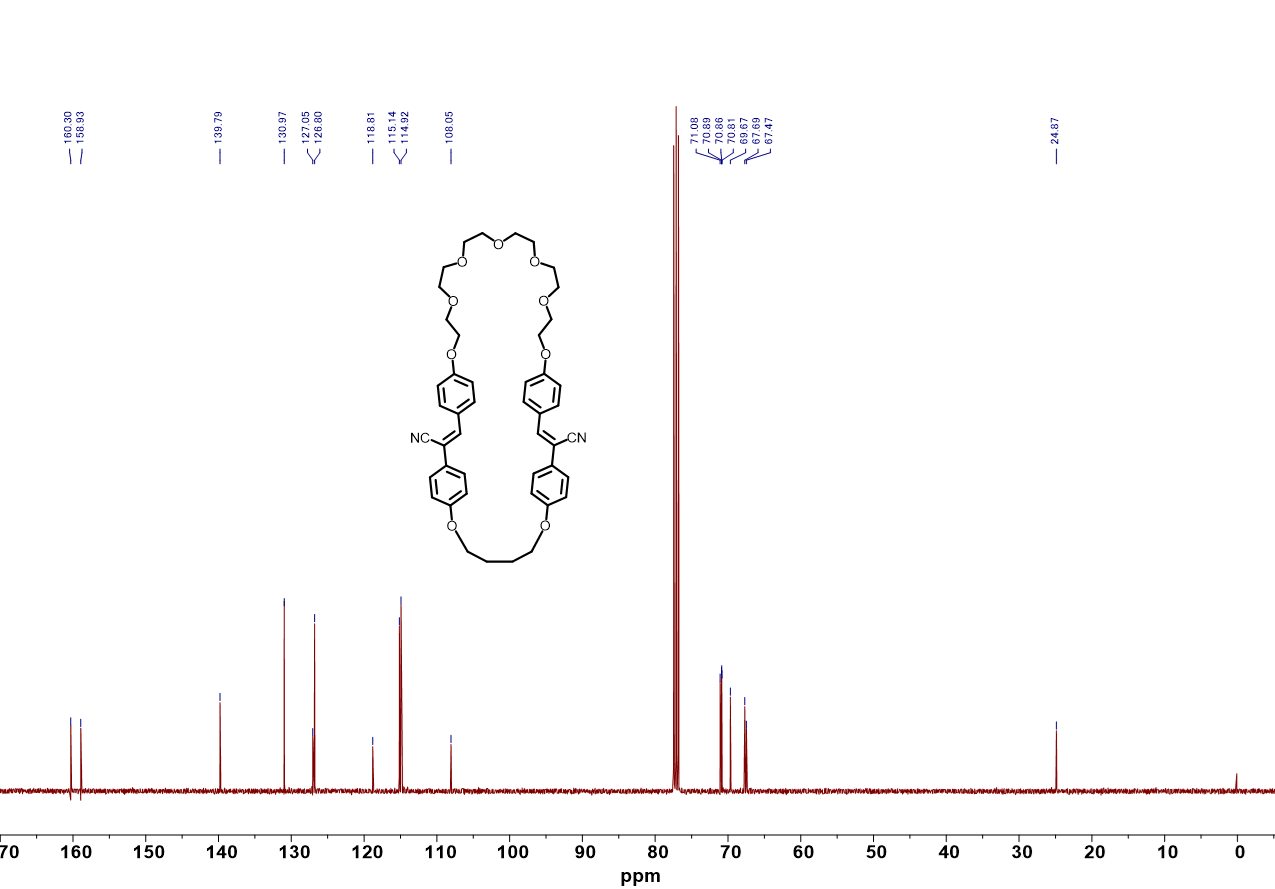


**Synthesis of MCH3**


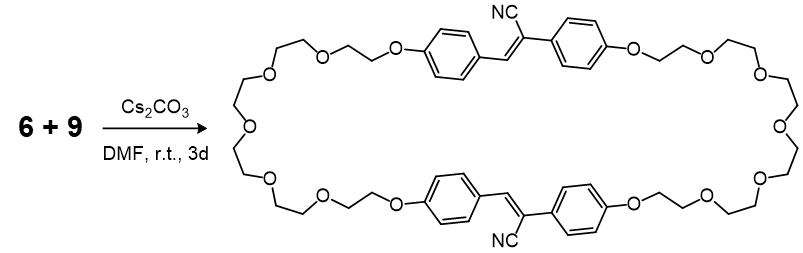


To a solution of **6** (0.573 g, 0.97 mmol) and **9** (0.7 g, 0.97 mmol) in DMF (80 mL) was added Cs_2_CO_3_ (1.89 g, 5.82 mmol). The mixture was stirred at room temperature for 3 days. Then the reaction mixture was poured into water and extracted with ethyl acetate. The organic phase was dried over MgSO_4_ and evaporated. The crude was purified via the silica column chromatography (CH_2_Cl_2_/ Ethyl acetate). **MCH3** was obtained as a yellow solid (Yield: 0.24 g, 26%). ^1^H NMR (400 MHz, CHLOROFORM-*D*) δ 7.72 (d, *J* = 8.8, 4H), 7.45 (d, *J* = 8.3, 4H), 7.19 (s, 2H), 6.88 (m, 8H), 4.12 – 4.04 (m, 8H), 3.83 (m, 8H), 3.74 – 3.59 (m, 32H). ^13^C NMR (101 MHz, CHLOROFORM-*D*) δ 160.35, 159.34, 139.84, 130.96, 127.39, 127.03, 126.82, 118.74, 115.04, 114.93, 108.11, 71.00, 70.79, 70.78, 69.70, 69.63, 67.66, 67.63. HR-MS (ESI): calcd for , [C_60_H_70_N_2_O_11_+Na]^+^ , m/z = 989.4412, found m/z = 989.4411.

^1^H NMR (400 MHz, CHCl₃-d, 298 K) spectrum of **MCH3**


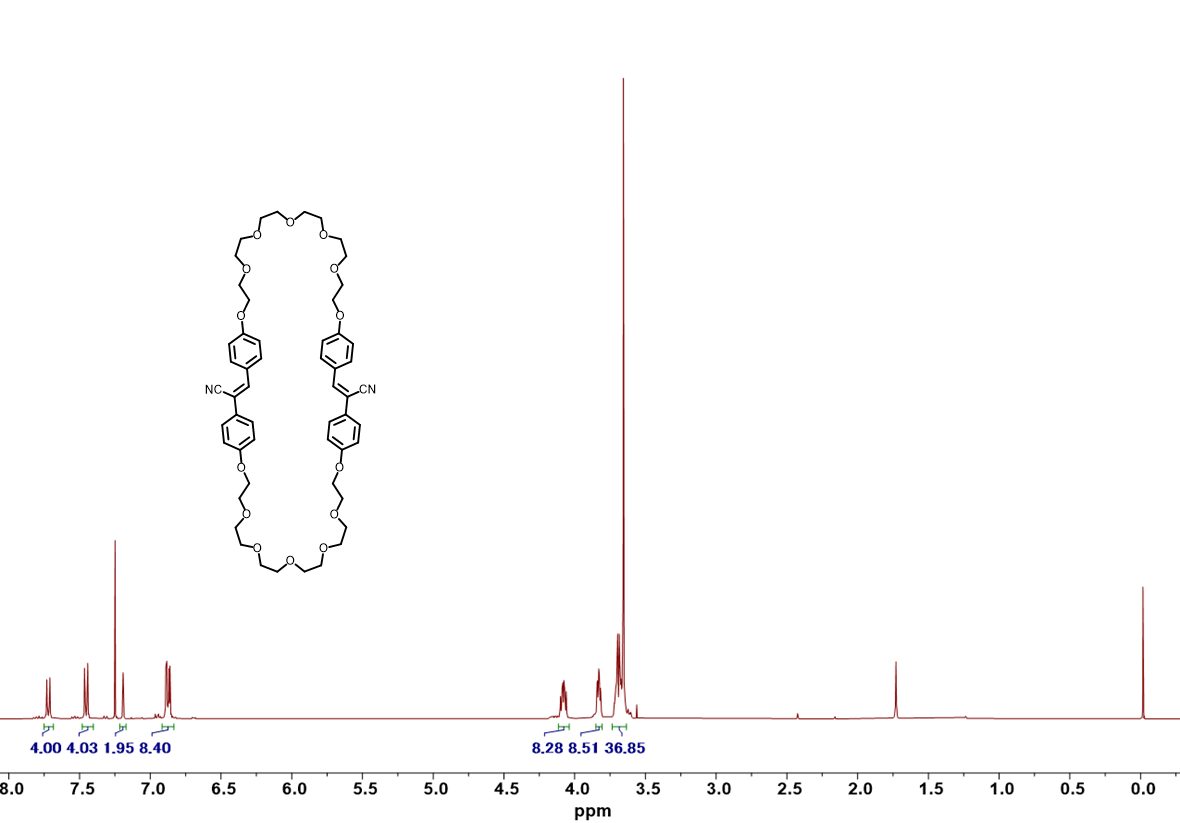


^13^C NMR (100 MHz, CHCl₃-d, 298 K) spectrum of **MCH3**


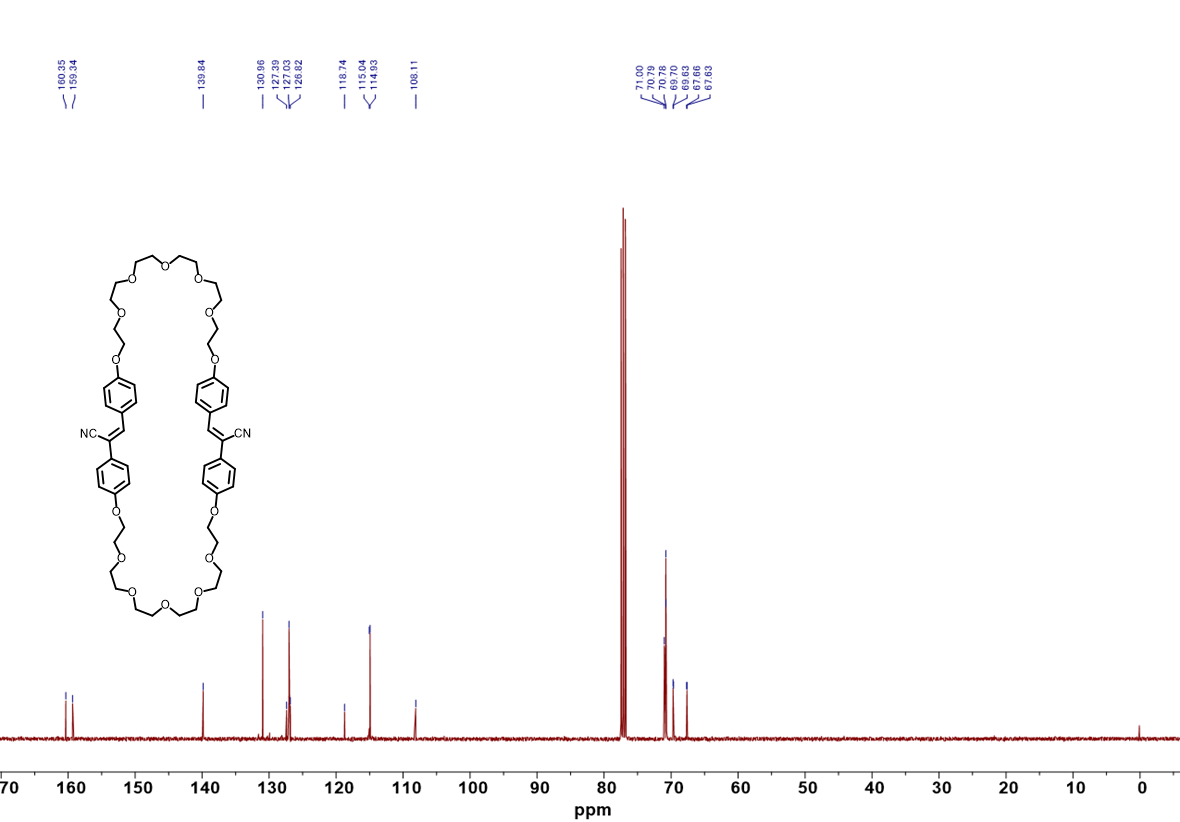


**Synthesis of cMCH1 and oMCH1 by intramolecular [2+2] photocycloaddition and growth of their single crystal**


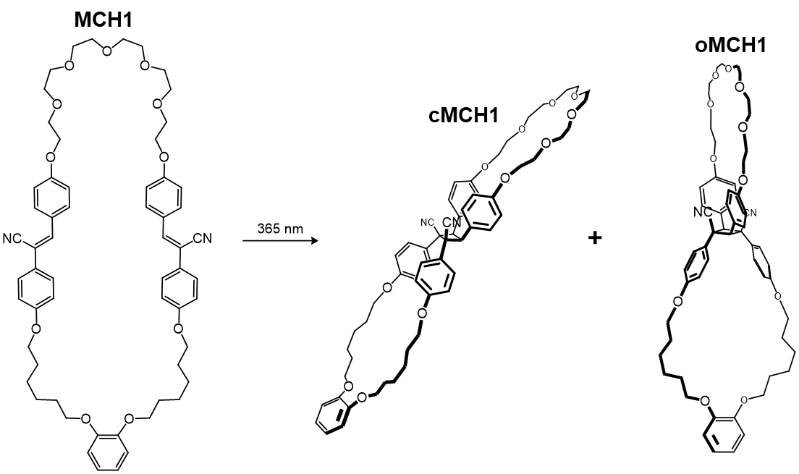


Synthesis: **MCH1** (100 mg, 25 mM) was dissolved in CHCl_3_ (4.2 mL) and degassed by bubbling with N₂. The solution was then cooled to -78 °C using a low temperature reactor and irradiated with 365 nm UV light overnight. After irradiation, the solution was directly subjected to silica column chromatography for isolation (CH_2_Cl_2_/Ethyl acetate = 100/0 – 60/40, gradient elution). **cMCH1** and **oMCH1** were isolated as white solid (**cMCH1**: 72 mg, 72%; **oMCH1**: 4.3 mg, 4.3%, Mixture: 19.1 mg, 19.1%; In total: 95%).

*Note: Partial overlap of* ***cMCH1*** *and* ***oMCH1*** *during column elution led to loss in yield of pure compounds.*

**cMCH1**: ^1^H NMR (500 MHz, CHLOROFORM-*D*) δ 7.15 (d, *J* = 8.8 Hz, 4H), 7.12 (d, *J* = 8.8 Hz, 4H), 6.90 (s, 4H), 6.82 (d, *J* = 8.8 Hz, 4H), 6.64 (d, *J* = 8.8 Hz, 4H), 4.91 (s, 2H), 4.16 – 4.06 (m, 4H), 4.00 (t, *J* = 6.1 Hz, 1H), 3.87 (t, *J* = 6.6 Hz, 1H), 3.82 (t, *J* = 4.7 Hz, 4H), 3.71 – 3.60 (m, 16H), 1.82 (p, *J* = 6.3 Hz, 4H), 1.71 (m, 4H), 1.54 (m, 4H), 1.46 (m, 4H). ^13^C NMR (101 MHz, CHLOROFORM-*D*) δ 159.29, 158.10, 149.43, 130.65, 128.84, 126.63, 125.54, 121.42, 119.48, 114.64, 114.62, 71.07, 70.91, 70.73, 69.71, 69.20, 68.06, 67.56, 54.92, 46.34, 29.40, 28.87, 25.87, 25.66. HR-MS (ESI): calcd for , [C_60_H_70_N_2_O_11_+Na]^+^ , m/z = 1017.4877, found m/z = 1017.4880.

**oMCH1**: ^1^H NMR (500 MHz, CHLOROFORM-*D*) δ 7.69 (d, *J* = 8.8 Hz, 4H), 7.23 – 7.20 (m, 8H), 6.87 – 6.83 (m, 8H), 4.37 (t, *J* = 3.5 Hz, 4H), 4.28 (s, 2H), 4.13 (t, *J* = 6.3 Hz, 4H), 3.94 (t, *J* = 7.3 Hz, 4H), 3.83 – 3.77 (t, *J* = 8.6 Hz, 4H), 3.70 (m, 4H), 3.64 – 3.51 (m, 12H), 1.78 (m, 4H), 1.71 (m, 4H), 1.43 (m, 8H). ^13^C NMR (126 MHz, CHLOROFORM-*D*) δ 159.80, 158.55, 148.66, 132.88, 130.20, 126.77, 126.43, 120.87, 119.34, 116.63, 115.86, 113.08, 71.11, 70.62, 70.49, 69.90, 69.09, 68.88, 67.70, 54.89, 50.53, 29.77, 29.11, 28.26, 25.68, 25.53. HR-MS (ESI): calcd for , [C_60_H_70_N_2_O_11_+Na]^+^ , m/z = 1017.4877, found m/z = 1017.4885.

Single crystal growth: single crystal of **cMCH1** and **oMCH1** were growth by the by vapor diffusion of hexane into their CHCl₃ solutions at 4 °C.

^1^H NMR (500 MHz, CHCl₃-d, 298 K) spectrum of **cMCH1**


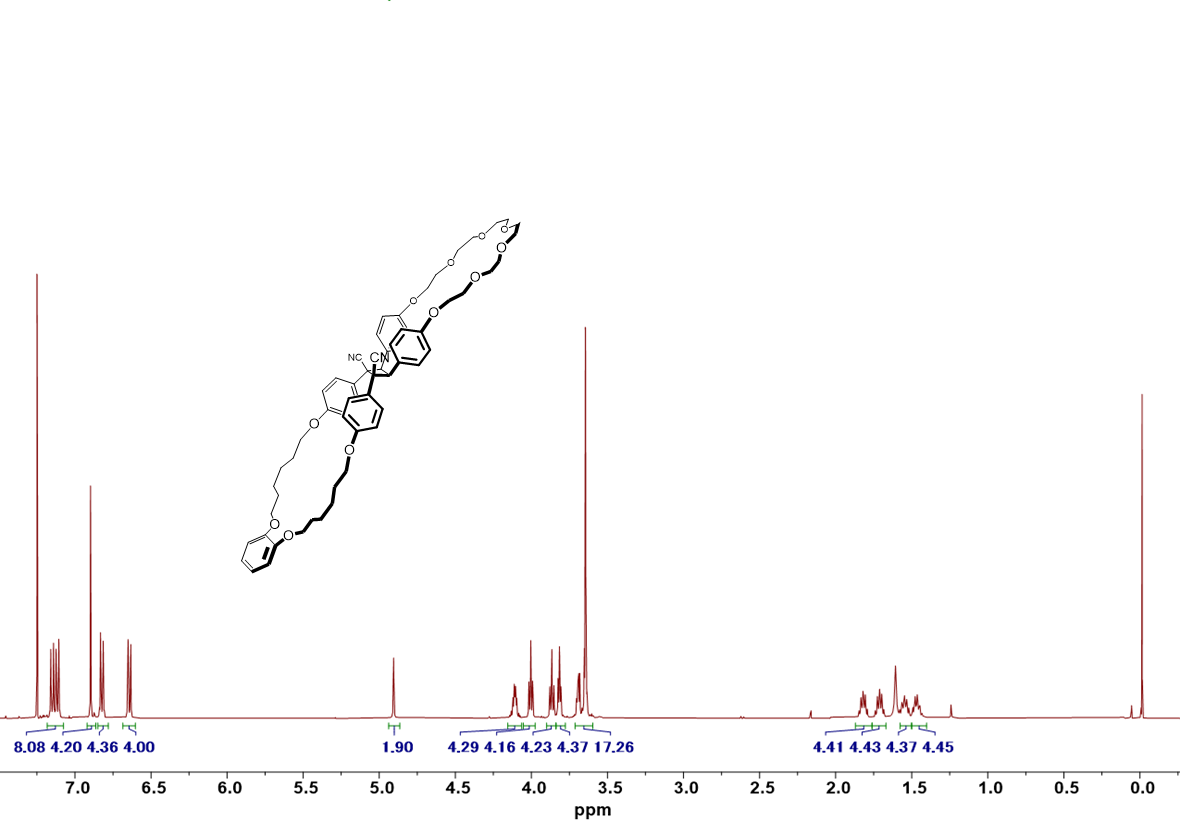


^13^C NMR (100 MHz, CHCl₃-d, 298 K) spectrum of **cMCH1**


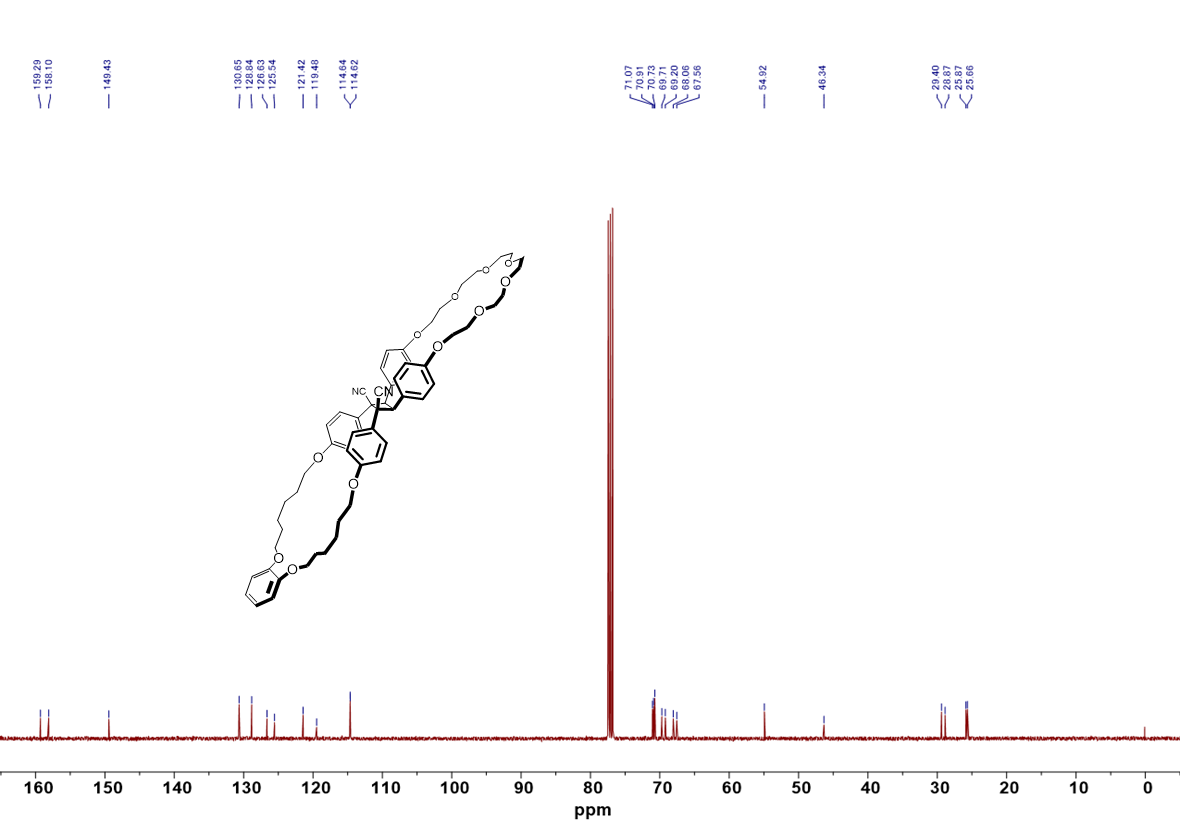


^1^H NMR (500 MHz, CHCl₃-d, 298 K) spectrum of **oMCH1**


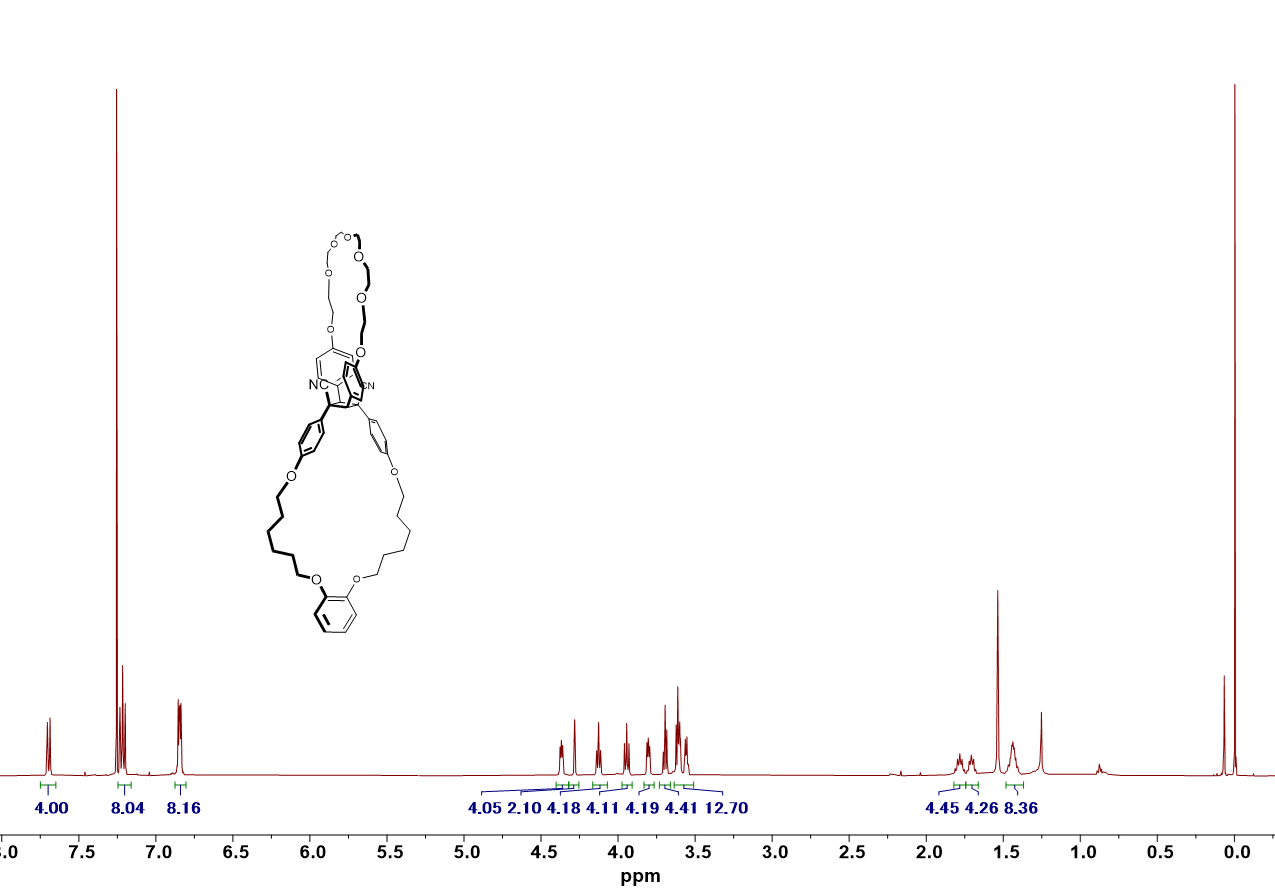


^13^C NMR (125 MHz, CHCl₃-d, 298 K) spectrum of **oMCH1**


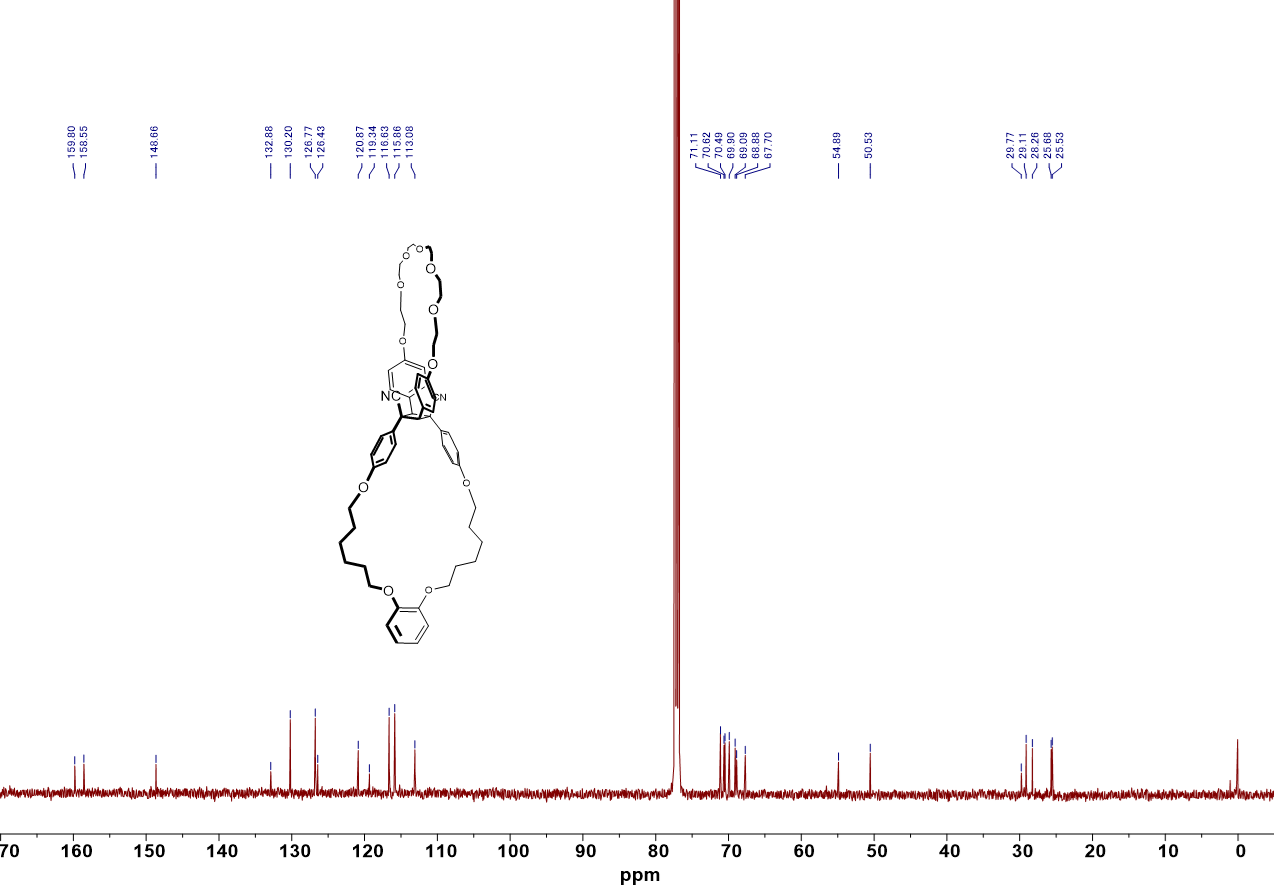


**Details of Single-Crystal X-ray Structure Determinations**

**cMCH1**: Single crystal X‑ray diffraction data were collected at 223.15 K on a Rigaku XtaLAB Synergy‑R four‑circle diffractometer using Mo Kα radiation (λ = 0.71073 Å) from a rotating‑anode X‑ray tube (Rigaku (Mo) X‑ray Source). Cell refinement, data collection, and reduction were performed with CrysAlisPro version 1.171.41.122a (Rigaku OD, 2021), and an empirical multi‑scan absorption correction was applied ^8^. Reflections were merged by SHELXL according to the crystal class to ensure robust statistical treatment for subsequent refinement. The structure was solved using SHELXT 2018/2 (Sheldrick, 2018) and refined by full‑matrix least‑squares on F² with SHELXL 2018/3 (Sheldrick, 2015), where hydrogen atoms were placed in idealized positions and constrained to ride on their parent atoms. Molecular graphics and publication materials were prepared using Olex2 version 1.5 ^9,10,11^. See Tables S1 for a summary of data collection, solution and refinement details. Complete details of the structures can be obtained from the Cambridge Crystallographic Data Centre at www.ccdc.cam.ac.uk for CCDC accession numbers 2443528.

**oMCH1**: Single crystal X‑ray diffraction data were collected at 120.15 K on a Rigaku XtaLAB Synergy‑R four‑circle diffractometer using Mo Kα radiation (λ = 0.71073 Å) from a rotating‑anode X‑ray tube (Rigaku (Mo) X‑ray Source). Data collection, cell refinement, and integration were performed with CrysAlisPro version 1.171.41.122a (Rigaku Oxford Diffraction, 2021)^8^. An empirical multi‑scan absorption correction was applied using spherical harmonics as implemented in the SCALE3 ABSPACK scaling algorithm. Reflection merging was carried out by SHELXL according to the crystal class, and structure factors included contributions from the .fab file. The structure was solved with SHELXT 2018/2 (Sheldrick, 2018) and refined by full‑matrix least‑squares on F² with SHELXL 2019/3 (Sheldrick, 2015), employing riding constraints for the hydrogen atoms. Molecular graphics and publication materials were prepared using Olex2 version 1.5 ^9,10,11^. See Tables S1 for a summary of data collection, solution and refinement details. Complete details of the structures can be obtained from the Cambridge Crystallographic Data Centre at www.ccdc.cam.ac.uk for CCDC accession numbers 2443529.

| Table S1 X-ray experimental details for **cMCH1** and **oMCH1**. | | |  |
| --- | --- | --- | --- |
| Compound | **cMCH1** | **oMCH1** | |
| CCDC No. | 2443528 | 2443529 | |
| Empirical formula | C_62_H_72_Cl_6_N_2_O_11_ | C_63_H_72_Cl_9_N_2_O_11_ | |
| Formula weight | 1233.91 | 1352.27 | |
| Temperature/K | 223.15 | 120.15 | |
| Crystal system | triclinic | triclinic | |
| Space group | P-1 | P-1 | |
| a/Å | 12.8790(3) | 10.4396(3) | |
| b/Å | 13.7920(3) | 13.9469(4) | |
| c/Å | 18.8490(4) | 23.7674(6) | |
| α/° | 85.565(2) | 78.125(2) | |
| β/° | 77.924(2) | 78.429(2) | |
| γ/° | 73.013(2) | 79.111(2) | |
| Volume/Å^3^ | 3130.69(13) | 3278.08(16) | |
| Z | 2 | 2 | |
| ρ_calc_g/cm^3^ | 1.309 | 1.370 | |
| μ/mm^‑1^ | 0.334 | 0.443 | |
| F(000) | 1296.0 | 1410.0 | |
| Crystal size/mm^3^ | 0.4 × 0.25 × 0.1 | 0.3 × 0.04 × 0.03 | |
| Radiation | Mo Kα (λ = 0.71073) | Mo Kα (λ = 0.71073) | |
| 2Θ range for data collection/° | 3.088 to 62.364 | 3.02 to 59.842 | |
| Reflections collected | 72299 | 32513 | |
| Independent reflections | 16551 | 14894 | |
| Data/restraints/parameters | 16552/2575/1315 | 14894/4/768 | |
| Goodness-of-fit on F^2^ | 1.034 | 1.047 | |
| *R*_1_ [I>=2σ (I)] | 0.0972 | 0.0820 | |
| *R*_1_ (all data) | 0.1360 | 0.1137 | |
| *wR*_2_ [I>=2σ (I)] | 0.2876 | 0.1957 | |
| *wR*_2_ (all data) | 0.3202 | 0.2127 | |
| Largest diff. peak/hole / e Å^-3^ | 0.74/-0.48 | 1.18/-0.63 | |

**Synthesis of cMCH2 by intramolecular [2+2] photocycloaddition**


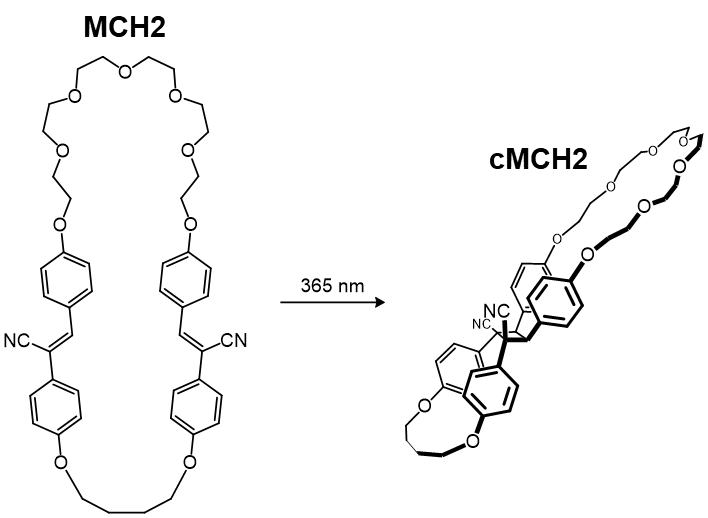


**MCH2** (78 mg, 25 mM) was dissolved in CHCl_3_ (4.2 mL) and degassed by bubbling with N₂. The solution was then cooled to -78 °C using a low temperature reactor and irradiated with 365 nm UV light overnight. After irradiation, the solvent was removed under reduced pressure to give **cMCH2** as a white solid quantitatively. ^1^H NMR (500 MHz, CHLOROFORM-*D*) δ 7.21 (dd, *J* = 8.6, 2.6 Hz, 2H), 7.18 – 7.12 (d, 4H), 7.00 (dd, *J* = 8.7, 2.6 Hz, 2H), 6.88 – 6.79 (d, 4H), 6.62 (m, *J* = 15.8, 8.6, 2.7 Hz, 4H), 4.93 (s, 2H), 4.23 (t, 4H), 4.15 – 4.08 (m, 4H), 3.82 (t, *J* = 4.7 Hz, 4H), 3.72 – 3.60 (m, 16H), 1.58 (m, 4H). ^13^C NMR (126 MHz, CHLOROFORM-*D*) δ 158.18, 156.87, 131.13, 130.64, 126.75, 126.53, 125.67, 119.12, 117.33, 116.56, 114.71, 71.08, 70.93, 70.75, 69.73, 67.62, 66.19, 55.73, 44.89, 21.31. HR-MS (ESI): calcd for , [C_16_H_50_N_2_O_9_+Na]^+^ , m/z = 797.3414, found m/z = 797.3420.

^1^H NMR (500 MHz, CHCl₃-d, 298 K) spectrum of **cMCH2**

*
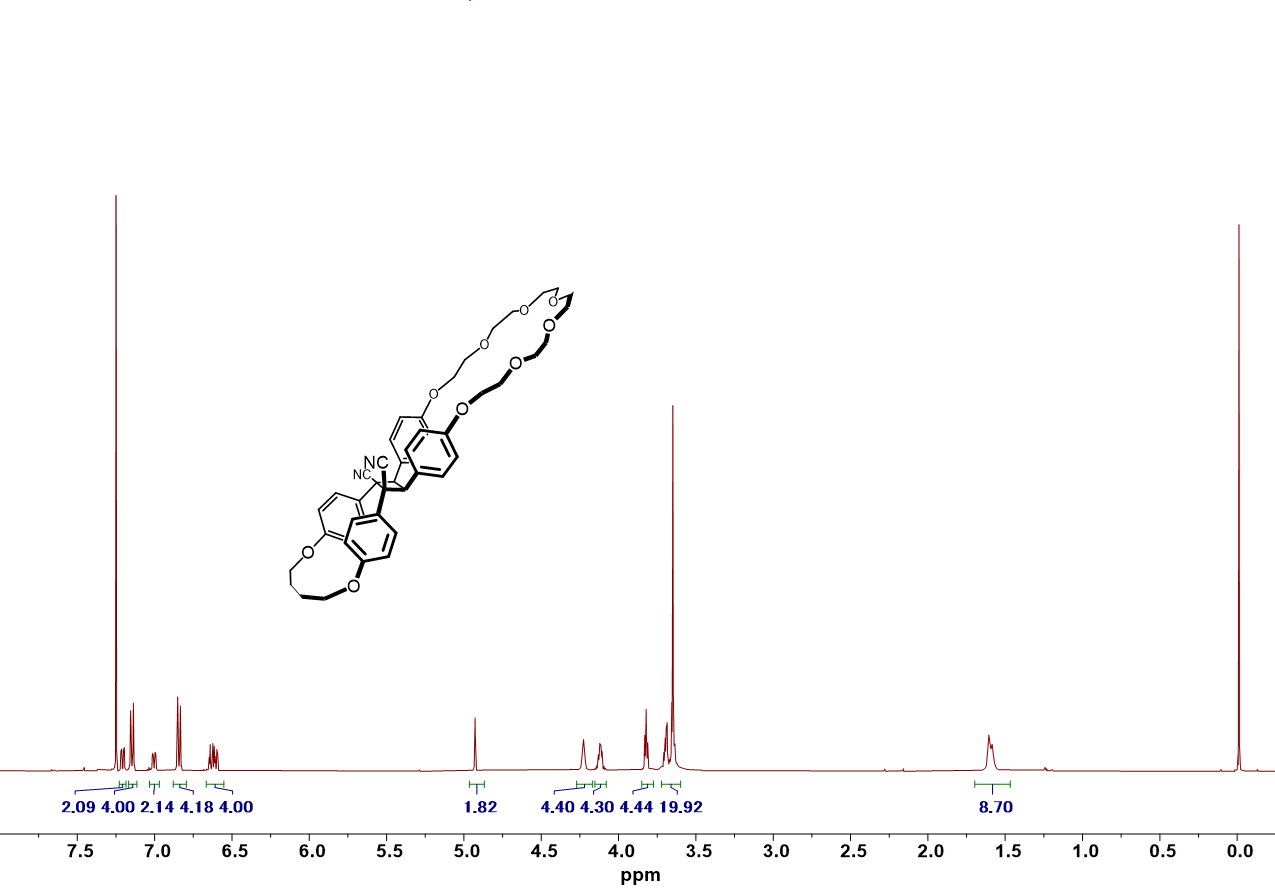
*

^13^C NMR (125 MHz, CHCl₃-d, 298 K) spectrum of **cMCH2**


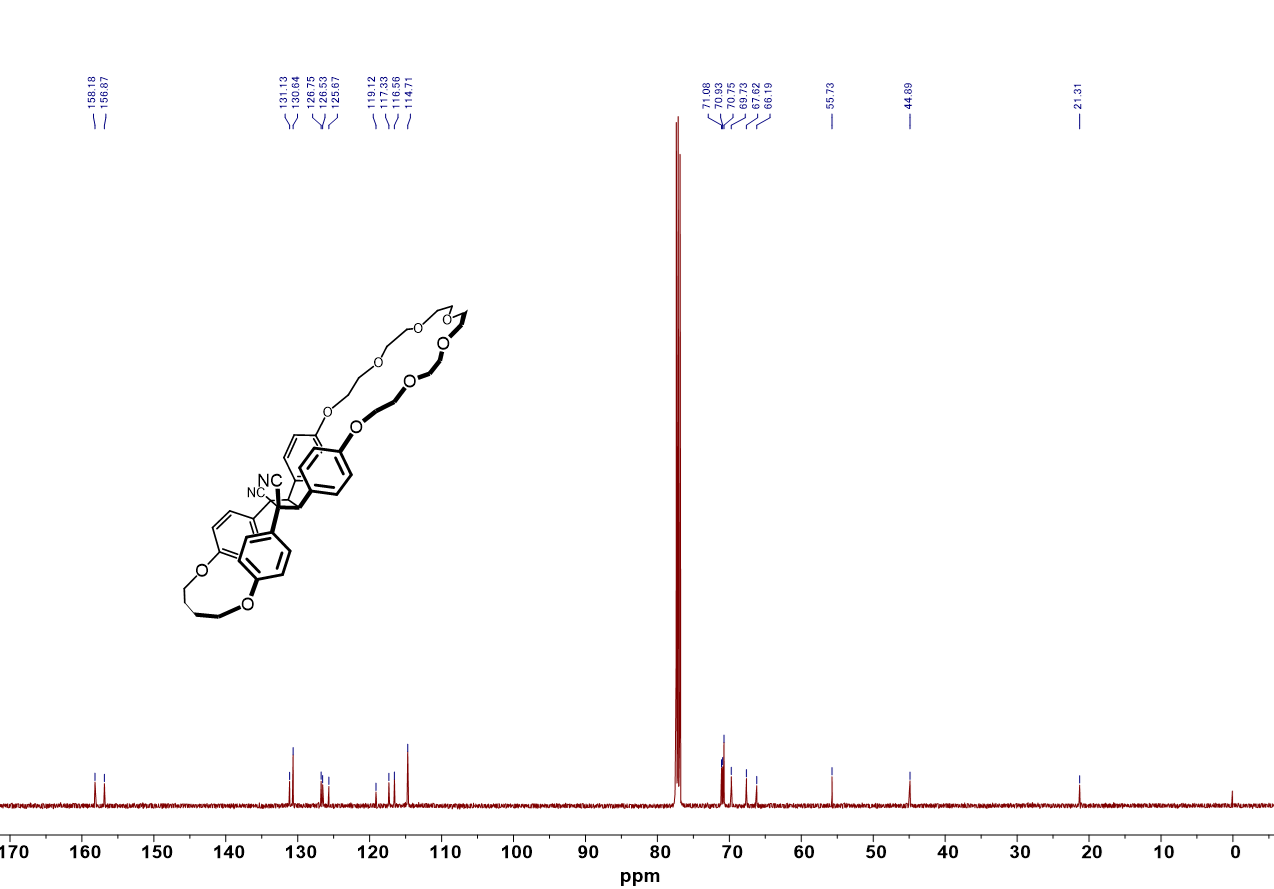


**Synthesis of cMCH3 and oMCH3 by intramolecular [2+2] photocycloaddition**


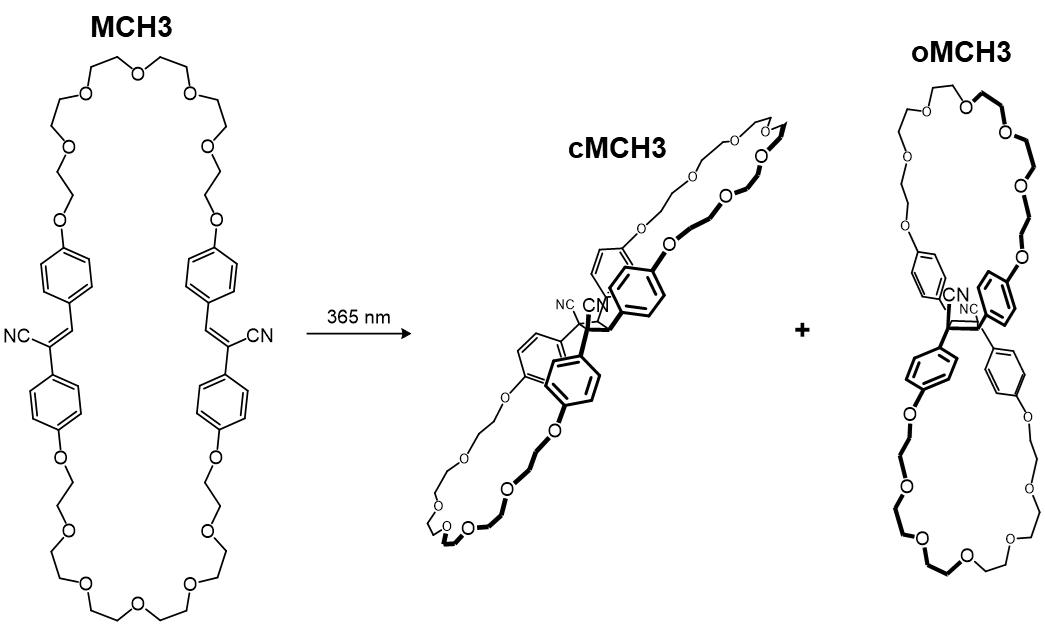


**MCH3** (97 mg, 25 mM) was dissolved in CHCl_3_ (4.2 mL) and degassed by bubbling with N₂. The solution was then cooled to -78 °C using a low temperature reactor and irradiated with 365 nm UV light for 2 days. After irradiation, the solution was directly subjected to silica column chromatography for isolation (Ethyl acetate/MeOH = 100/0 – 92/8, gradient elution). **cMCH3** and **oMCH3** were isolated as white solid (**cMCH3**: 70 mg, 72%; **oMCH3**: 4.3 mg, 4.4%; Mixture: 11.6 mg, 12%; In total: 88%).

*Note: Partial overlap of* ***cMCH3*** *and* ***oMCH3*** *during column elution led to loss in yield of pure compounds.*

**cMCH3**: ^1^H NMR (400 MHz, CHLOROFORM-D) δ 7.21 (d, *J* = 8.9 Hz, 4H), 7.10 (d, *J* = 8.9 Hz, 4H), 6.81 (d, *J* = 8.9 Hz, 4H), 6.72 (d, *J* = 8.9 Hz, 4H), 4.90 (s, 2H), 4.15 – 4.07 (m, 4H), 4.07 – 4.00 (m, 4H), 3.80 (m, 8H), 3.72 – 3.57 (m, 32H). ^13^C NMR (101 MHz, CHLOROFORM-*D*) δ 158.99, 158.10, 130.65, 128.80, 126.59, 126.03, 119.54, 114.86, 114.60, 71.10, 71.06, 70.93, 70.90, 70.88, 70.86, 70.73, 69.71, 69.64, 67.55, 54.60, 47.08. HR-MS (ESI): calcd for , [C_60_H_70_N_2_O_11_+Na]^+^ , m/z = 989.4412, found m/z = 989.4412.

**oMCH3**: ^1^H NMR (500 MHz, CHLOROFORM-*D*) δ 7.56 (d, *J* = 8.9 Hz, 4H), 7.29 (d, *J* = 8.9 Hz, 4H), 7.17 (d, *J* = 8.9 Hz, 4H), 7.98 (d, *J* = 8.9 Hz, 4H), 4.37 – 4.31 (m, 4H), 4.25 (s, 2H), 4.24 – 4.19 (m, 4H), 3.83 – 3.75 (m, 8H), 3.67 – 3.41 (m, 32H). ^13^C NMR (126 MHz, CHLOROFORM-*D*) δ 159.64, 159.17, 132.56, 129.74, 126.65, 126.35, 119.14, 116.51, 116.36, 71.10, 71.00, 70.74, 70.59, 70.57, 70.53, 70.46, 70.02, 69.84, 68.75, 68.26, 55.81. HR-MS (ESI): calcd for , [C_60_H_70_N_2_O_11_+Na]^+^ , m/z = 989.4412, found m/z = 989.4416.

^1^H NMR (400 MHz, CHCl₃-d, 298 K) spectrum of **cMCH3**


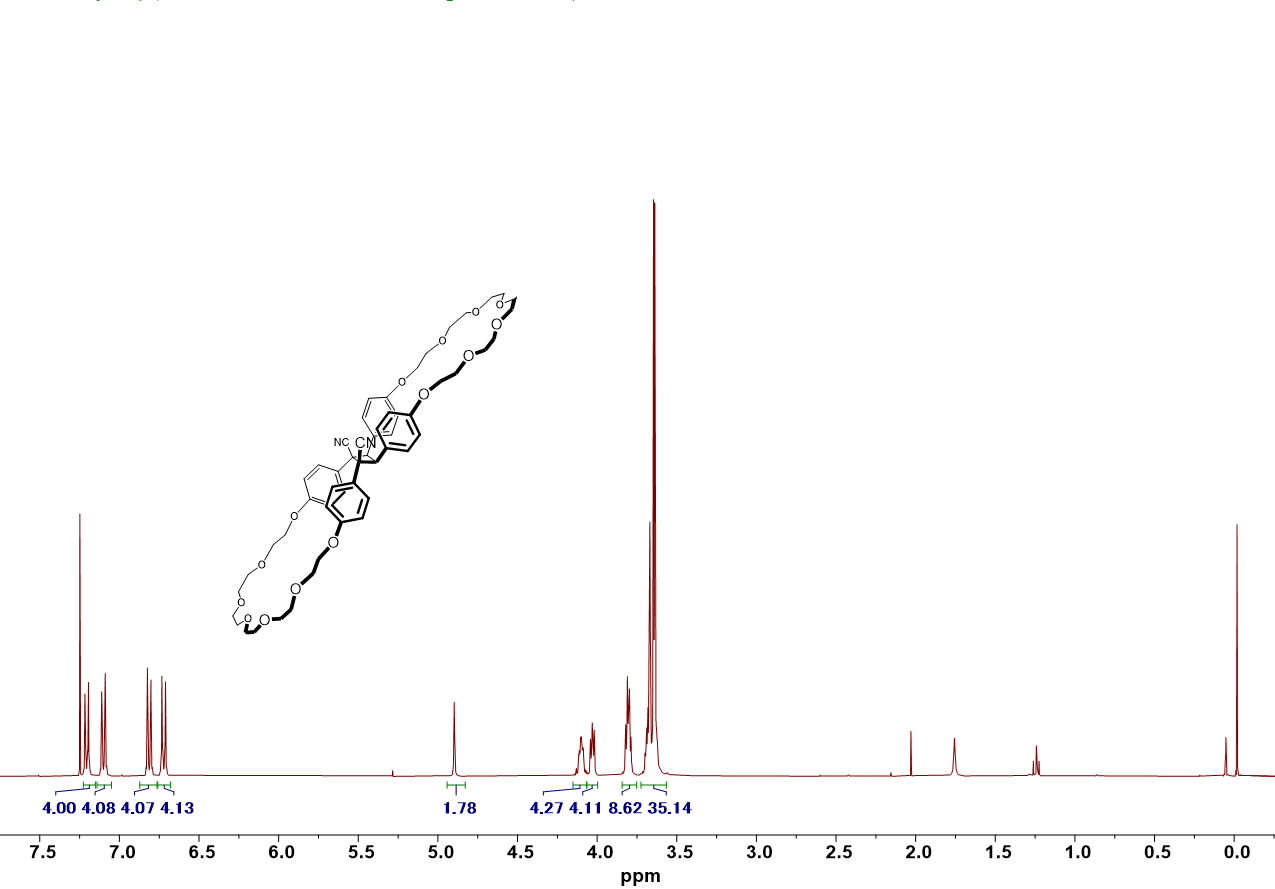


^13^C NMR (100 MHz, CHCl₃-d, 298 K) spectrum of **cMCH3**


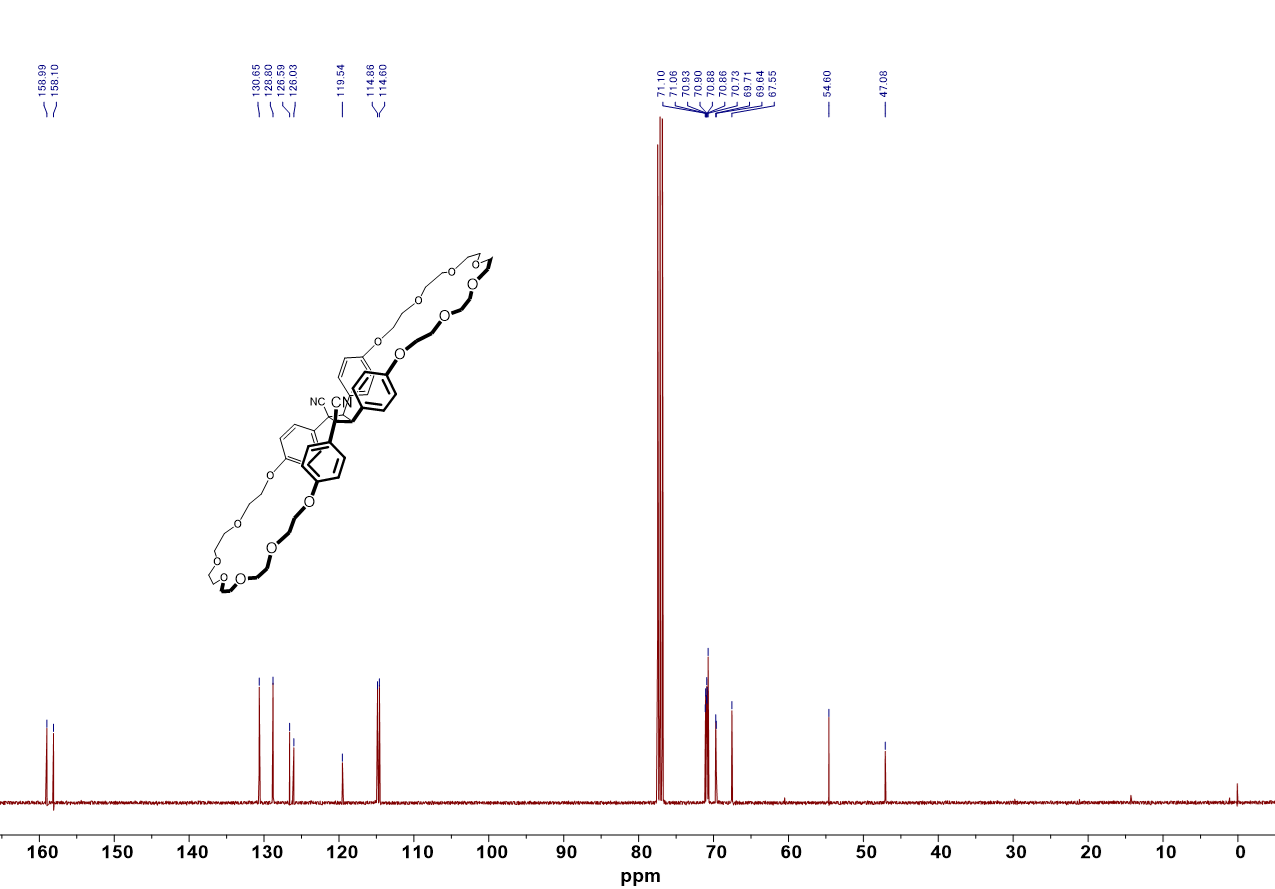


^1^H NMR (500 MHz, CHCl₃-d, 298 K) spectrum of **oMCH3**


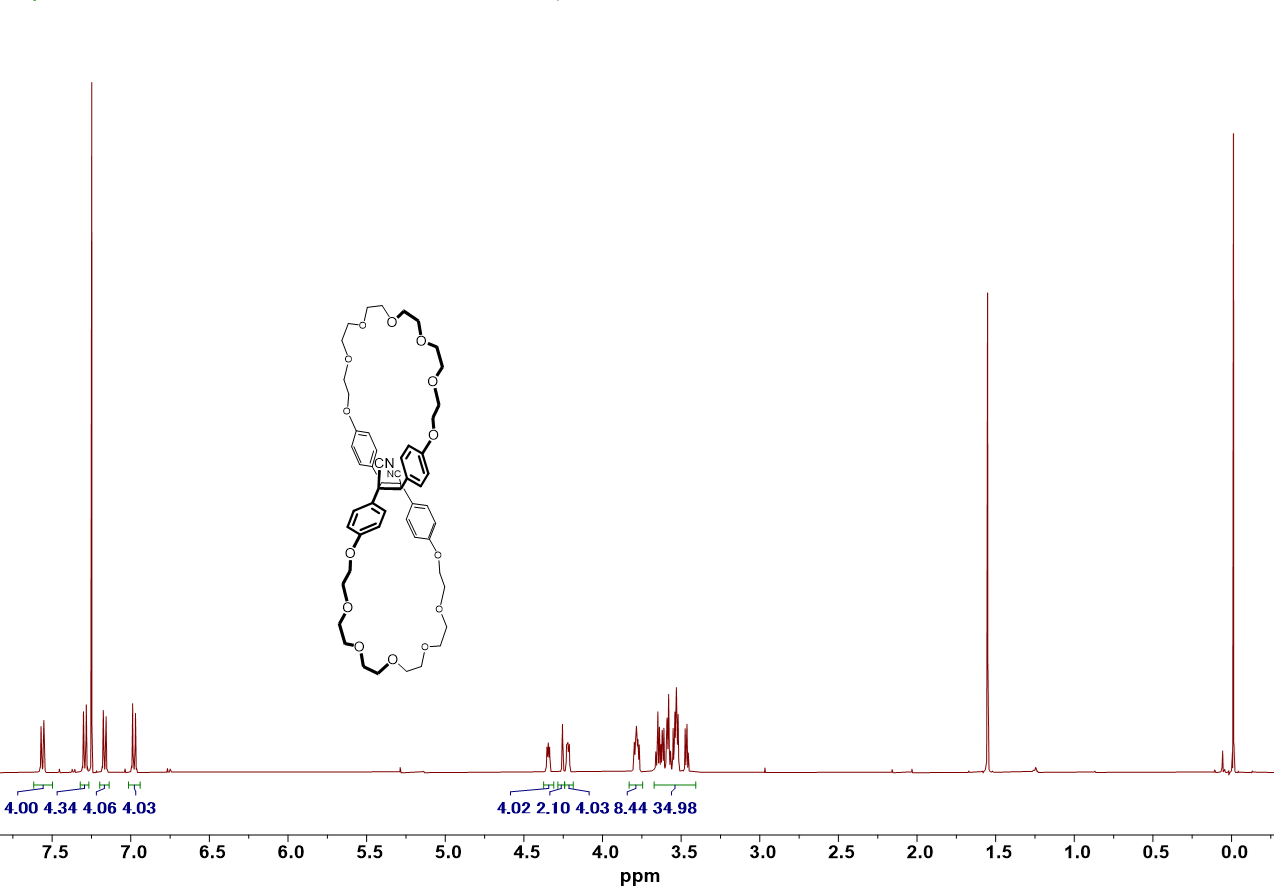


^13^C NMR (125 MHz, CHCl₃-d, 298 K) spectrum of **oMCH3**


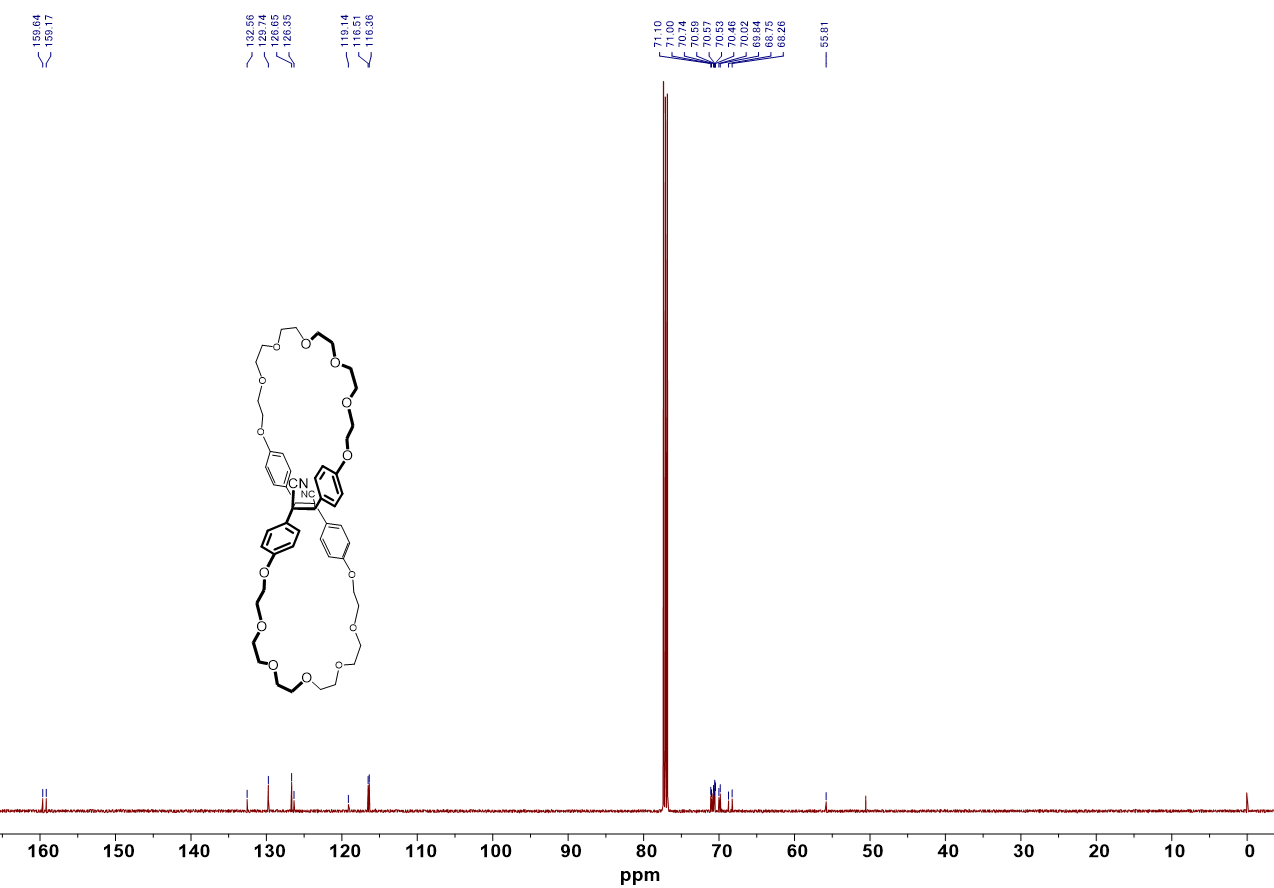


**Synthesis of c[2]RT(cMCH1@G) and of o[2]RT(oMCH1@G)**


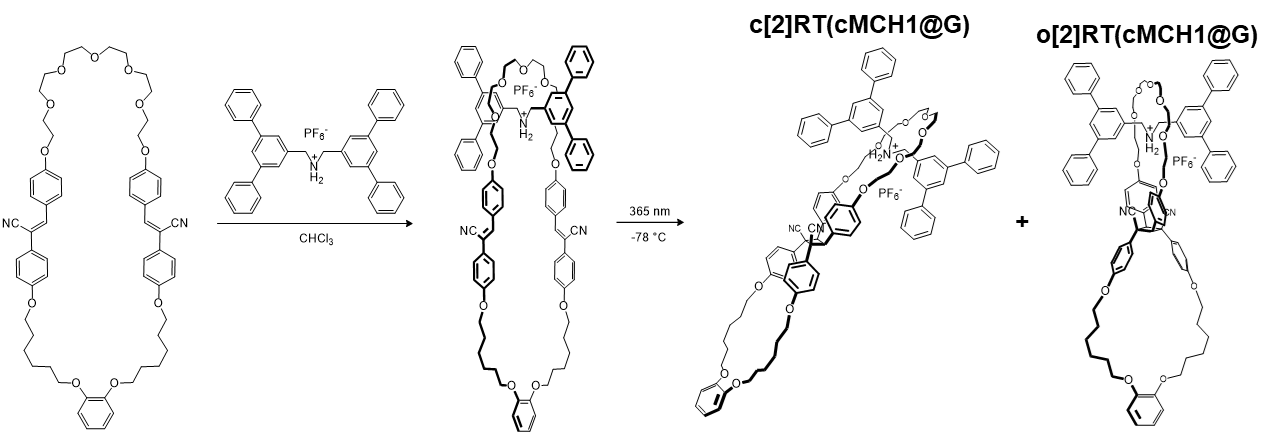


**MCH1** (100 mg, 0.1 mmol, 50 mM) and **G** (195 mg, 0.3 mmol) were dissolved in acetone (2 mL) and degassed by bubbling with N₂. The solution was then cooled to -78 °C using a low temperature reactor and irradiated with 365 nm UV light overnight. After irradiation, the solution was removed under reduced pressure without heating directly. The resulting residue was purified by silica column chromatography (CH_2_Cl_2_/Ethyl acetate = 100/0 – 88/12, gradient elution). **c[2]RT(cMCH1@G)** and **o[2]RT(oMCH1@G)** were isolated as white solid (**c[2]RT(cMCH1@G)**: 134 mg, 79%; **o[2]RT(oMCH1@G)**: 10.1 mg, 6.1%; Mixture: 11.2 mg, 6.8%; In total: 92%).

*Note: Partial overlap of* ***c[2]RT(cMCH1@G)*** *and* ***o[2]RT(oMCH1@G)*** *during column elution led to little loss in yield of pure compounds.*

**c[2]RT(cMCH1@G)**: ^1^H NMR (600 MHz, ACETONE-*D*_6_) δ 8.22 (br s, 2H), 8.11 (br t, *J* = 1.7 Hz, 1H), 8.08 (br t, *J* = 1.7 Hz, 1H), 7.94 (d, *J* = 1.7 Hz, 2H), 7.87 (d, *J* = 1.7 Hz, 2H), 7.86 – 7.83 (m, 8H), 7.56 (m, 8H), 7.49 – 7.42 (m, 4H), 7.31 (d, *J* = 8.8 Hz, 4H), 7.13 (d, *J* = 8.8 Hz, 4H), 6.96 (dd, *J* = 5.9, 3.6 Hz, 2H), 6.88 (dd, *J* = 6.0, 3.6 Hz, 2H), 6.78 (d, *J* = 8.8 Hz, 4H), 6.66 (d, *J* = 8.8 Hz, 4H), 5.17 (s, 2H), 4.78 (t, *J* = 6.3, 3.3 Hz, 2H), 4.68 – 4.63 (t, 2H), 4.01 (m, 8H), 3.97 (t, *J* = 6.6 Hz, 4H), 3.79 (m, 2H), 3.73 (m, 2H), 3.69 – 3.64 (m, 4H), 3.63 – 3.59 (m, 2H), 3.52 (m, 2H), 3.46 (m, 4H), 3.37 (m, 4H), 1.83 – 1.78 (m, 4H), 1.73 (m, 4H), 1.62 – 1.56 (m, 4H), 1.51 (m, 4H). ^13^C NMR (101 MHz, ACETONE-*D*_6_) δ 159.47, 157.61, 149.78, 142.50, 142.29, 140.02, 139.90, 132.56, 131.21, 130.97, 129.23, 129.08, 128.91, 128.19, 128.12, 127.93, 127.86, 127.46, 127.21, 127.15, 126.89, 126.82, 126.57, 125.77, 121.32, 119.76, 114.92, 114.67, 113.67, 70.97, 70.44, 70.18, 70.05, 69.93, 68.93, 67.85, 67.23, 59.72, 54.59, 46.16, 25.74, 25.47, 20.01, 13.68. HR-MS (ESI): calcd for [M – PF_6_]^+^ , [C_98_H_102_N_3_O_11_]^+^ , m/z = 1497.7543, found m/z = 1496.7538.

**o[2]RT(oMCH1@G)**: ^1^H NMR (600 MHz, ACETONE-*D*_6_) δ 8.05 (br t, *J* = 1.7 Hz, 2H), 7.86 – 7.83 (m, 8H), 7.81 (d, *J* = 1.7 Hz, 4H), 7.72 (d, *J* = 8.9 Hz, 4H), 7.53 – 7.55 (m, 8H), 7.47 – 7.41 (m, 8H), 7.13 – 7.07 (m, 8H), 6.90 (dd, *J* = 6.0, 3.6 Hz, 2H), 6.79 (dd, *J* = 6.0, 3.5 Hz, 2H), 4.84 (s, 2H), 4.43 – 4.37 (m, 4H), 4.26 (t, *J* = 7.0 Hz, 4H), 4.06 (m, 4H), 3.93 (t, *J* = 7.2 Hz, 4H), 3.87 – 3.81 (br, 4H), 3.54 – 3.51 (br, 4H), 3.48 – 3.43 (m, 4H), 3.24 – 3.27 (m, 4H), 2.97 – 2.91 (m, 4H), 1.78 (m, 8H), 1.50 (m, 8H). ^13^C NMR (101 MHz, ACETONE-*D*_6_) δ 205.35, 159.13, 158.65, 148.93, 142.18, 140.06, 132.46, 130.31, 129.21, 128.91, 128.43, 128.11, 128.07, 128.03, 127.54, 127.15, 126.53, 120.67, 120.34, 116.13, 116.01, 113.13, 71.09, 70.23, 70.16, 69.91, 68.94, 68.58, 67.63, 67.57, 55.70, 52.37, 50.63, 27.80, 25.13. HR-MS (ESI): calcd for [M – PF_6_]^+^ , [C_98_H_102_N_3_O_11_]^+^ , m/z = 1497.7543, found m/z = 1497.7546.

^1^H NMR (600 MHz, acetone-d6, 298 K) spectrum of **c[2]RT(cMCH1@G)**


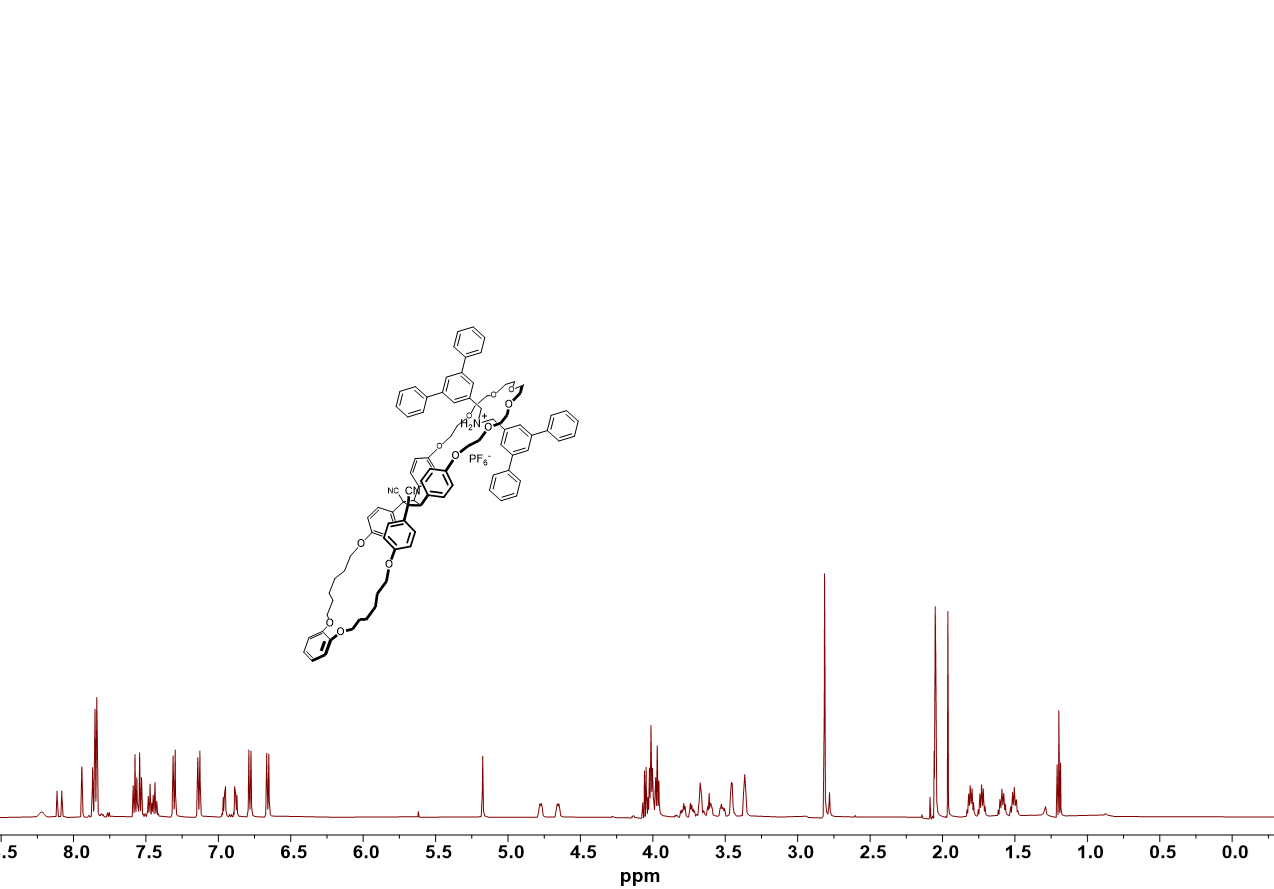


^13^C NMR (100 MHz, acetone-d6, 298 K) spectrum of **c[2]RT(cMCH1@G)**


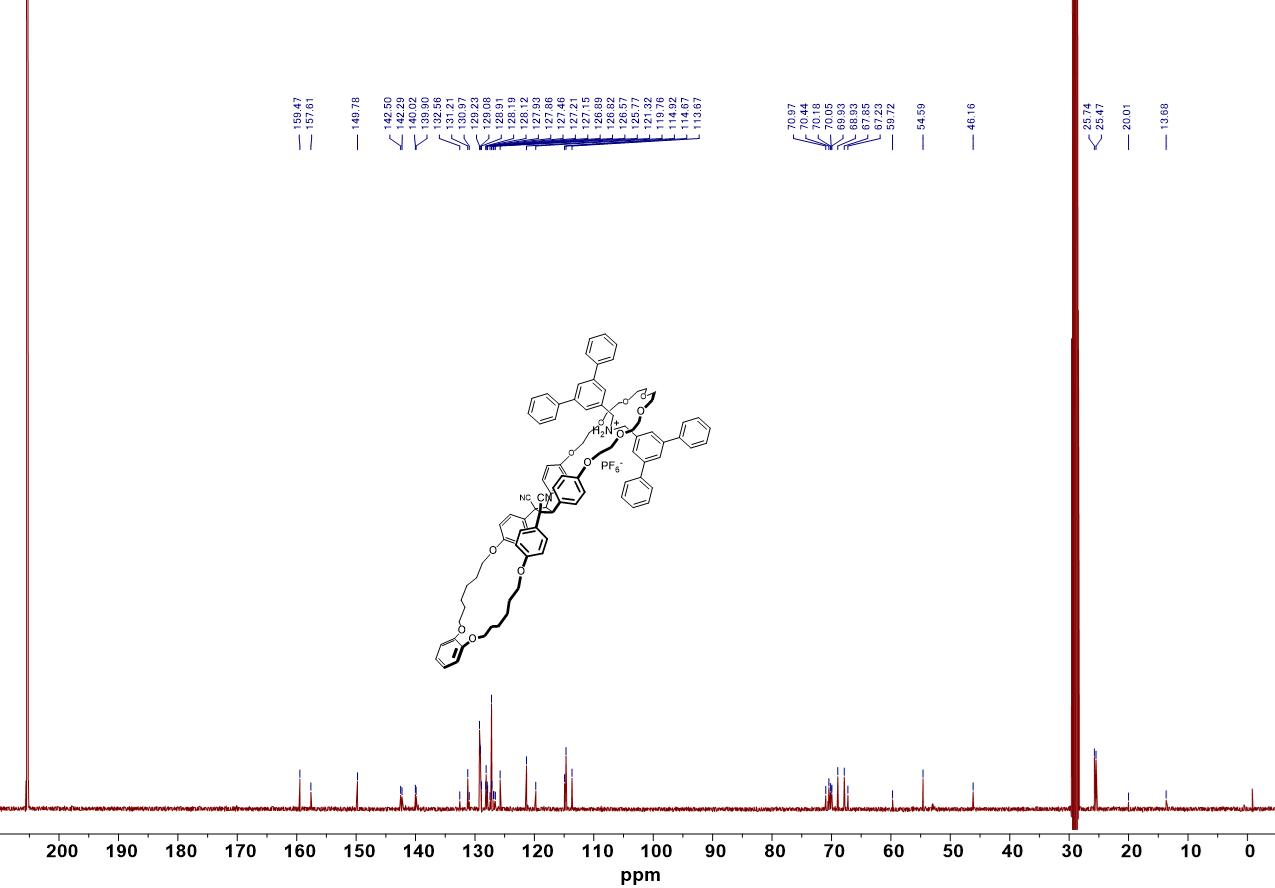


^1^H NMR (600 MHz, acetone-d6, 298 K) spectrum of **o[2]RT(oMCH1@G)**


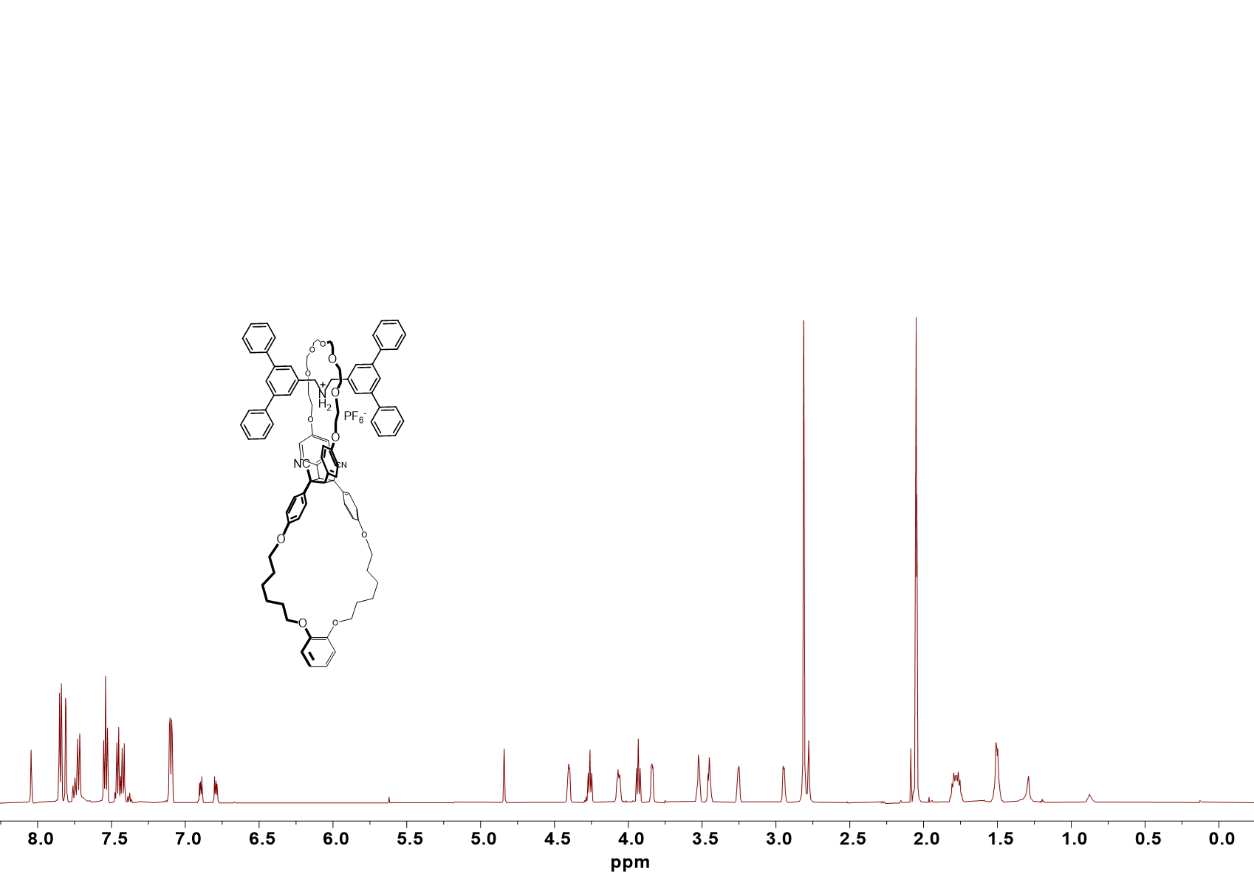


^13^C NMR (101 MHz, acetone-d6, 298 K) spectrum of **o[2]RT(oMCH1@G)**


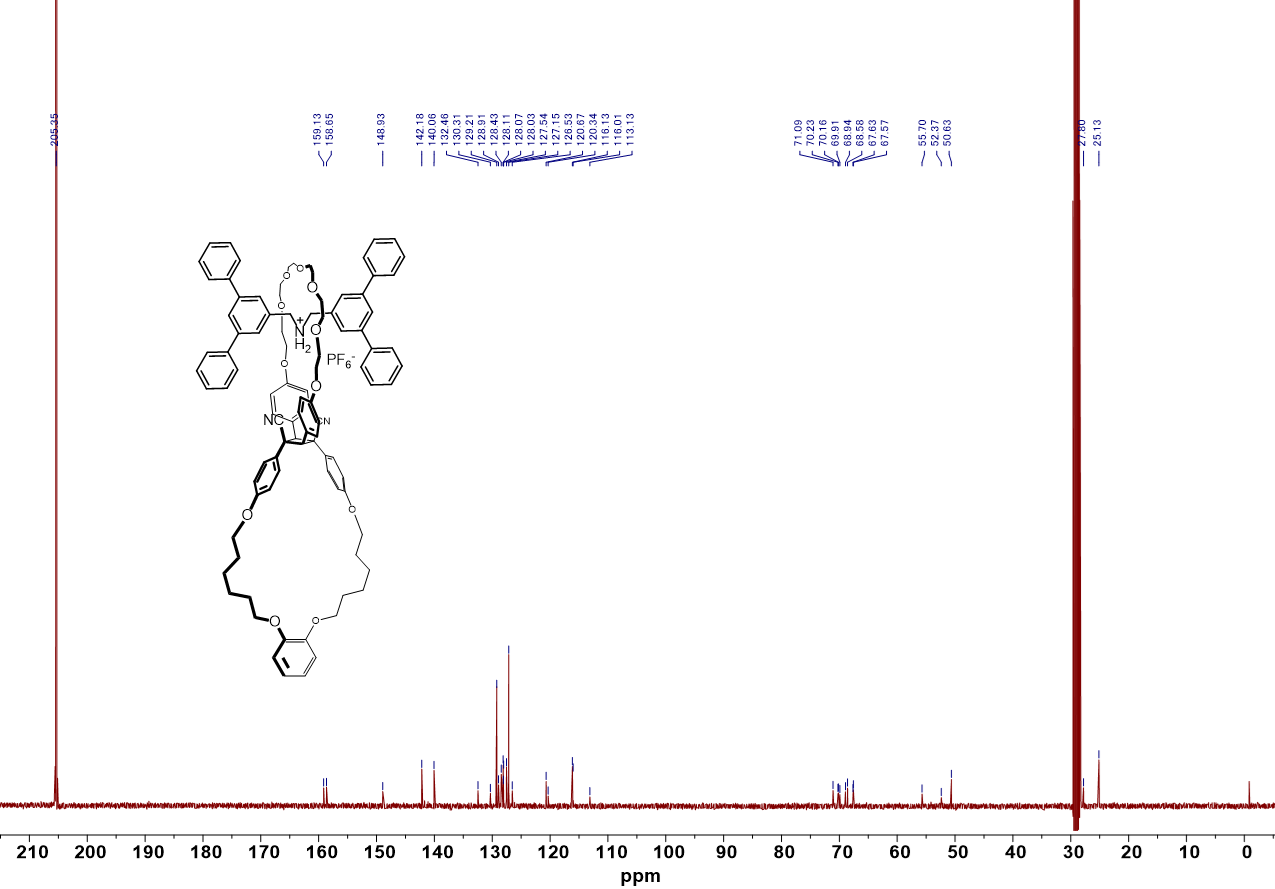


**Synthesis of c[2]RT(cMCH2@G)**


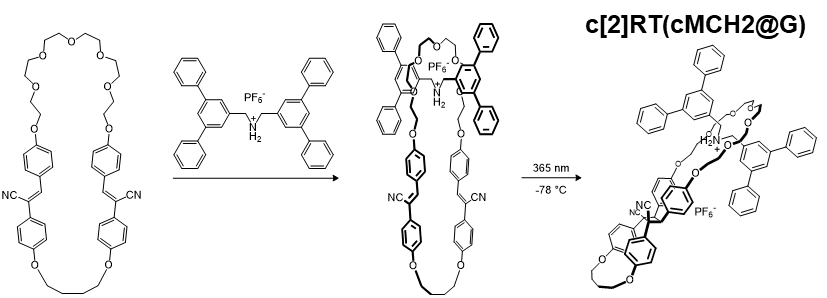


**MCH2** (78 mg, 0.1 mmol, 50 mM) and **G** (195 mg, 0.3 mmol) were dissolved in acetone (2 mL) and degassed by bubbling with N₂. The solution was then cooled to -78 °C using a low temperature reactor and irradiated with 365 nm UV light overnight. After irradiation, the solution was removed under reduced pressure without heating directly. The resulting residue was purified by silica column chromatography (CH_2_Cl_2_/Ethyl acetate = 100/0 – 88/12, gradient elution). **c[2]RT(cMCH1@G)** was isolated as white solid (132 mg, 92%). ^1^H NMR (600 MHz, ACETONE-*D*_6_) δ 8.23 (br s, 2H), 8.08 (t, *J* = 1.7 Hz, 1H), 7.94 (d, *J* = 1.7 Hz, 2H), 7.87 (d, *J* = 1.7 Hz, 2H), 7.30 (dd, *J* = 8.8, 2.6 Hz, 2H), 7.86 – 7.82 (m, 8H), 7.61 – 7.51 (m, 8H), 7.49 – 7.39 (m, 4H), 7.30 (dd, *J* = 8.8, 2.6 Hz, 2H), 7.18 (dd, *J* = 8.7, 2.6 Hz, 2H), 7.15 (d, *J* = 8.8 Hz, 4H), 6.77 (dd, *J* = 8.7, 2.8 Hz, 2H), 6.71 (dd, *J* = 8.7, 2.7 Hz, 2H), 6.66 (d, *J* = 1.7 Hz, 4H), 5.20 (s, 2H), 4.76 – 4.80 (br, 2H), 4.67 – 4.63 (br, 2H), 4.27 (s, 4H), 3.96 – 4.05 (m, 4H), 3.80 –3.76 (br, 2H), 3.74 – 3.69 (br, 2H), 3.69 – 3.63 (m, 4H), 3.62 – 3.58 (br, 2H), 3.53 – 3.48 (br, 2H), 3.47 – 3.42 (m, 4H), 3.37 – 3.34 (m, 4H), 1.63 (br, 4H). ^13^C NMR (126 MHz, ACETONE-*D*_6_) δ 170.09, 157.23, 142.51, 142.29, 140.02, 139.90, 131.20, 130.69, 129.24, 128.15, 128.12, 127.93, 127.86, 127.56, 127.21, 126.82, 126.58, 125.59, 119.55, 117.29, 116.79, 113.70, 70.97, 70.44, 70.18, 70.05, 69.93, 67.24, 65.89, 59.71, 55.38, 53.06, 52.95, 44.61, 21.12, 13.67. HR-MS (ESI): calcd for [M – PF_6_]^+^ , [C_84_H_82_N_3_O_9_]^+^ , m/z = 1276.6046, found m/z = 1276.5996.

^1^H NMR (600 MHz, acetone-d6, 298 K) spectrum of **c[2]RT(cMCH2@G)**


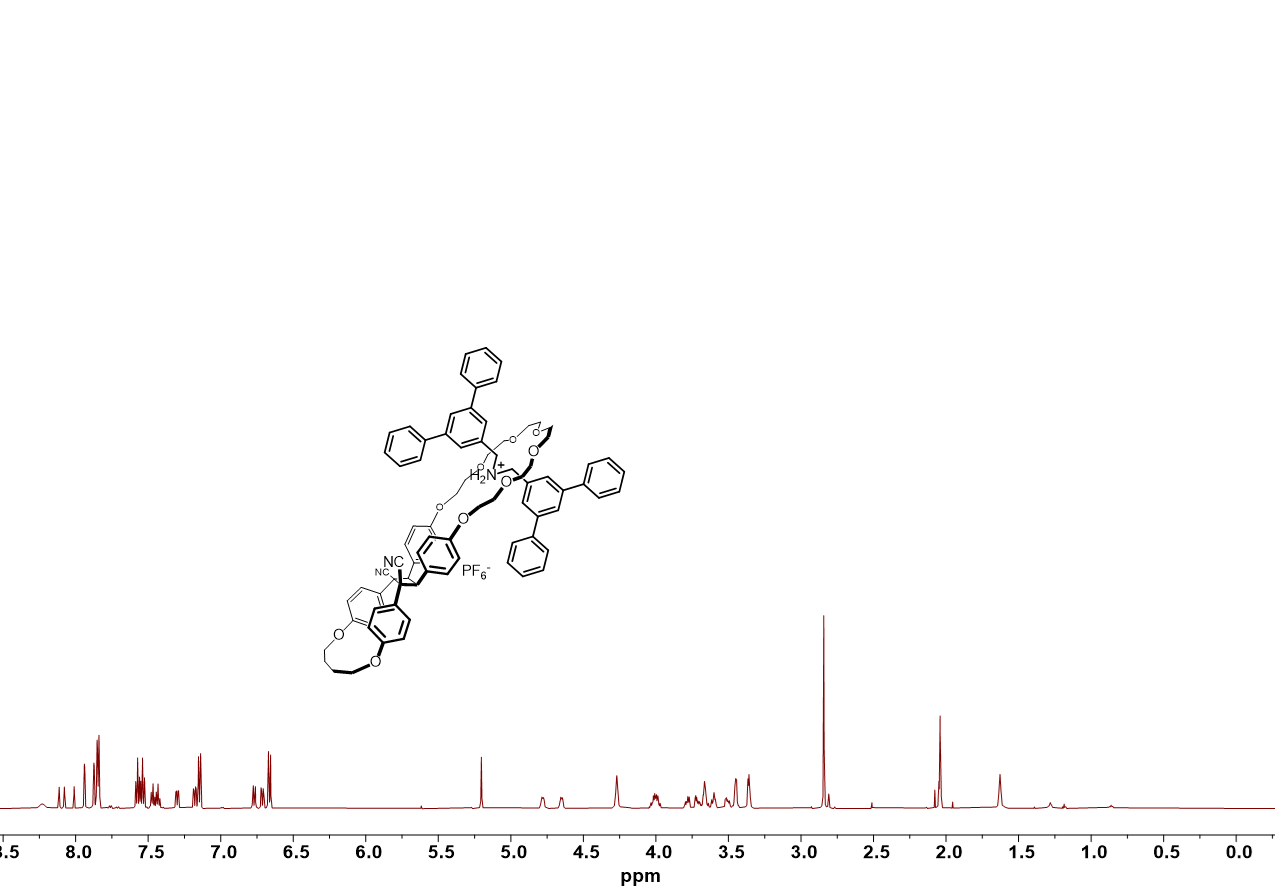


^13^C NMR (125 MHz, acetone-d6, 298 K) spectrum of **c[2]RT(cMCH2@G)**


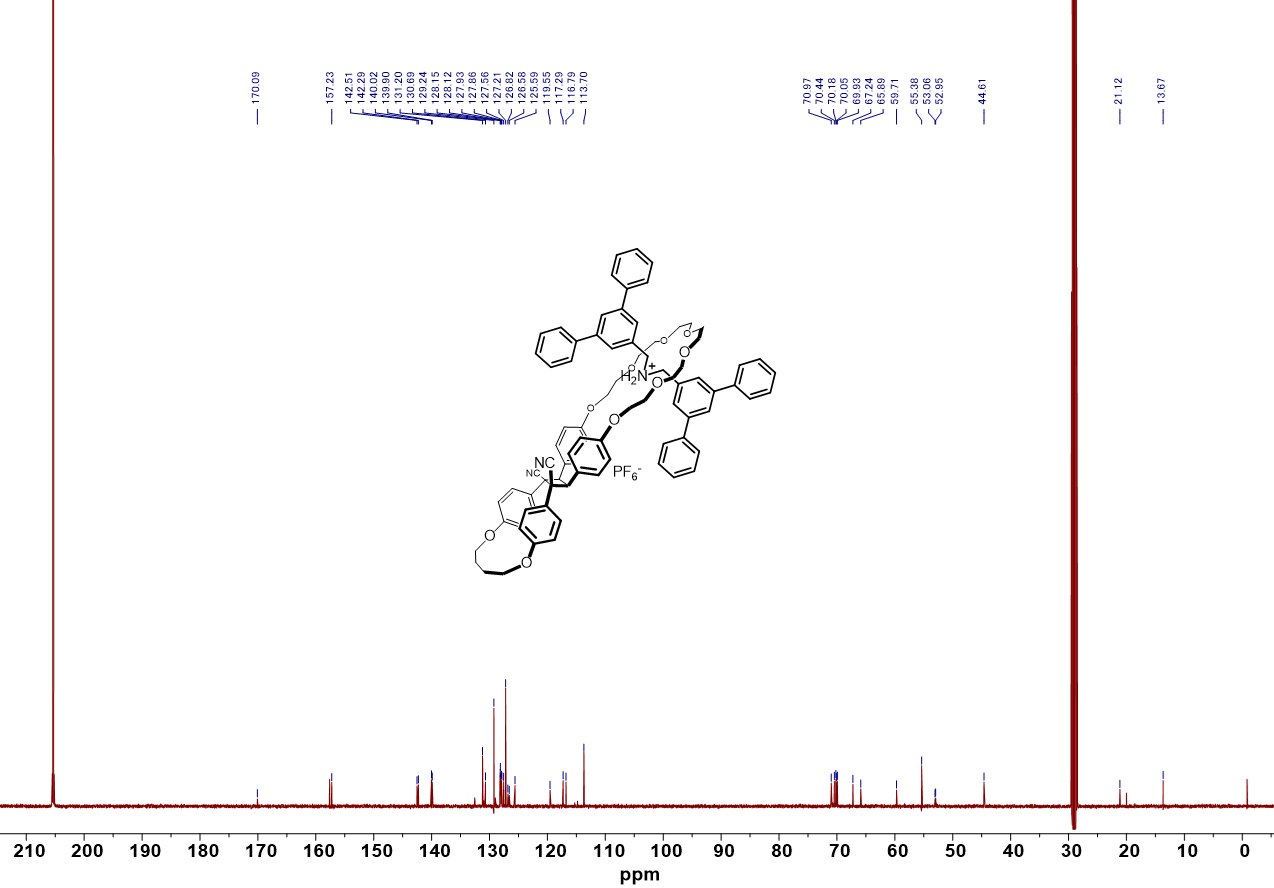


**Synthesis of bis-c[2]RT(cMCH3@G) and of bis-oRT(oMCH3@G)**


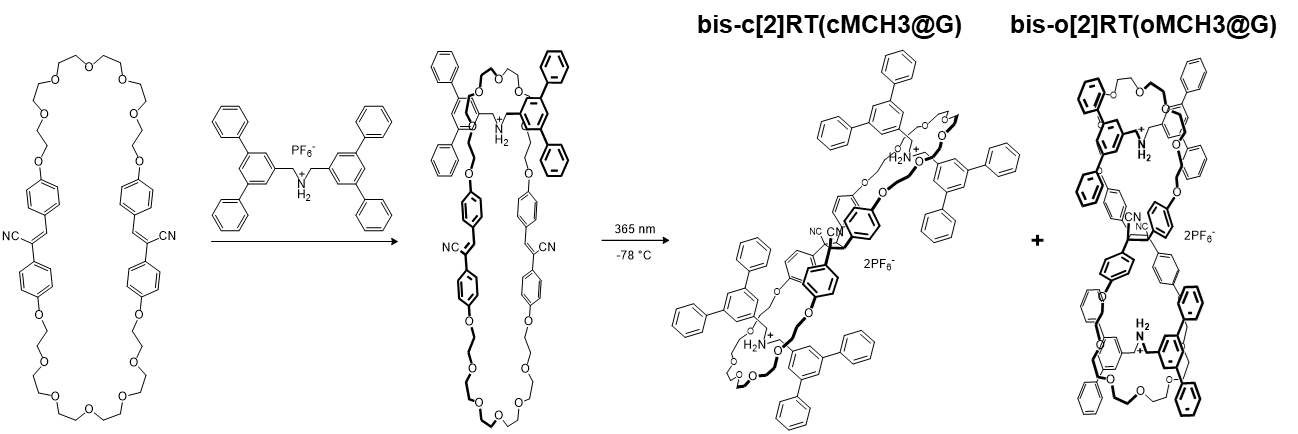


**MCH1** (97 mg, 0.1 mmol, 50 mM) and **G** (227.4 mg, 0.35 mmol) were dissolved in acetone (2 mL) and degassed by bubbling with N₂. The solution was then cooled to -78 °C using a low temperature reactor and irradiated with 365 nm UV light for 3 days. After irradiation, the solution was removed under reduced pressure without heating directly. The resulting residue was purified by silica column chromatography (CH_2_Cl_2_/Ethyl acetate = 100/0 – 88/12, gradient elution). **bis-c[2]RT(cMCH3@G)** and **bis-oRT(oMCH3@G)** were isolated as white solid (**bis-c[2]RT(cMCH3@G)**: 156 mg, 69%; **o[2]RT(oMCH1@G)**: 5.3 mg, 2.3%; Mixture: 19.7 mg, 8.7%; In total: 80%).

*Note: Partial overlap of* ***bis-c[2]RT(cMCH3@G)*** *and* ***bis-oRT(oMCH3@G)*** *during column elution led to little loss in yield of pure compounds.*

**bis-c[2]RT(cMCH3@G)**: ^1^H NMR (600 MHz, ACETONE-*D*_6_) δ 8.19 (br s, 2H), 8.15 – 8.06 (m, 4H), 7.95 – 7.82 (m, 24H), 7.61 – 7.52 (m, 16H), 7.50 – 7.45 (m, 4H), 7.45 – 7.40 (m, 4H), 7.14 (d, *J* = 8.9 Hz, 1H), 7.07 (d, *J* = 8.8 Hz, 1H), 6.64 (d, *J* = 8.8 Hz, 1H), 6.55 (d, *J* = 8.9 Hz, 1H), 5.02 (s, 2H), 4.78 – 4.74 (br, 2H), 4.66 – 4.62 (br, 2H), 4.60 – 4.57 (br, 2H), 4.05 – 3.97 (m, 8H), 3.91 – 3.87 (m, 4H), 3.80 – 3.72 (m, 8H), 3.69 – 3.58 (m, 12H), 3.54 – 3.42 (m, 10H), 3.37 – 3.33 (m, 4H), 3.31 – 3.27 (m, 4H). ^13^C NMR (101 MHz, ACETONE-*D*_6_) δ 158.77, 157.65, 142.50, 142.42, 142.30, 142.27, 140.03, 140.01, 139.89, 132.55, 131.12, 129.26, 129.23, 129.11, 128.24, 128.19, 128.17, 128.11, 127.96, 127.93, 127.59, 127.25, 127.22, 127.20, 126.82, 126.56, 126.53, 119.56, 114.43, 113.67, 70.96, 70.82, 70.54, 70.50, 70.41, 70.17, 70.05, 69.94, 67.65, 67.23, 59.72, 54.12, 46.55, 20.01, 13.67. HR-MS (ESI): calcd for [M – 2PF_6_]^+^ , [C₁₃₀H₁₃₀N₄O₁₄]^2+^ , m/z = 985.9803, found m/z = 985.9810.

**bis-oRT(oMCH3@G)**: ^1^H NMR (600 MHz, ACETONE-*D*_6_) δ 8.33 (br s, 2H), 8.04 – 8.00 (m, 4H), 7.83 (m, 8H), 7.80 (m, 8H), 7.76 (m, 8H), 7.53 (m, 8H), 7.45 (m, 16H), 7.33 (d, *J* = 8.4 Hz, 4H), 7.30 (d, *J* = 8.6 Hz, 4H), 7.06 (d, *J* = 8.7 Hz, 4H), 6.99 (d, *J* = 8.6 Hz, 4H), 4.61 – 4.56 (br, 4H), 4.40 – 4.37 (br, 4H), 4.31 (s, 2H), 4.21 – 4.18 (m, 4H), 4.08 – 4.04 (br, 4H), 3.88 – 3.85 (m, 4H), 3.85 – 3.81 (m, 4H), 3.76 (m, 4H), 3.64 (m, 4H), 3.53 (t, *J* = 5.3 Hz, 4H), 3.45 (m, 8H), 3.26 (m, 4H), 3.22 (m, 4H), 2.98 (m, 4H). HR-MS (ESI): calcd for [M – 2PF_6_]^+^ , [C₁₃₀H₁₃₀N₄O₁₄]^+^ , m/z = 985.9803, found m/z = 985.9805.

^1^H NMR (600 MHz, acetone-d6, 298 K) spectrum of **bis-c[2]RT(cMCH3@G)**

**
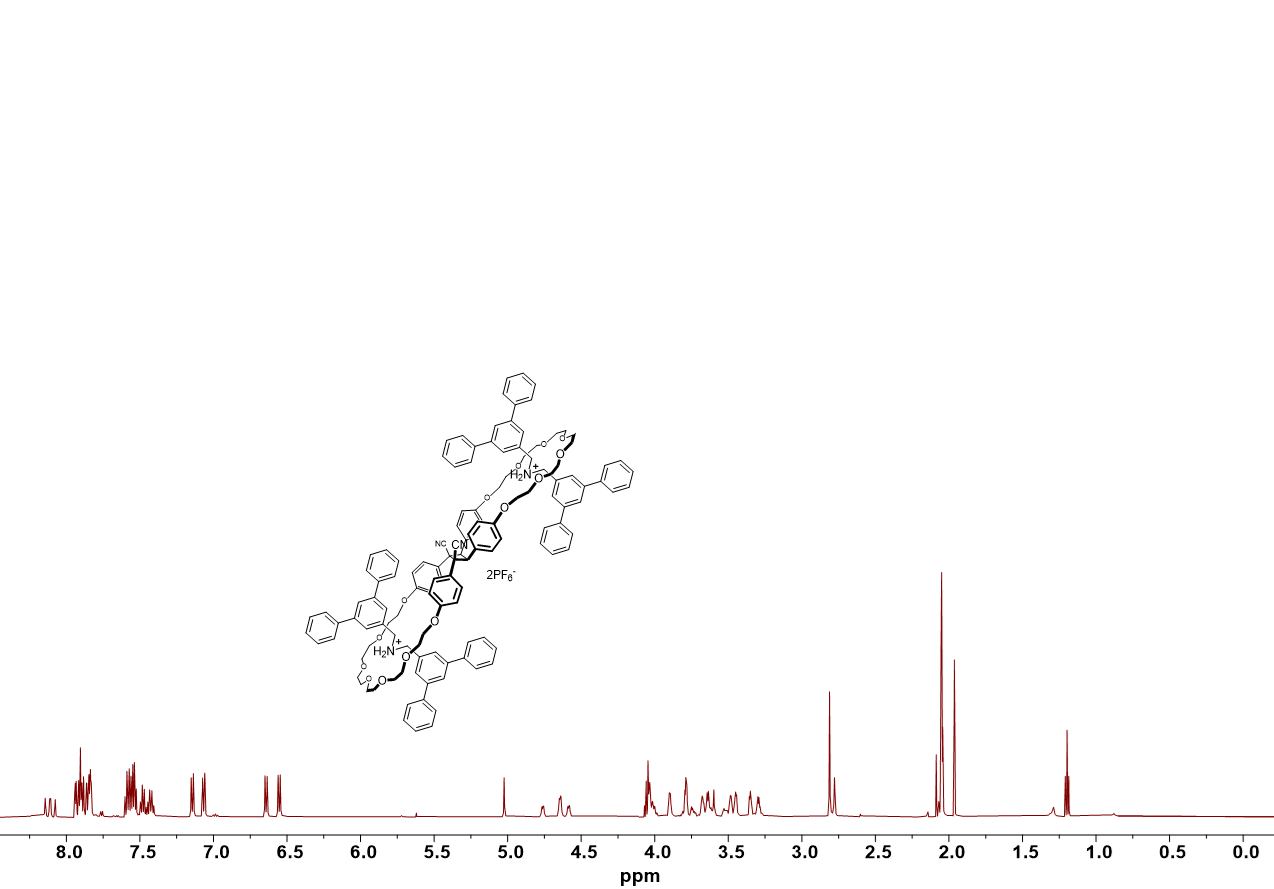
**

^13^C NMR (100 MHz, acetone-d6, 298 K) spectrum of **bis-c[2]RT(cMCH3@G)**

**
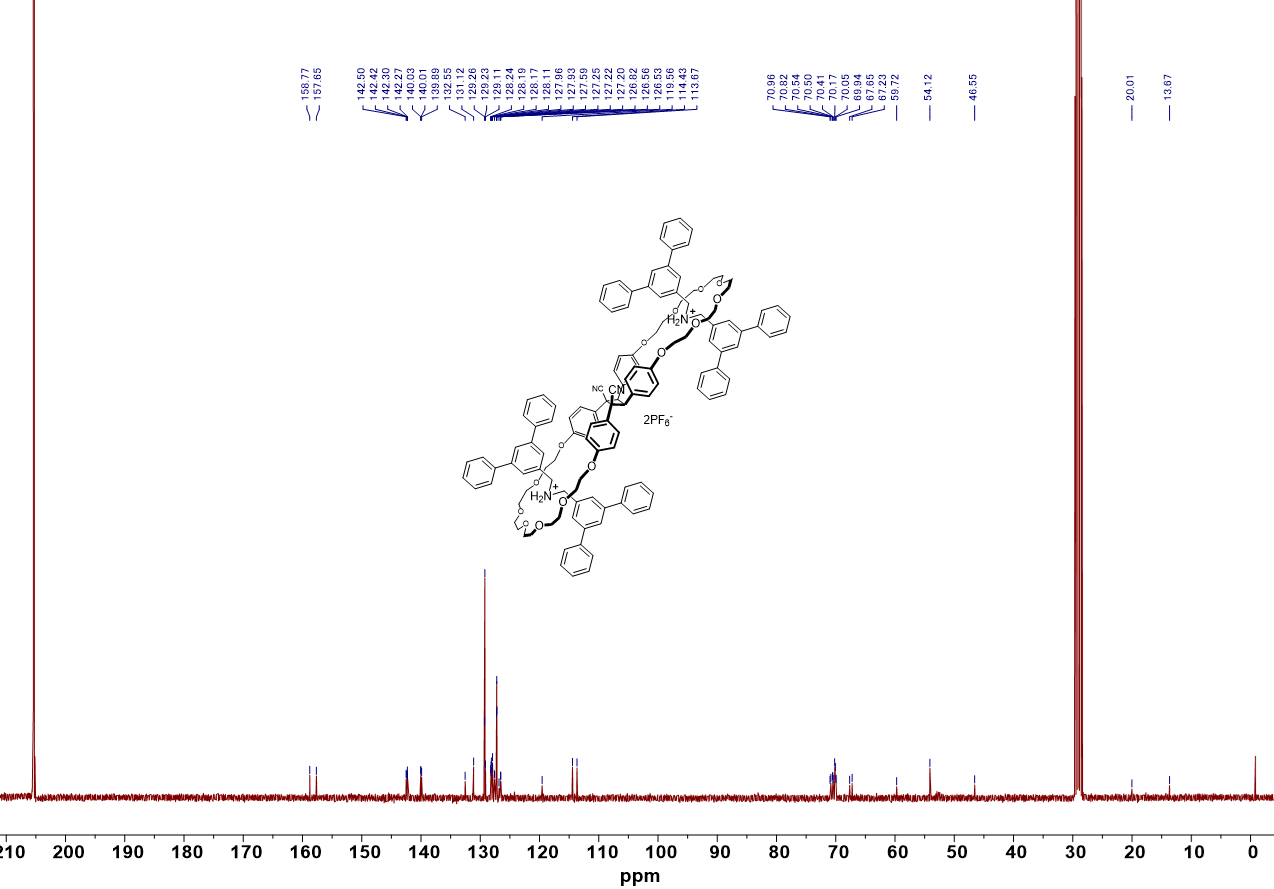
**

^1^H NMR (600 MHz, acetone-d6, 298 K) spectrum of **c[3]RT(oMCH3@G)**

**
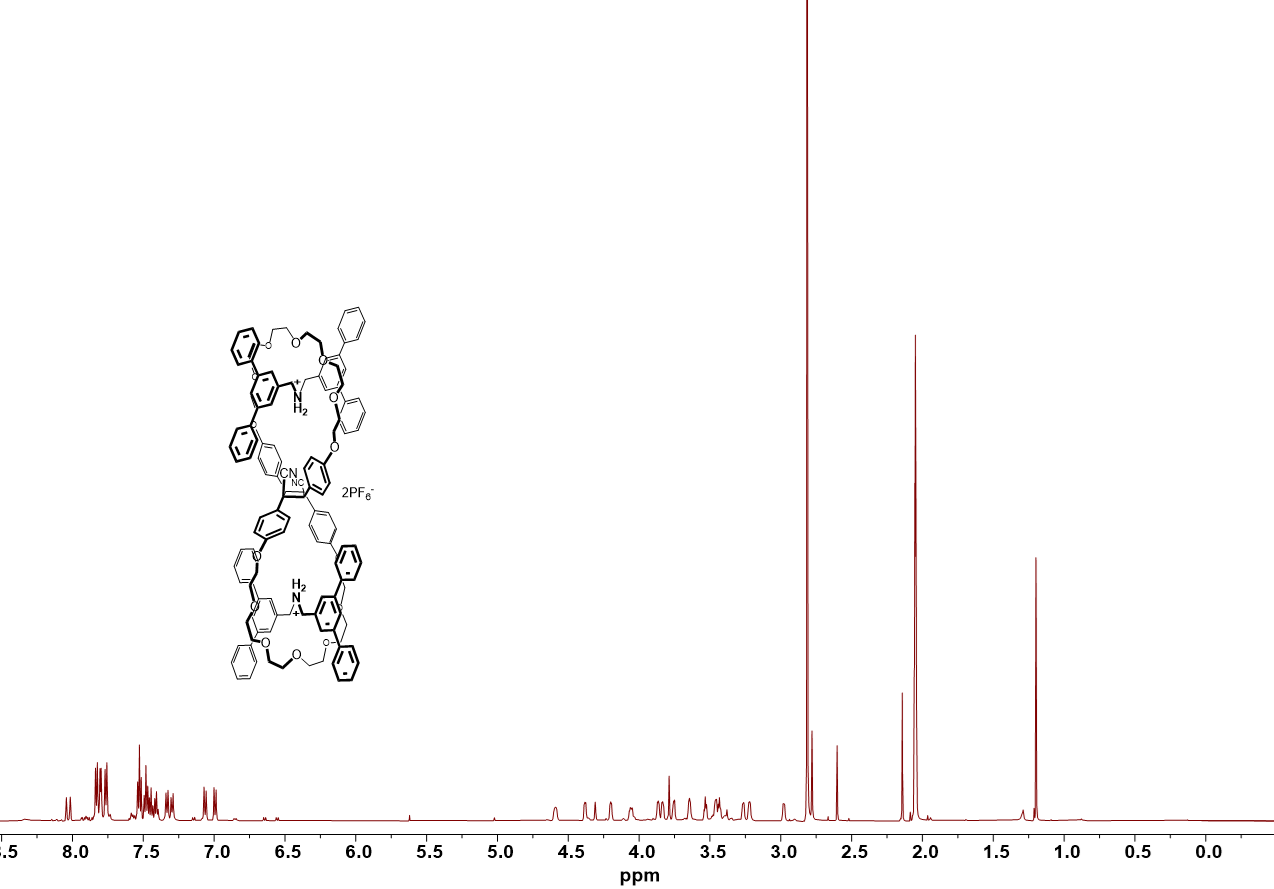
**

^13^C NMR (100 MHz, acetone-d6, 298 K) spectrum of **bis-oRT(oMCH3@G)**

**
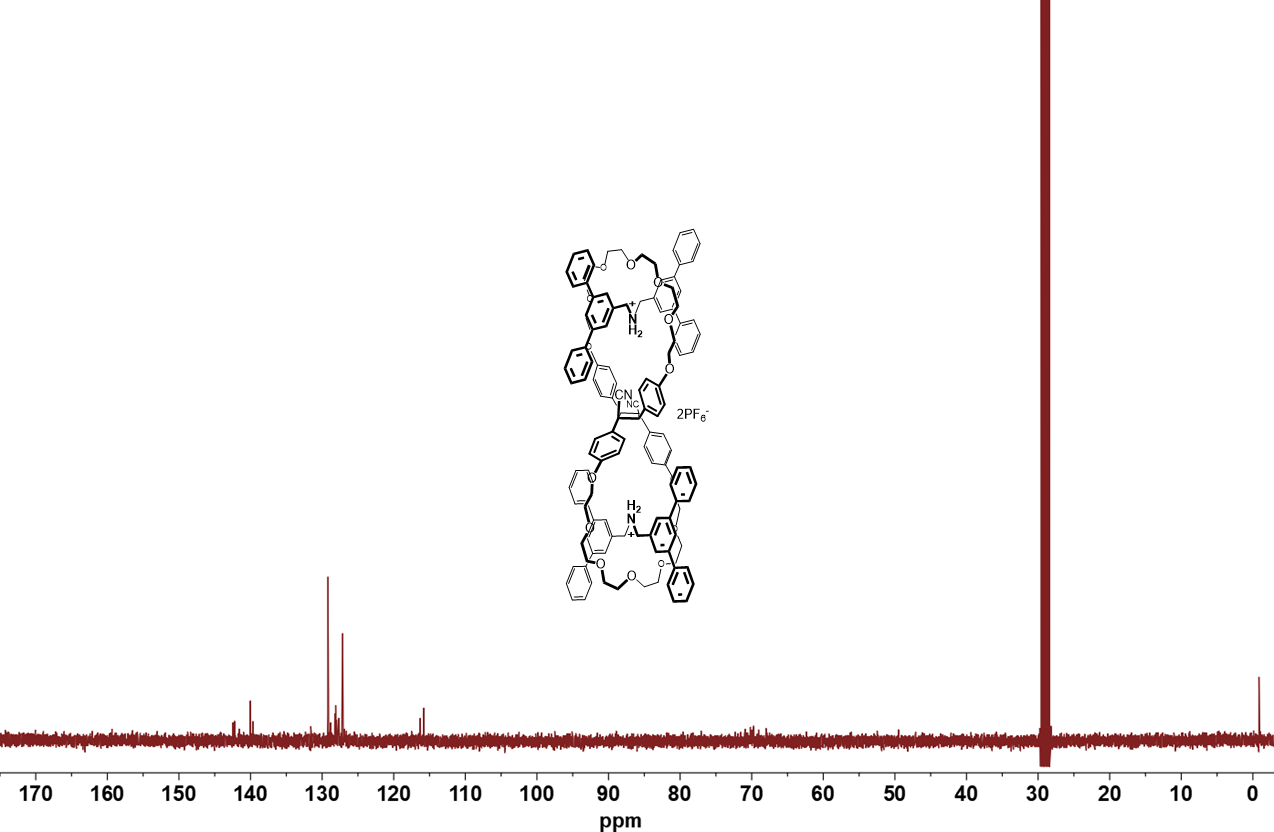
**

**2. Topology transformation of MCH1, MCH2, and MCH3 from macrocycles to chair-like and orthogonal figure-eight structures**

**2.1 MCH1**

**
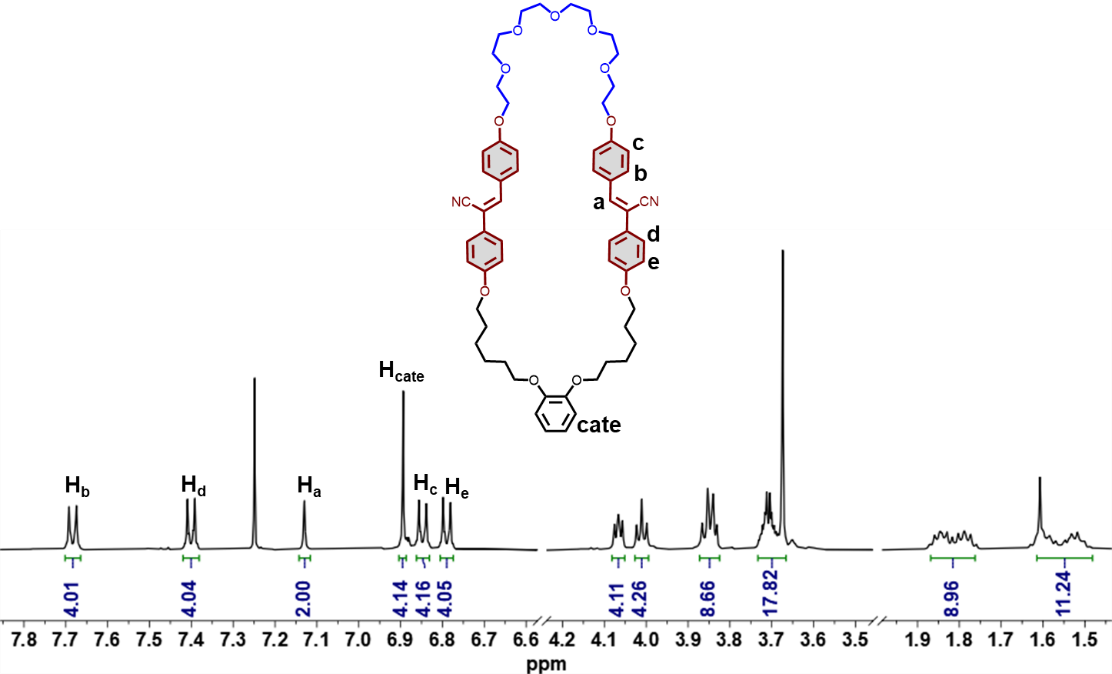
**

**Figure S1.** ^1^H spectrum of **MCH1**; 298 K, CHCl_3_-d.

**
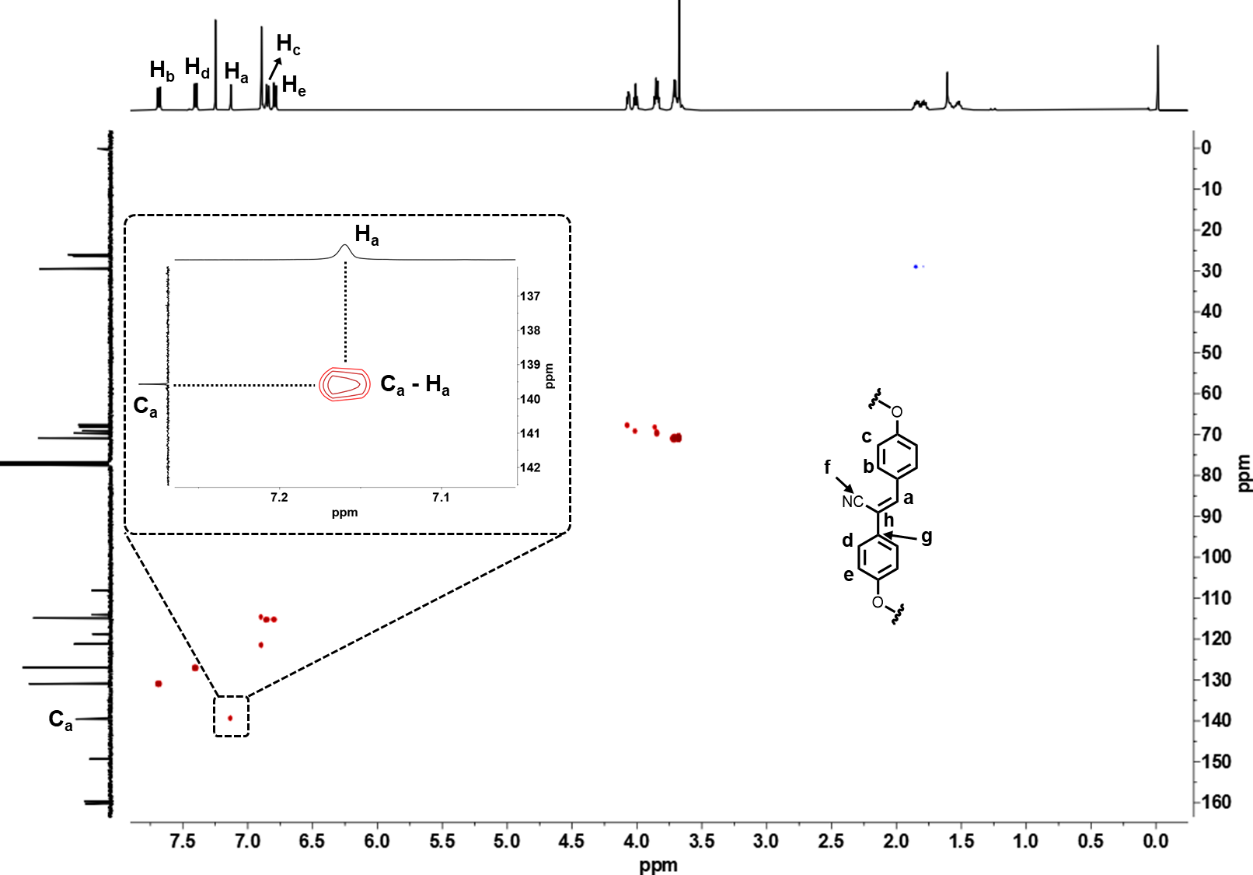
**

**Figure S2.** HSQC spectrum of **MCH1**; 298 K, CHCl_3_-d.

**
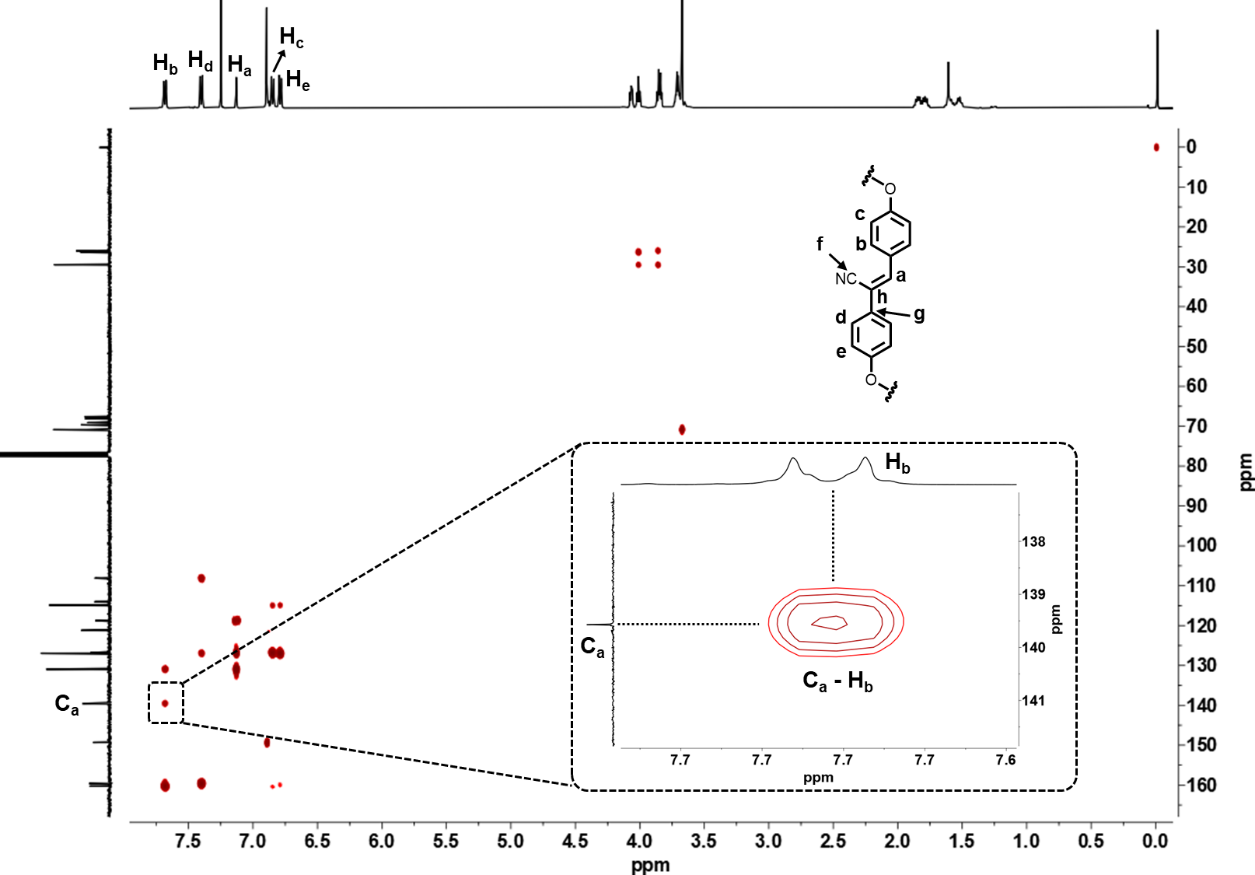
**

**Figure S3.** HMBC spectrum of **MCH1**; 298 K, CHCl_3_-d.

**
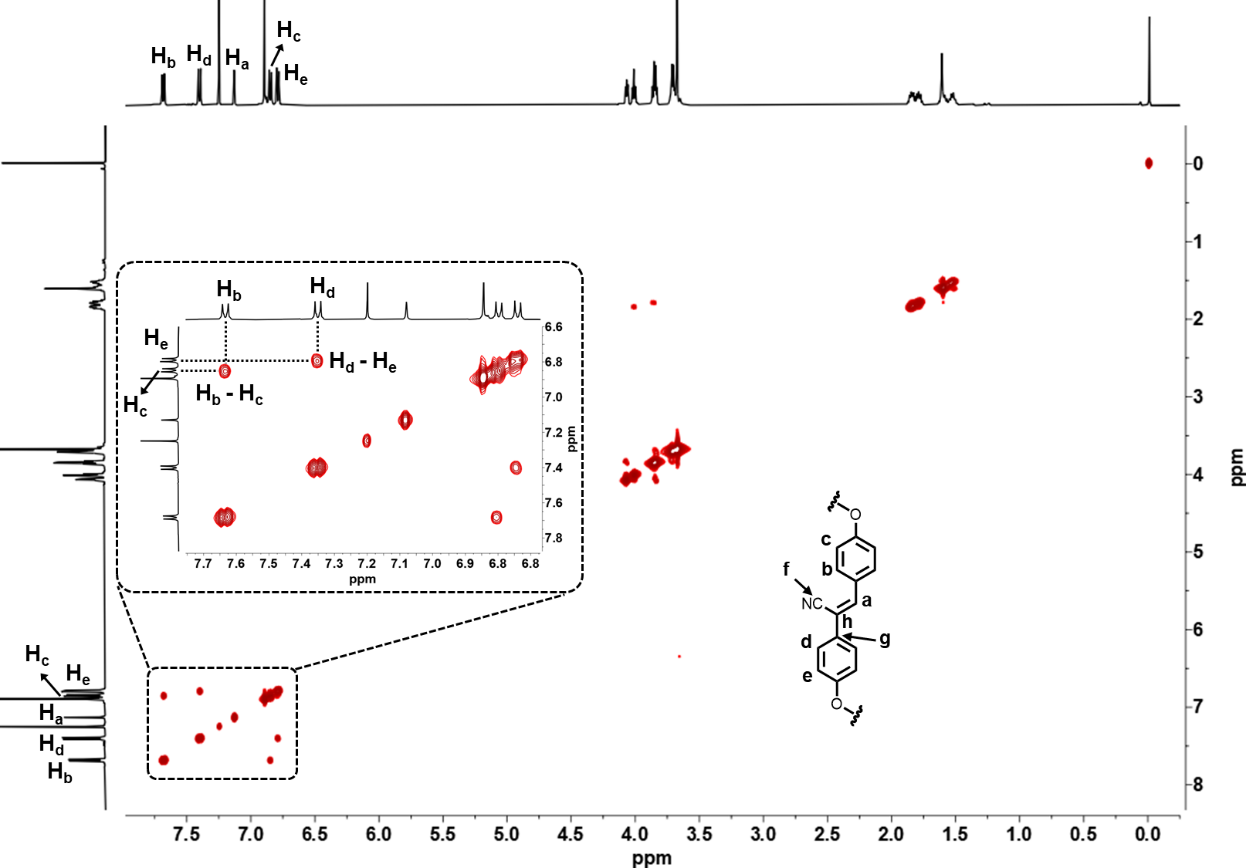
**

**Figure S4.** COSY spectrum of **MCH1**; 298 K, CHCl_3_-d.

**Photoisomerization of MCH1 to cMCH1** **and oMCH1**

**
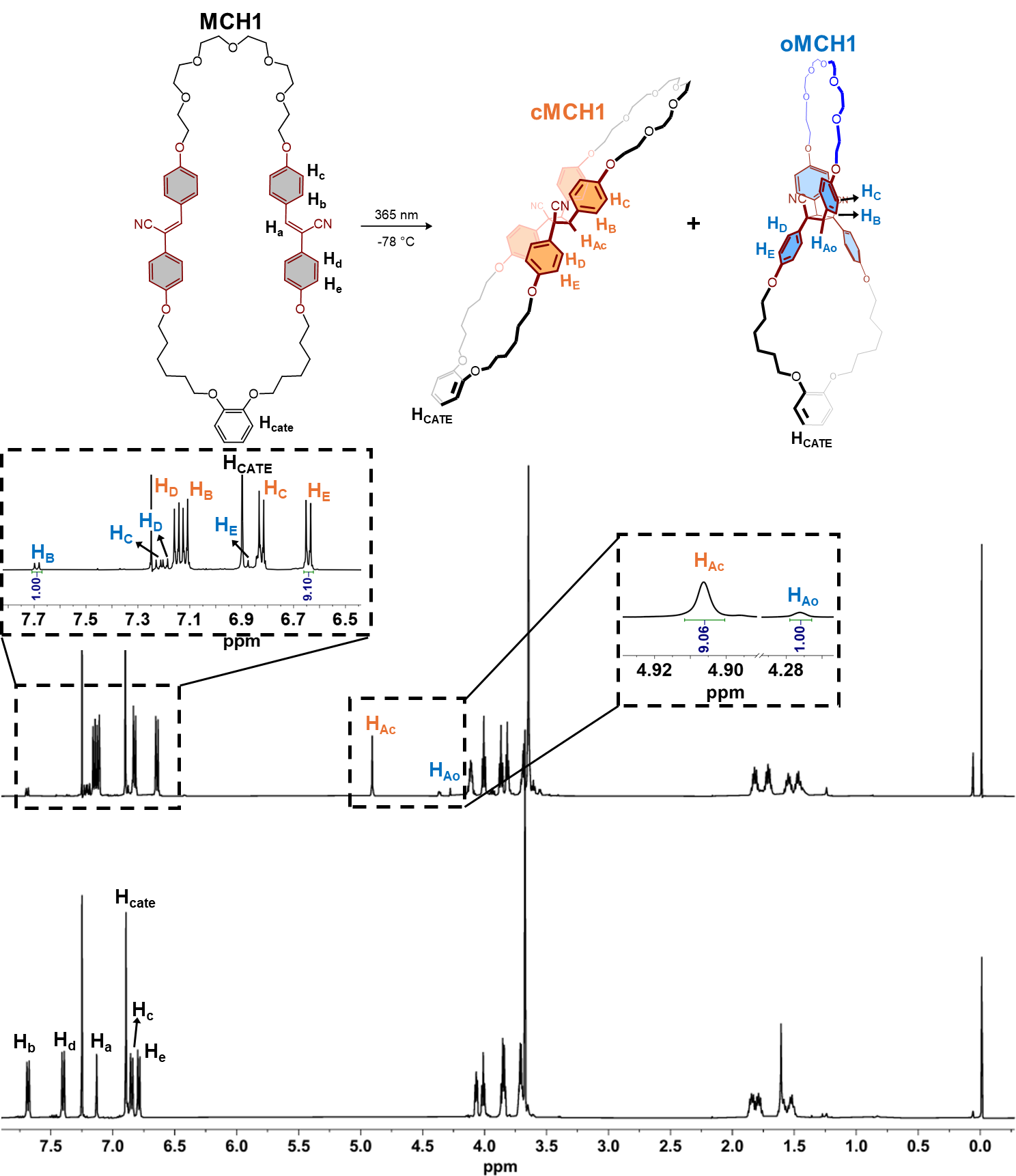
**

**Figure S5.** Photoisomerization and intramolecular [2+2] photocycloaddition of **MCH1**. ^1^H NMR spectrum of **MCH1** (bottom) and **MCH1** after irradiation by 365 nm UV light at -78 °C overnight (above); 298 K, CHCl_3_-d.

**Intramolecular [2+2] photocycloaddition between cyano-stilbene moieties in different arrangements**

**Scheme S9.** intramolecular [2+2] photocycloaddition of cyano-stilbene in a parallel head-to-head arrangement affords **cMCH1** (above) and intramolecular [2+2] photocycloaddition of cyano-stilbene in an anti-parallel head-to-tail arrangement affords **oMCH1** (bottom).

**Structural analysis cMCH1 by ^1^H NMR, HSQC, HMBC and COSY**

**cMCH1** is taken as an example for assignment of proton signals, and signal assignments for all other figure-eight compounds including rotaxanes follow such method:

Proton of cyclobutane (H_Ac_) can be first assigned according to previous reports ^12-14^. From the HSQC spectra, the unsubstituted carbon of cyclobutane (C_A_) is identified via the correlation of C_A_ – H_A_. The adjacent carbon signal lacking any correlation with cyclobutane protons was assigned as the cyano-substituted carbon (C_H_). In the subsequent HMBC spectrum, correlation of C_A_ – H_B_ and C_H_ – H_D_ allow the assignments of H_B_ and H_D_, respectively. Finally, the remaining aromatic protons (H_C_ and H_E_) can be assigned by COSY correlation of H_B_ – H_C_ and H_D_ – H_E_.

**
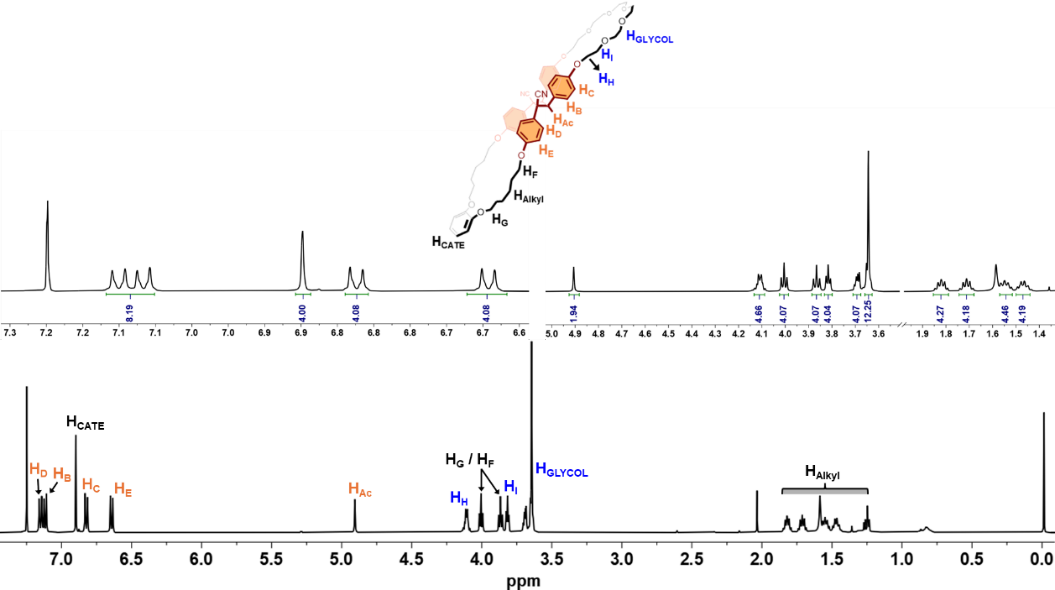
**

**Figure S6.** ^1^H NMR spectrum of **cMCH1**; 298 K, CHCl_3_-d.


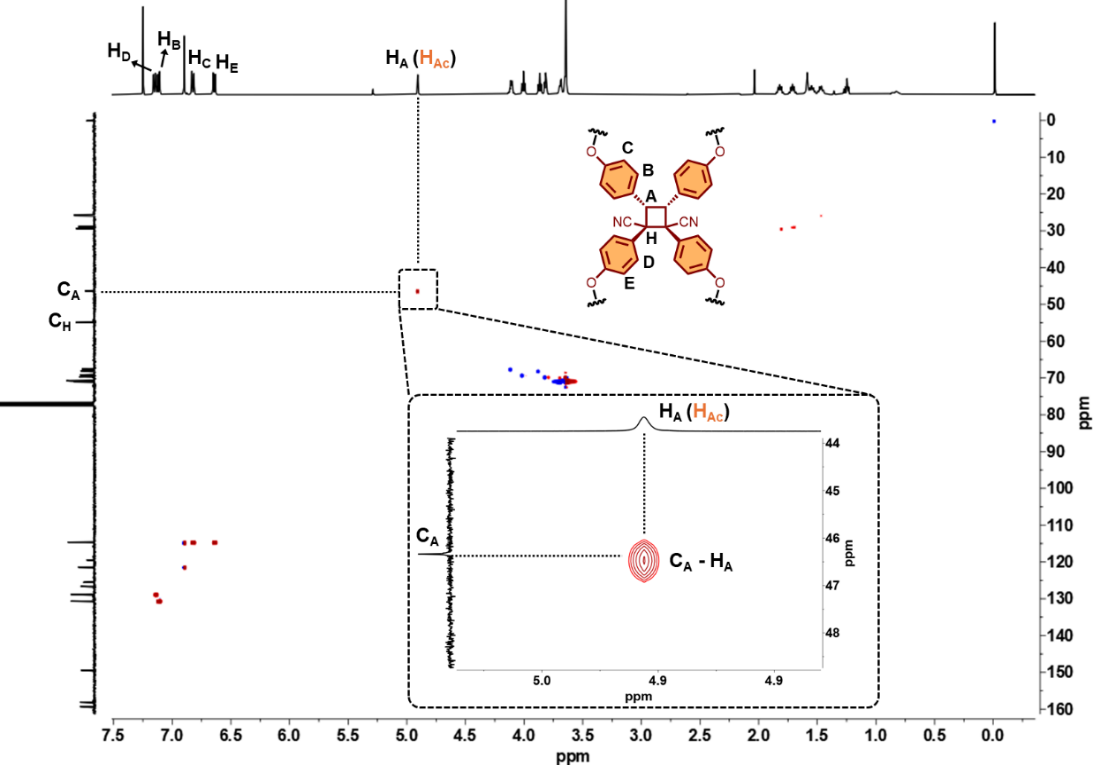


**Figure S7.** HSQC spectrum of **cMCH1**; 298 K, CHCl_3_-d.

**
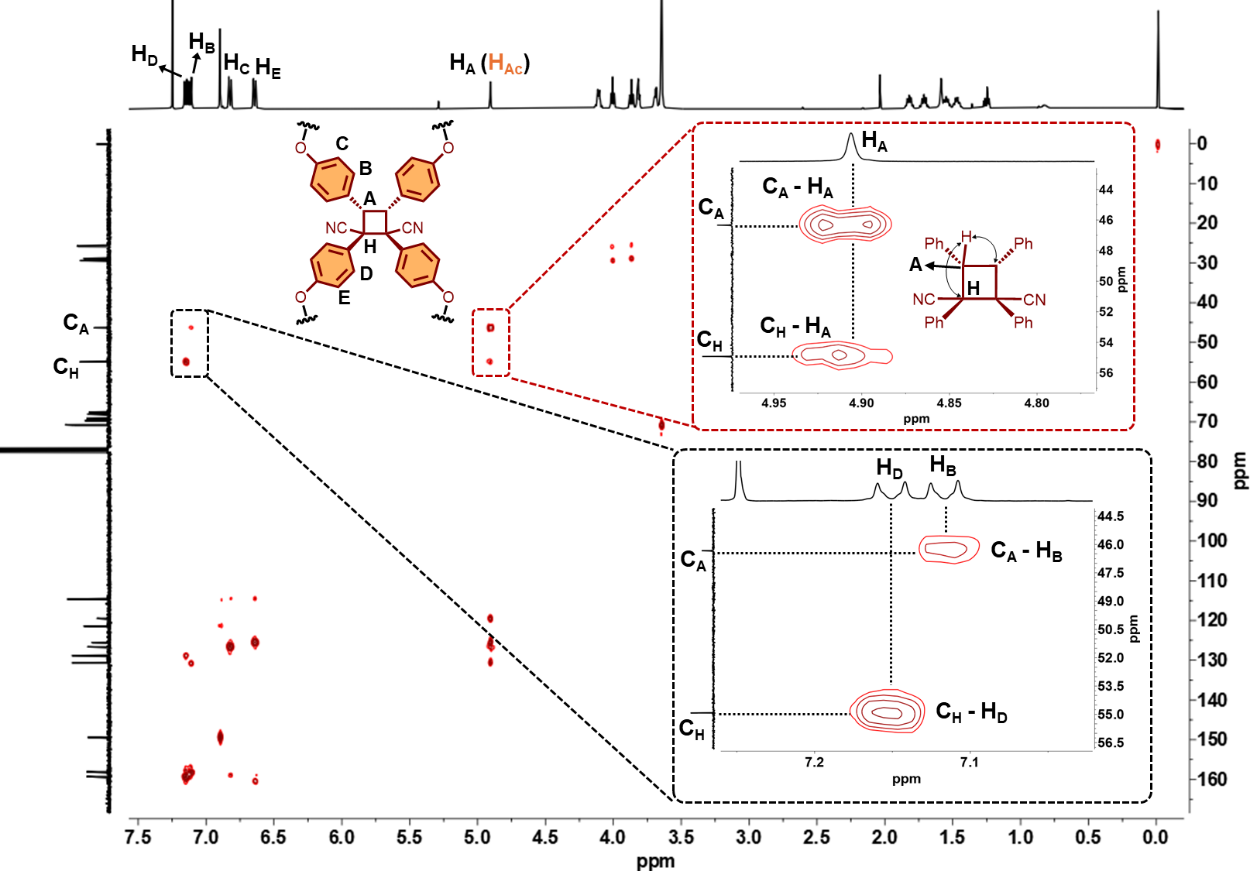
**

**Figure S8.** HMBC spectrum of **cMCH1**; 298 K, CHCl_3_-d.

**
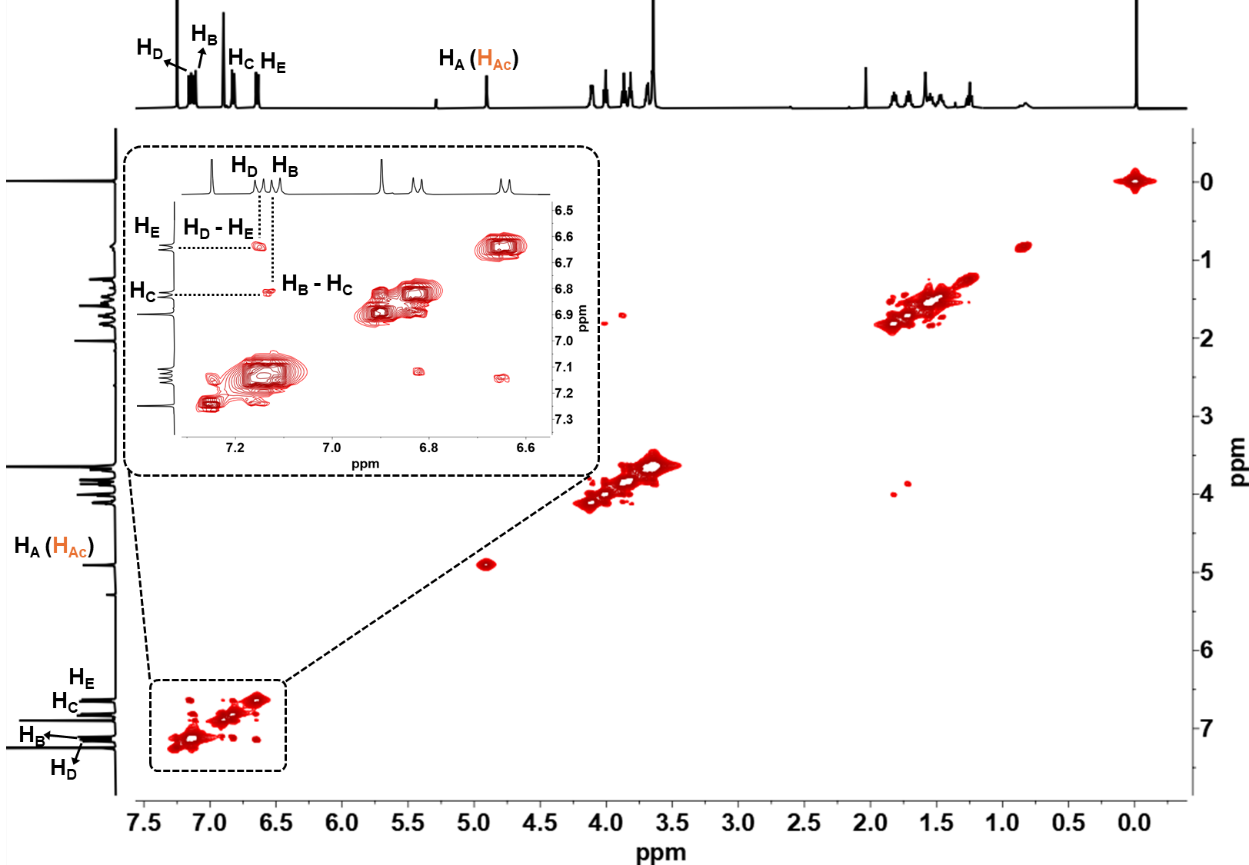
**

**Figure S9.** COSY spectrum of **cMCH1**; 298 K, CHCl_3_-d.

**Association of cMCH1 and dibenzylammonium (DBA^+^)**

The complexation of **cMCH1** with dibenzylammonium **(DBA^+^)** was investigated by NMR titration experiments. Association constant (K_a_) is determined as 325 M^-1^.

**
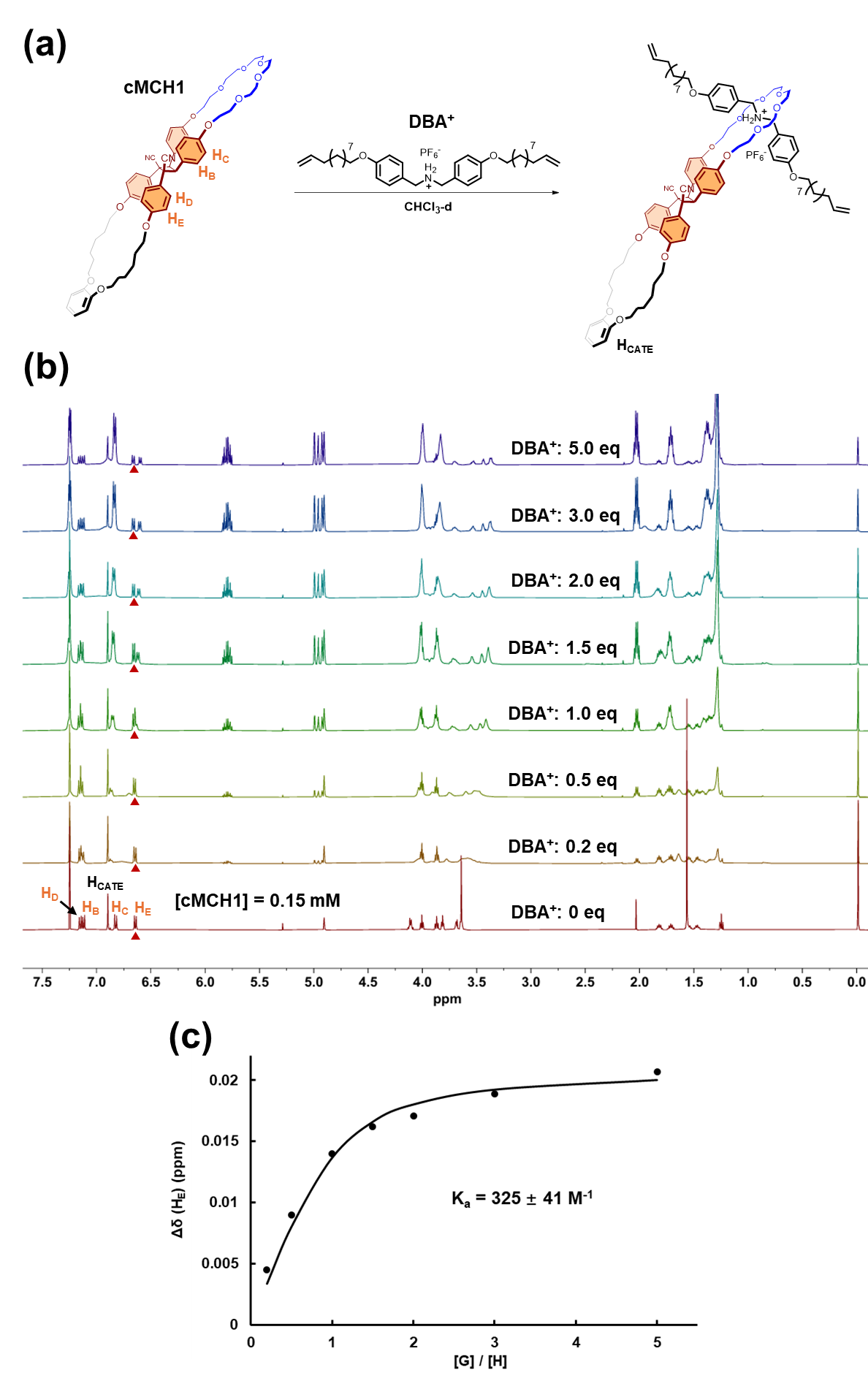
**

**Figure S10.** (a) Association of **cMCH1** and **DBA^+^**. (b) ^1^H NMR spectra of mixture of **cMCH1** and **DBA^+^** with increasing equivalence of **DBA^+^**. (c) Association constant (K_a_) between **cMCH1** and **DBA^+^**.

**Structural analysis oMCH1 by ^1^H NMR, HSQC, HMBC and COSY**

**
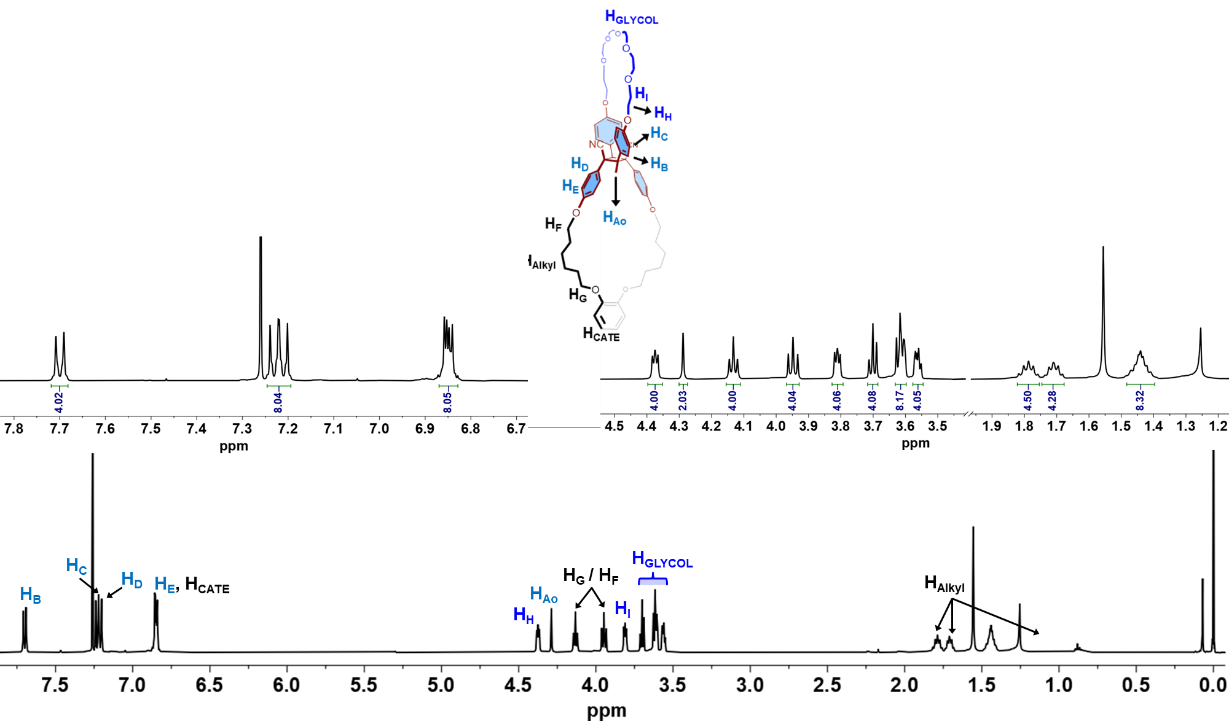
**

**Figure S11.** ^1^H NMR spectrum of **oMCH1**; 298 K, CHCl_3_-d.

**
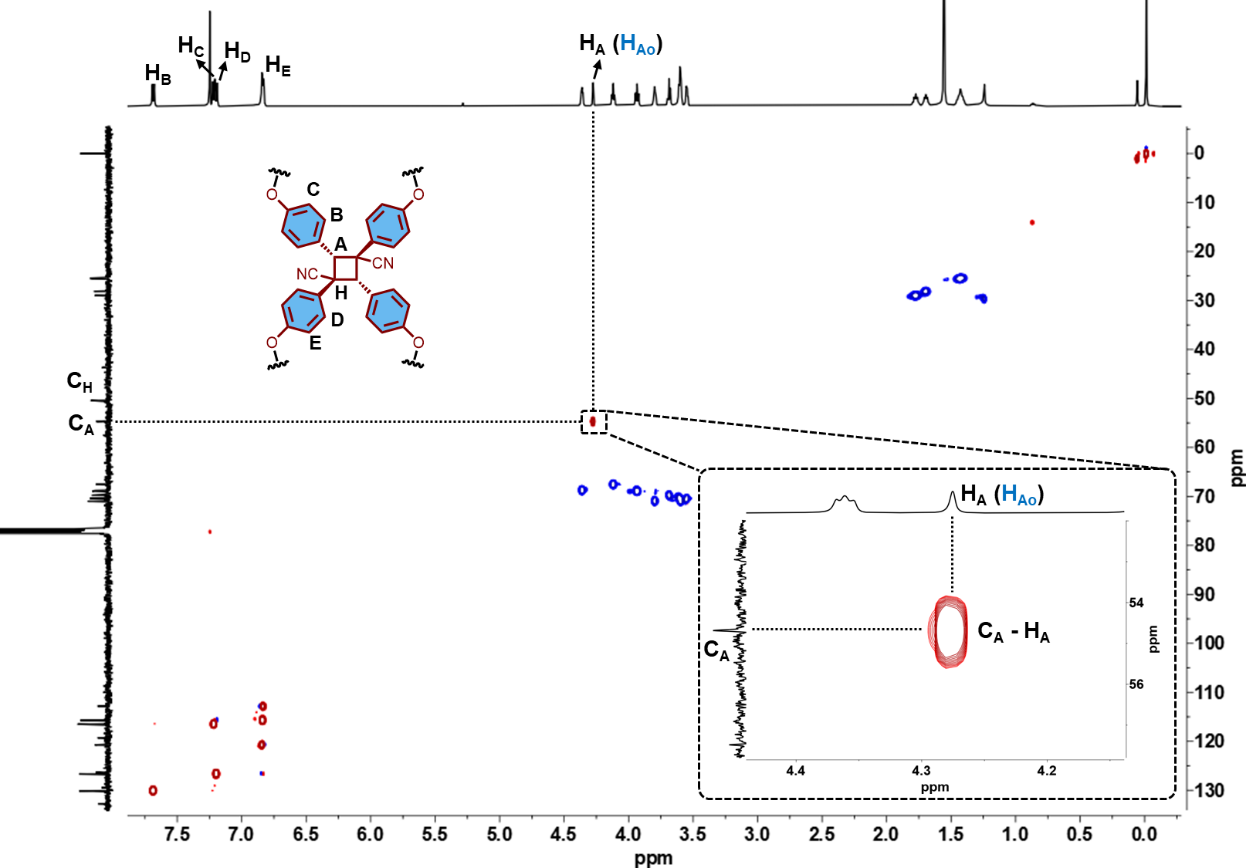
**

**Figure S12.** HSQC spectrum of **oMCH1**; 298 K, CHCl_3_-d.

**
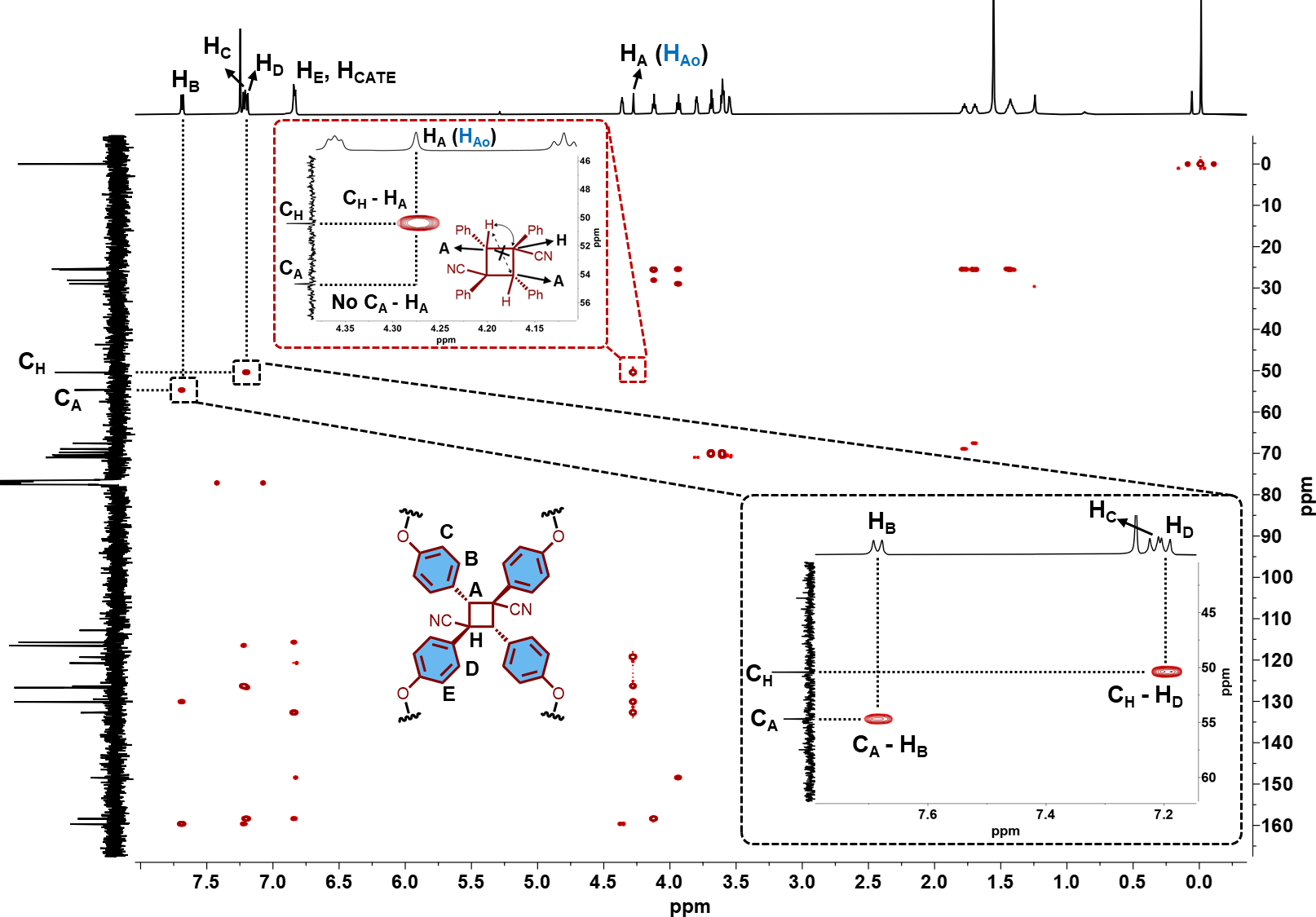
**

**Figure S13.** HMBC spectrum of **oMCH1**; 298 K, CHCl_3_-d.

**
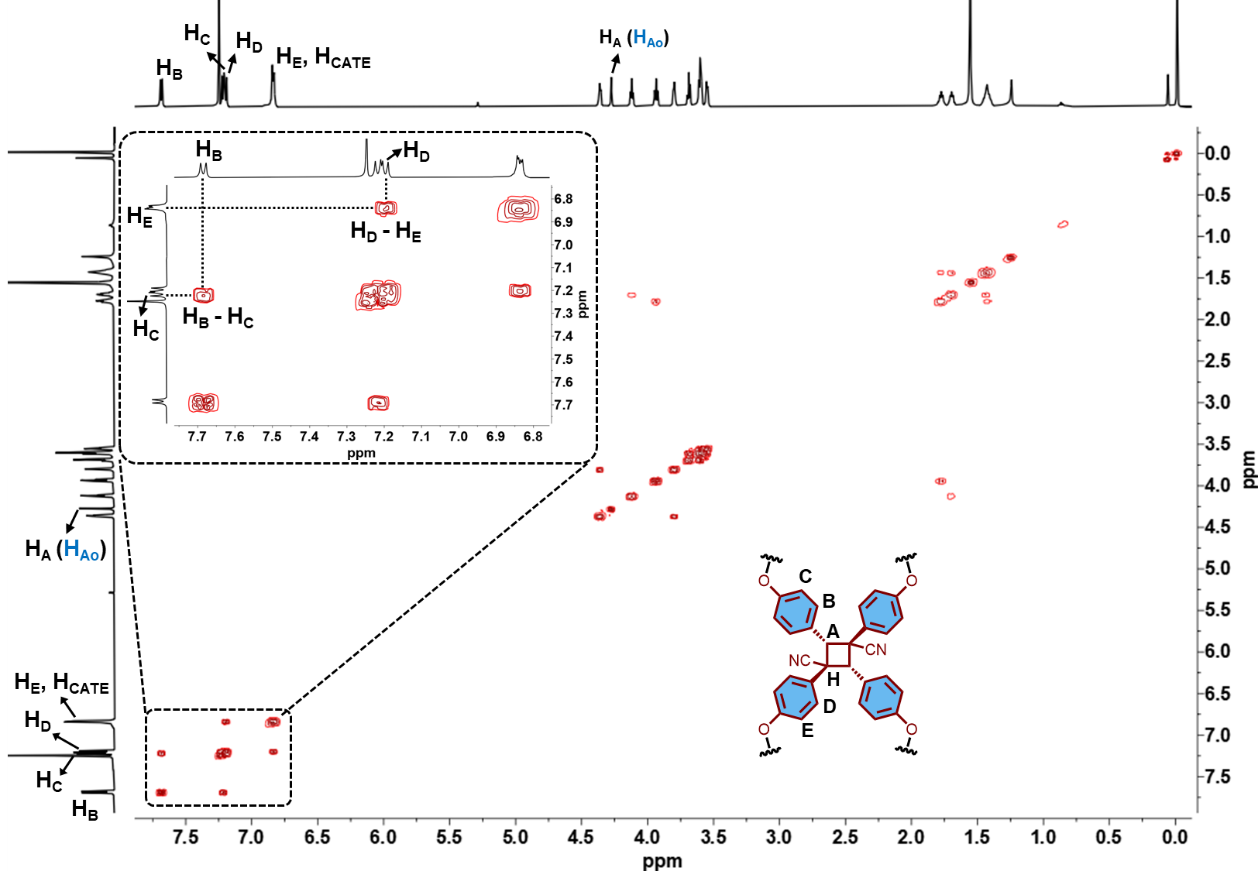
**

**Figure S14.** COSY spectrum of **oMCH1**; 298 K, CHCl_3_-d.

**2.2 MCH2**

**Structural analysis MCH2 by ^1^H NMR, HSQC, HMBC and COSY**

**
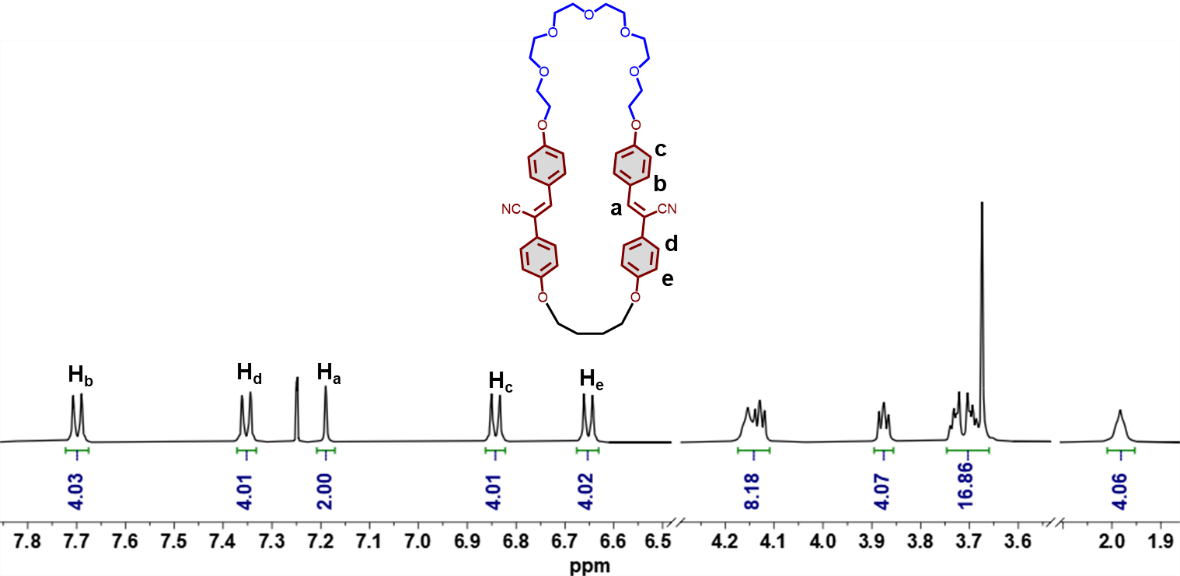
**

**Figure S15.** ^1^H NMR spectrum of **MCH2**; 298 K, CHCl_3_-d.

**
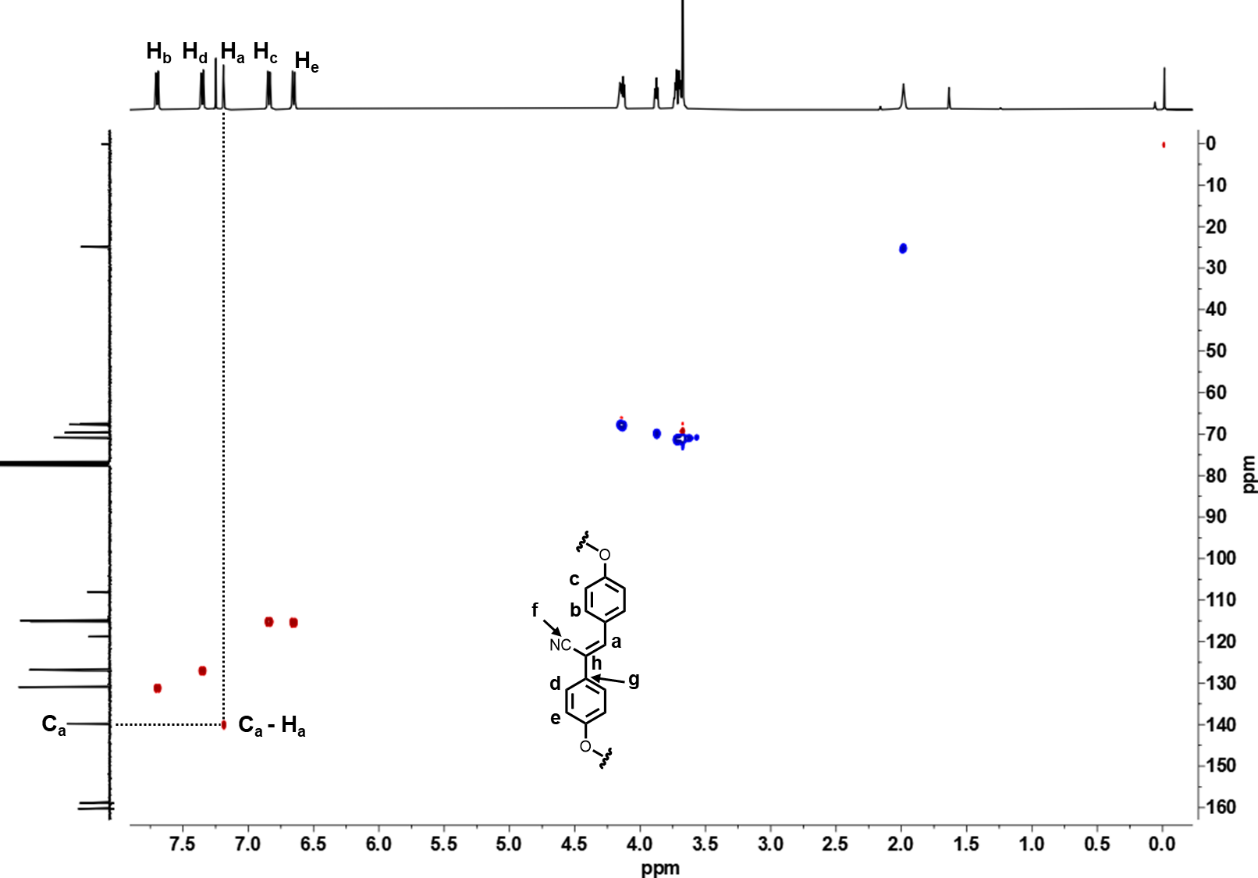
**

**Figure S16.** HSQC spectrum of **MCH2**; 298 K, CHCl_3_-d.

**
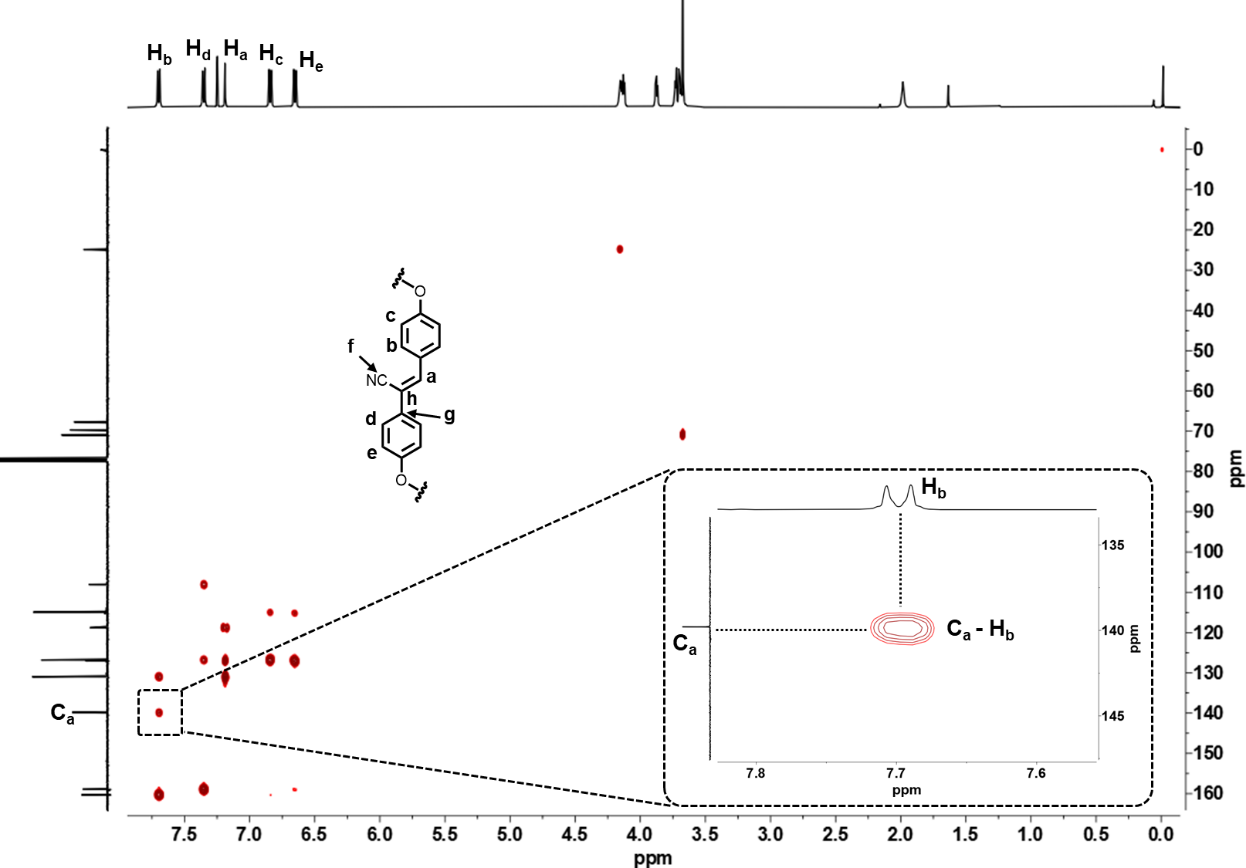
**

**Figure S17.** HMBC spectrum of **MCH2**; 298 K, CHCl_3_-d.

**
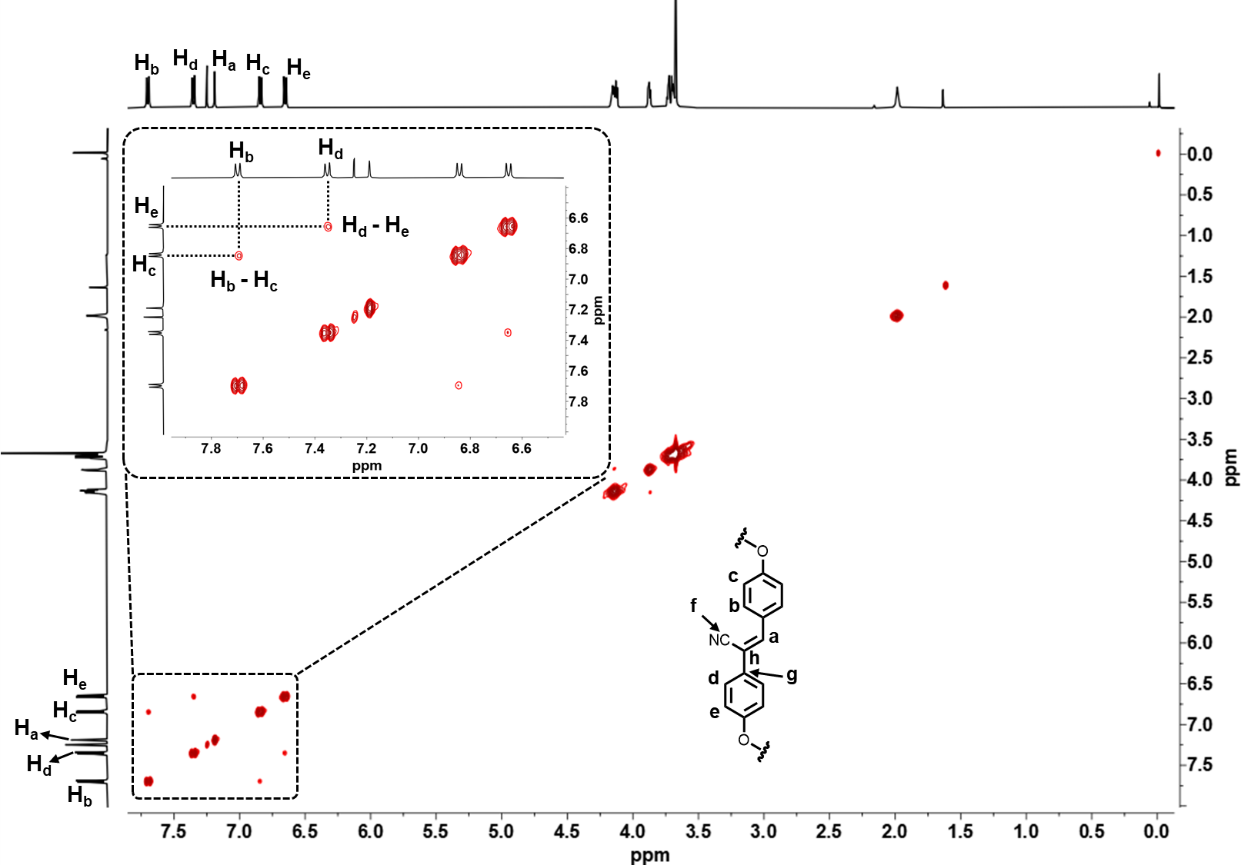
**

**Figure S18.** COSY spectrum of **MCH2**; 298 K, CHCl_3_-d.

**Photoisomerization of MCH2 to cMCH2**

**
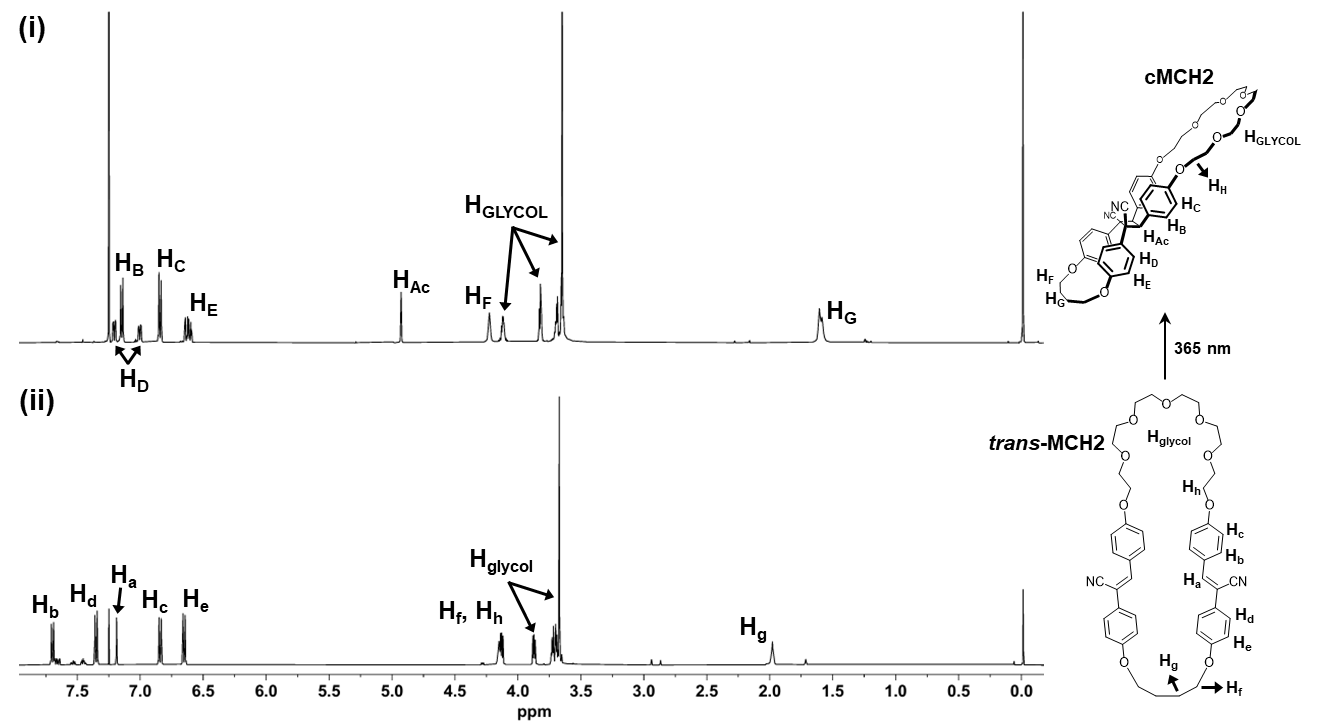
**

**Figure S19.** ^1^H NMR spectra of **MCH2** (i) and **MCH2** after irradiation by 365 nm UV light at -78 °C overnight (ii); 298 K, CHCl_3_-d.

**Structural analysis cMCH2 by ^1^H NMR, HSQC, HMBC and COSY**

**
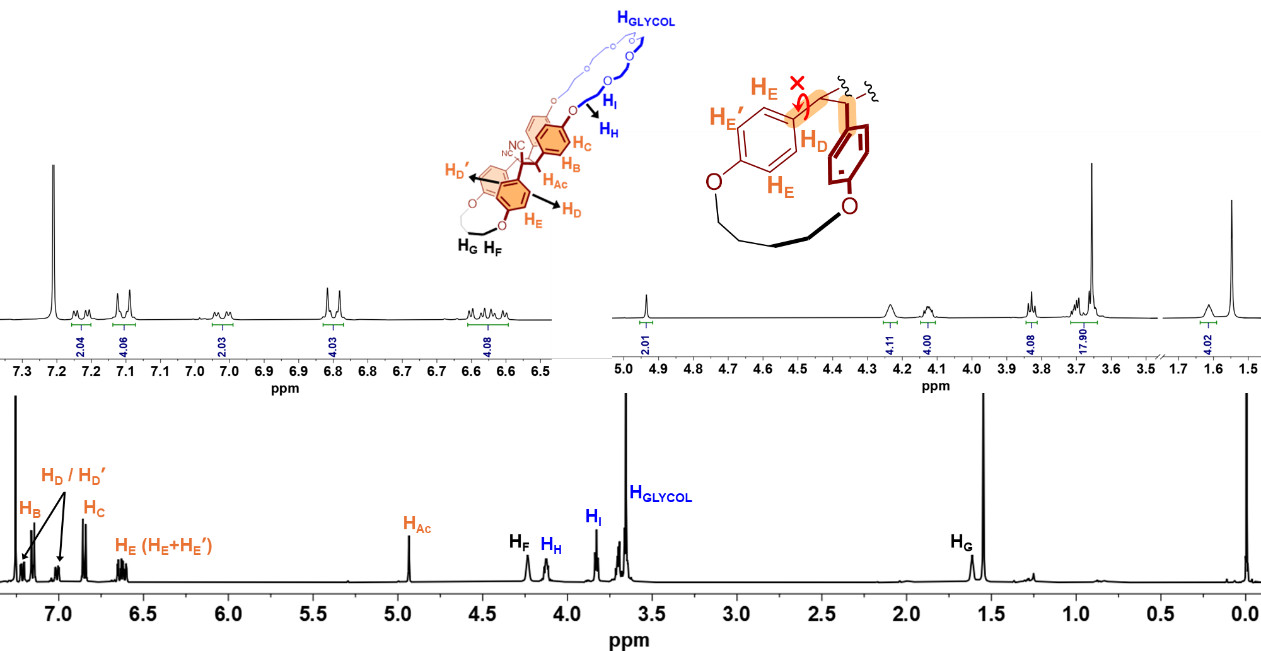
**

**Figure S20.** ^1^H NMR spectrum of **cMCH2**; 298 K, CHCl_3_-d.

**
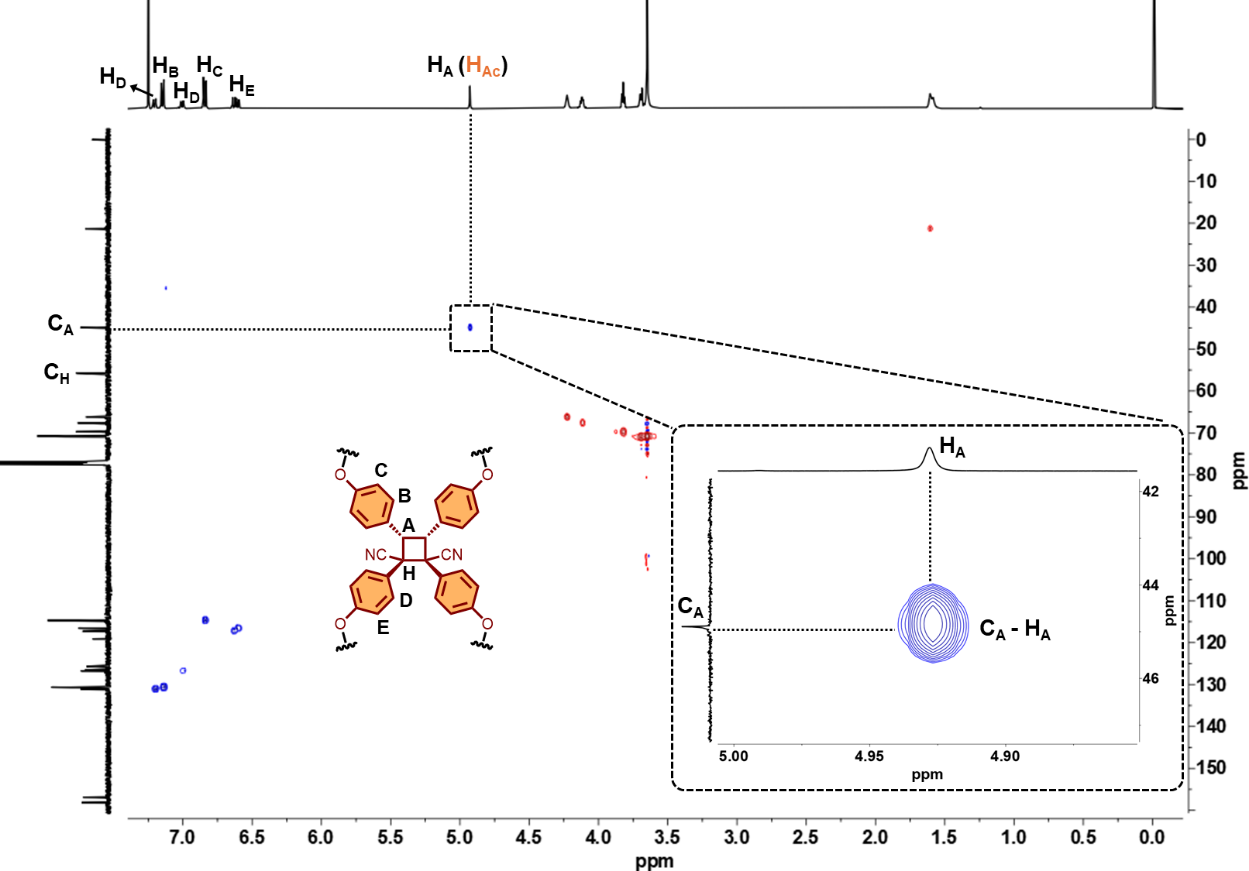
**

**Figure S21.** HSQC spectrum of **cMCH2**; 298 K, CHCl_3_-d.

**
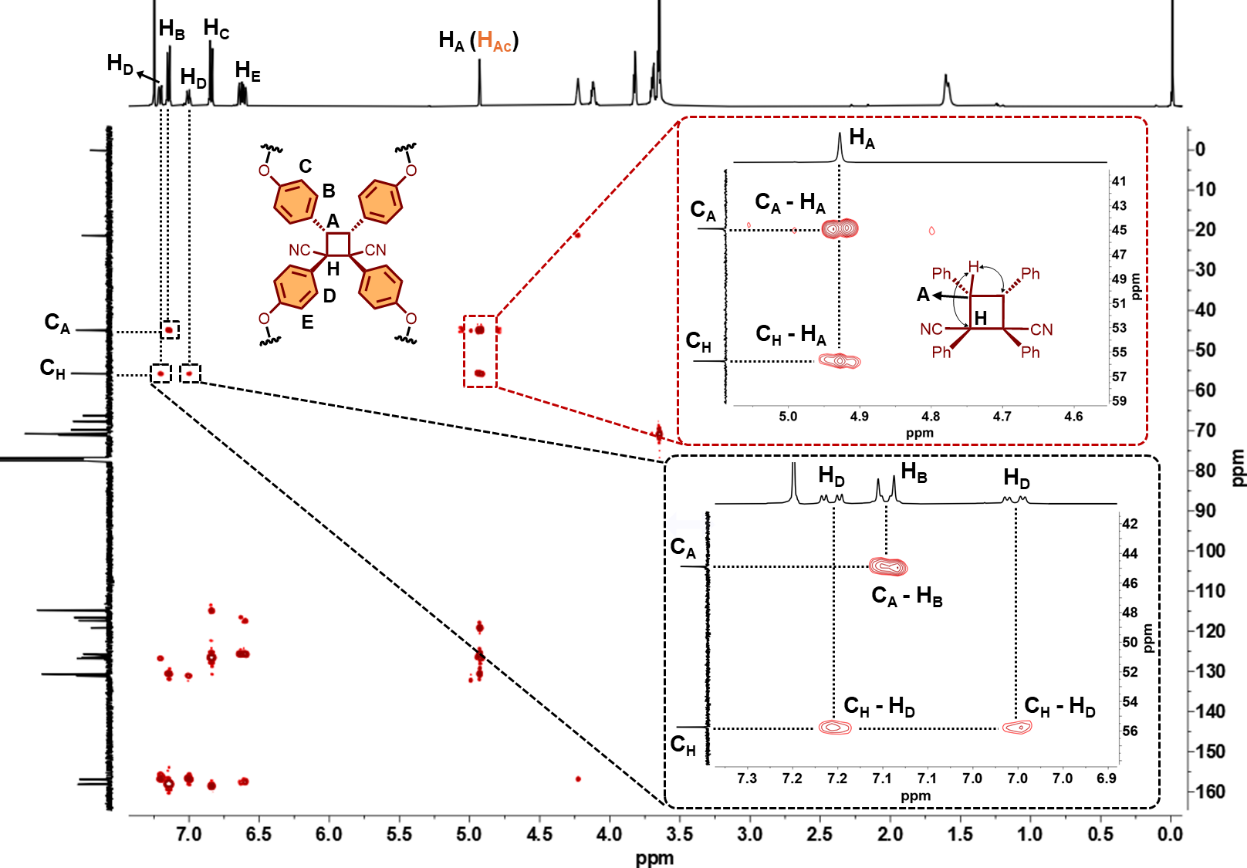
**

**Figure S22.** HMBC spectrum of **cMCH2**; 298 K, CHCl_3_-d.

**
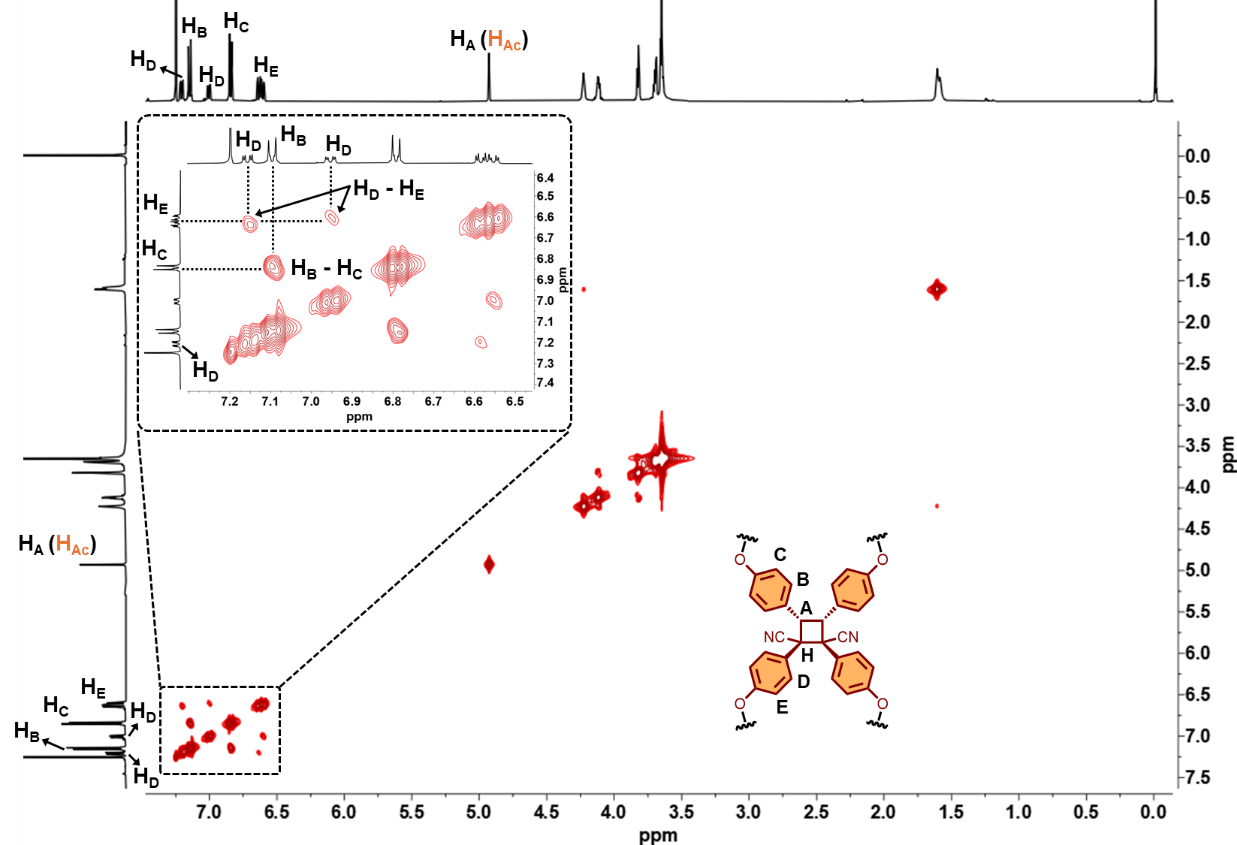
**

**Figure S23.** COSY spectrum of **cMCH2**; 298 K, CHCl_3_-d.

**2.3 MCH3**

**Structural analysis MCH3 by ^1^H NMR, HSQC, HMBC and COSY**

**
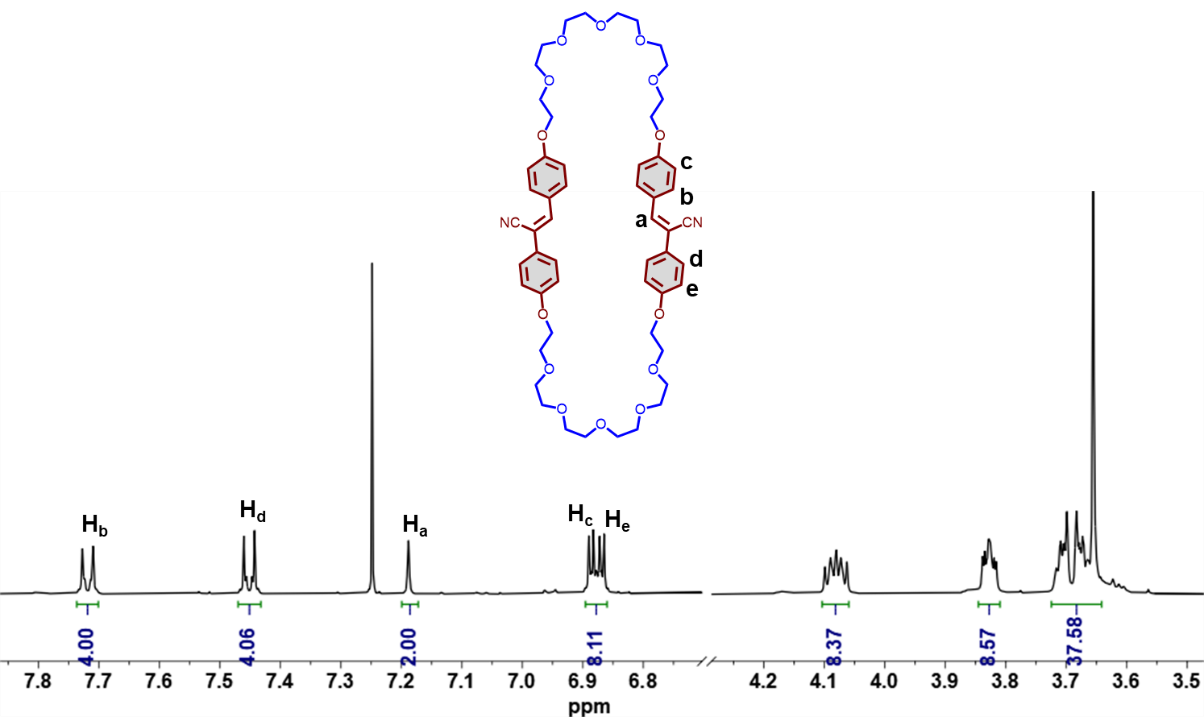
**

**Figure S24.** ^1^H NMR spectrum of **MCH3**; 298 K, CHCl_3_-d.

**
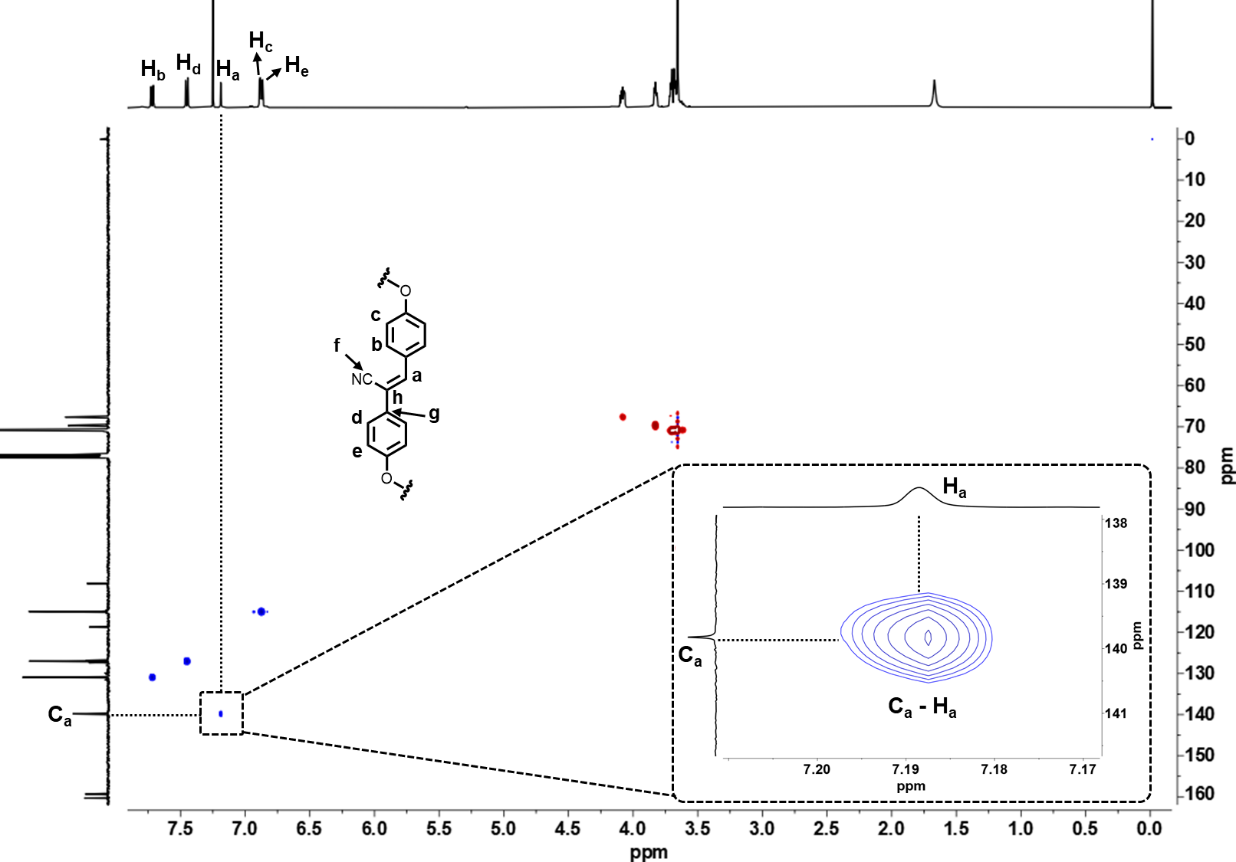
**

**Figure S25.** HSQC spectrum of **MCH3**; 298 K, CHCl_3_-d.

**
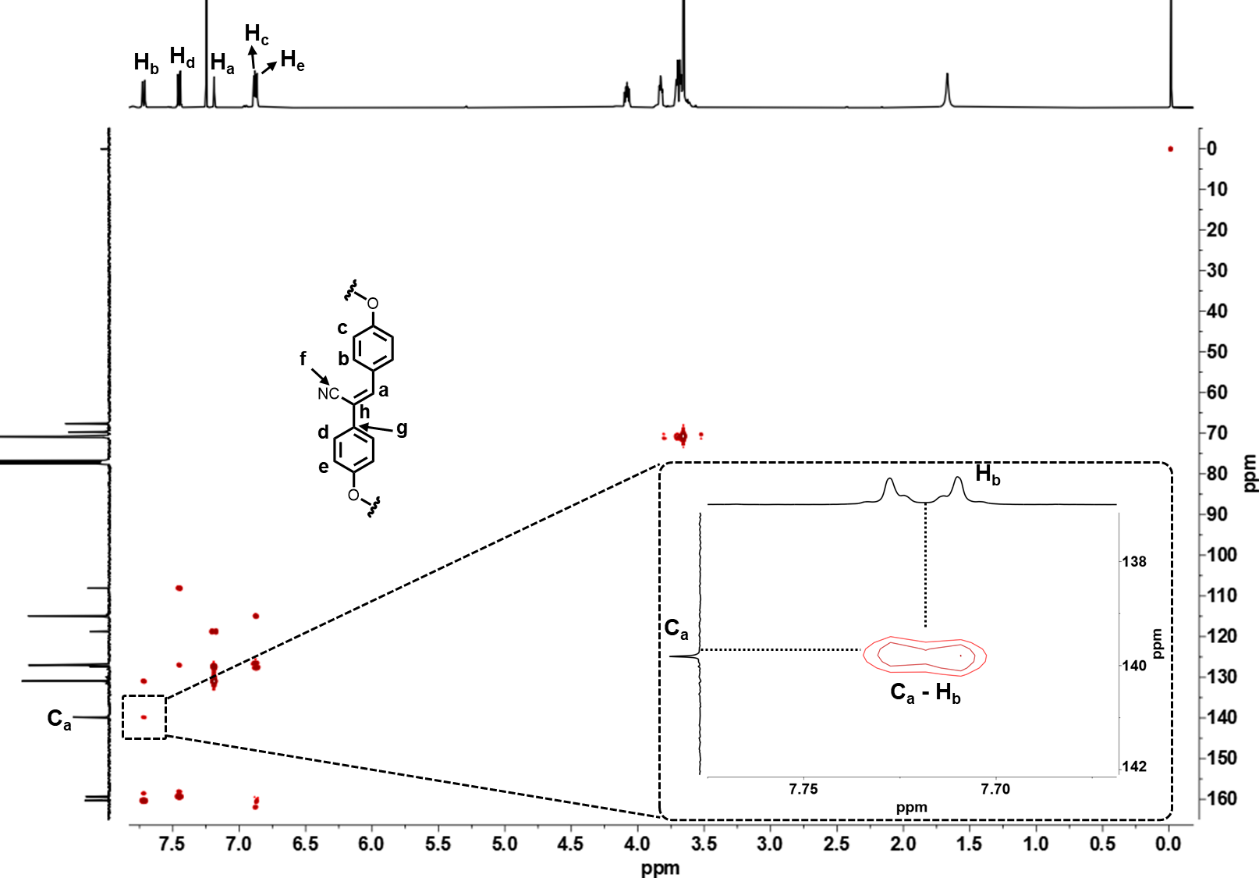
**

**Figure S26.** HMBC spectrum of **MCH3**; 298 K, CHCl_3_-d.

**
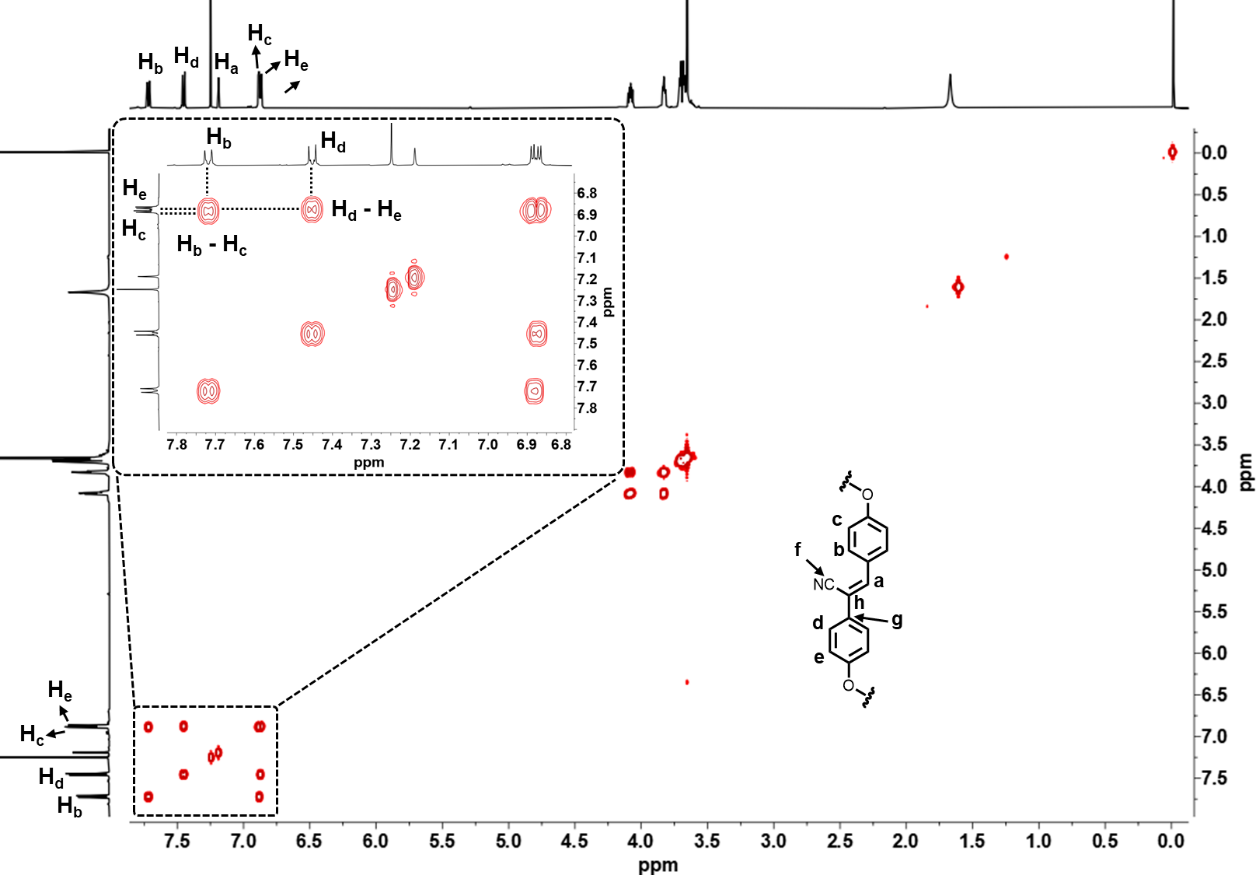
**

**Figure S27.** COSY spectrum of **MCH3**; 298 K, CHCl_3_-d.

**Photoisomerization of MCH3 to cMCH3** **and oMCH3**

**
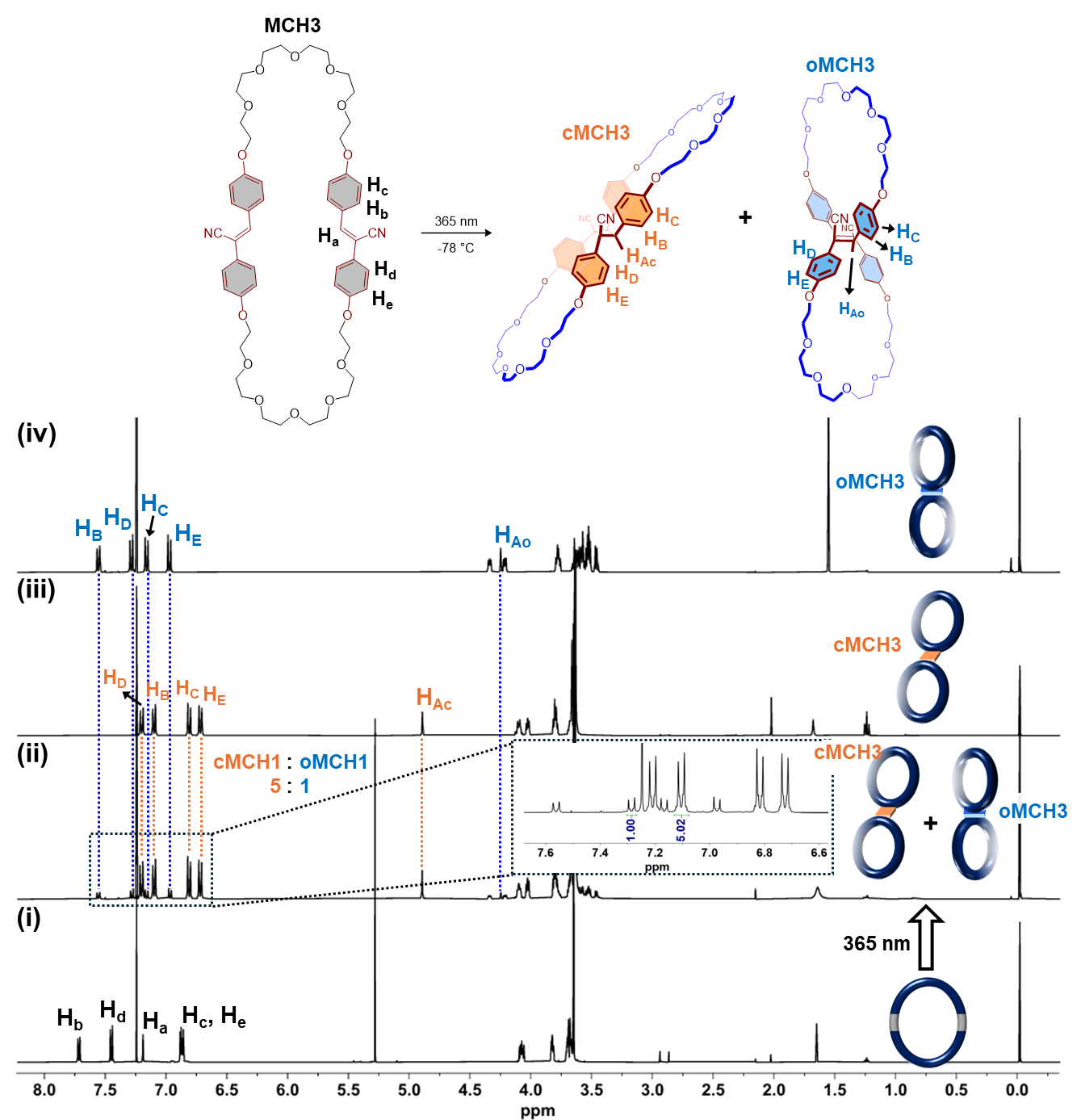
**

**Figure S28.** ^1^H NMR spectra of **MCH3** (i), **MCH3** after irradiation by 365 nm UV light at -78 °C overnight (ii), isolated **cMCH3** (iii) and isolated **oMCH3** (iv); 298 K, CHCl_3_-d.

**Structural analysis cMCH3 by ^1^H NMR, HSQC, HMBC and COSY**

**
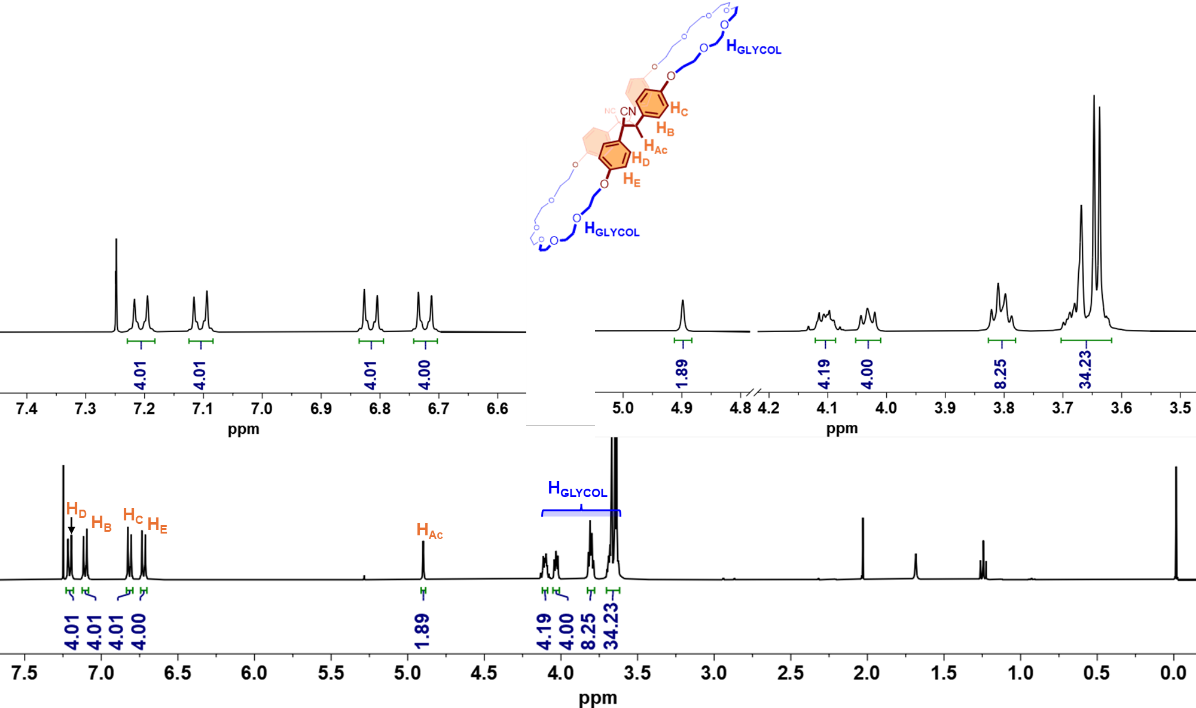
**

**Figure S29.** ^1^H NMR spectrum of **cMCH3**; 298 K, CHCl_3_-d.

**
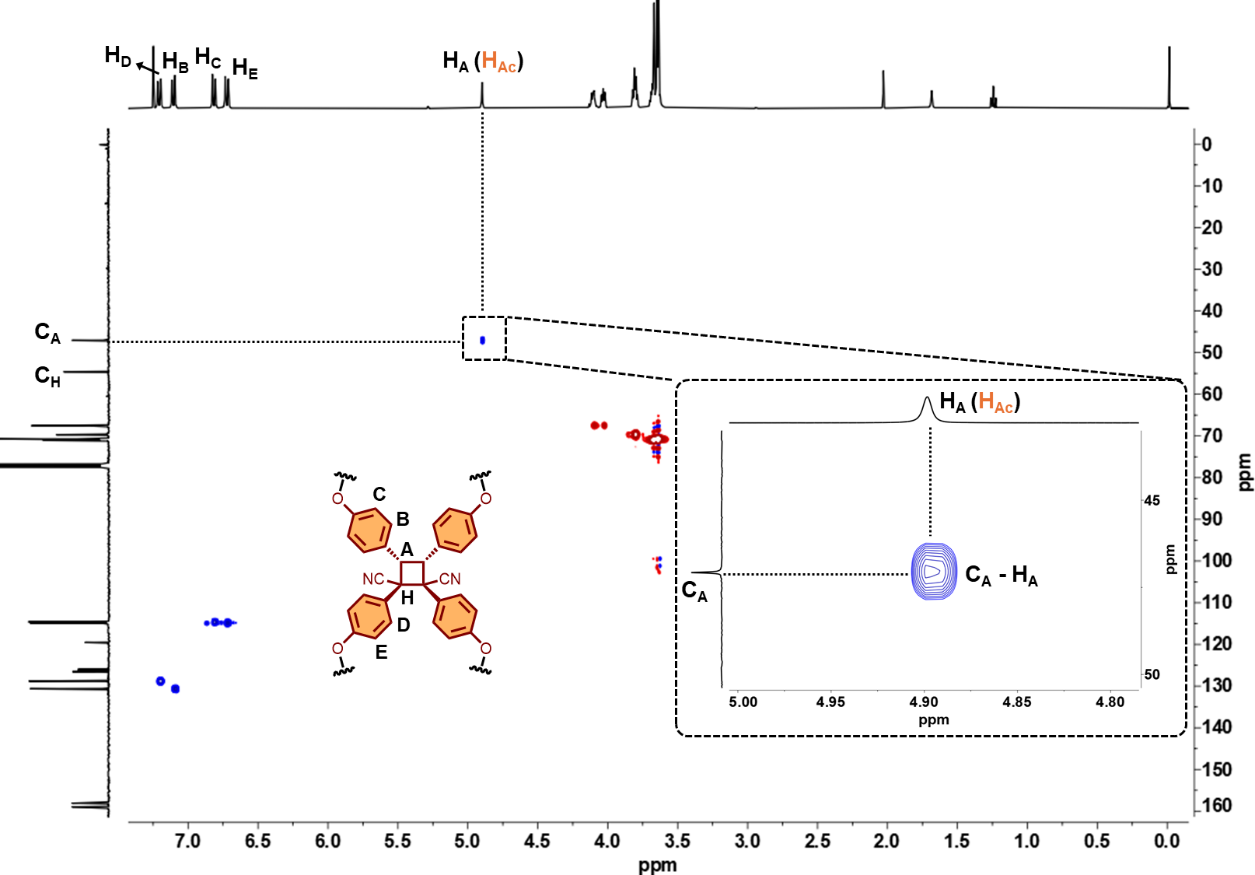
**

**Figure S30.** HSQC spectrum of **cMCH3**; 298 K, CHCl_3_-d.

**
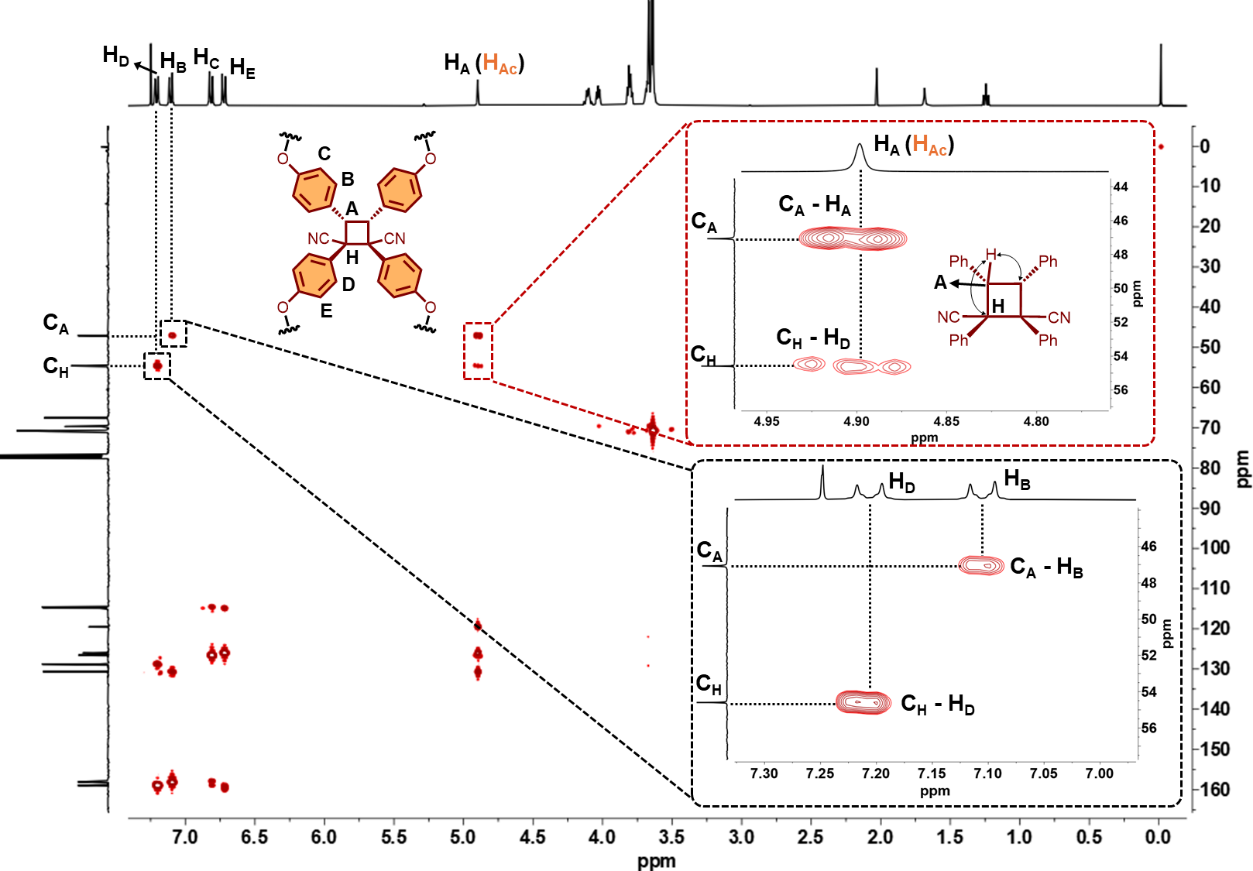
**

**Figure S31.** HMBC spectrum of **cMCH3**; 298 K, CHCl_3_-d.

**
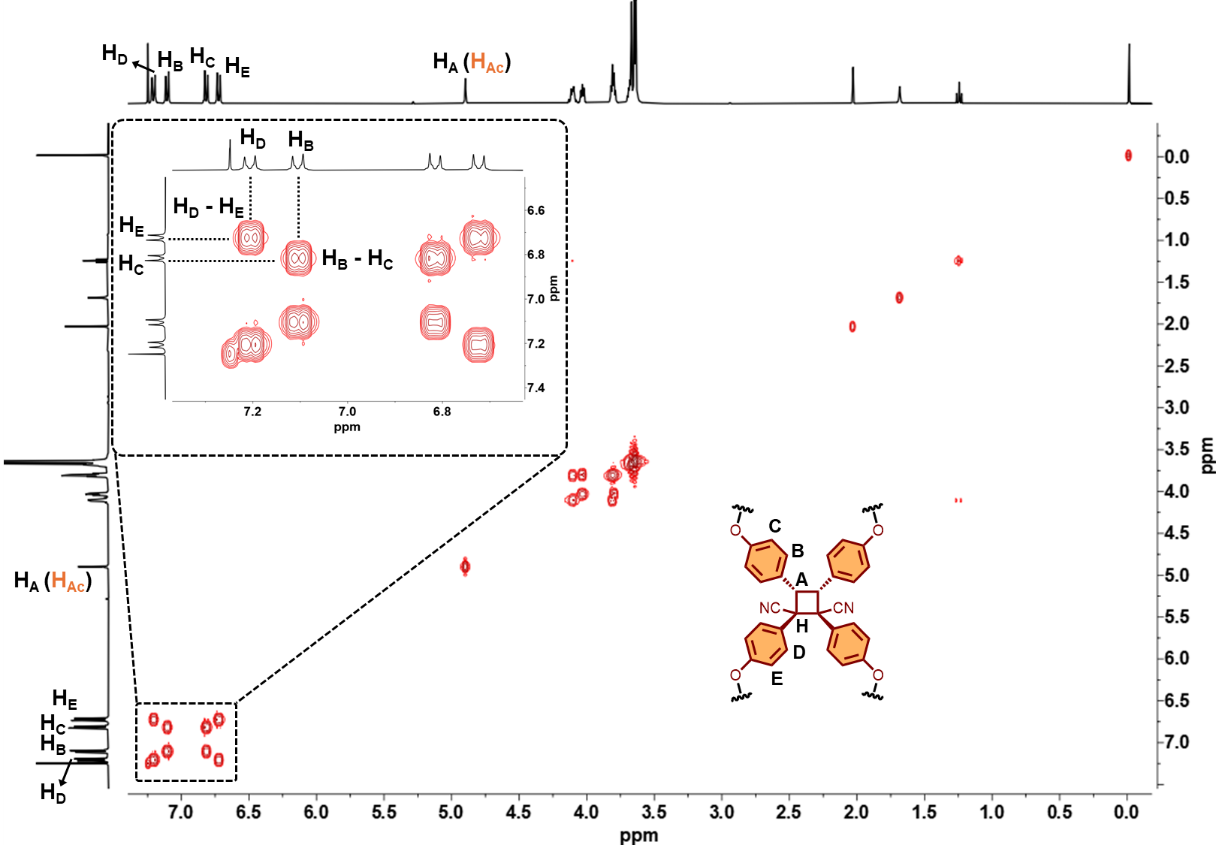
**

**Figure S32.** COSY spectrum of **cMCH3**; 298 K, CHCl_3_-d.

**Association of cMCH3 and dibenzylammonium (DBA^+^)**

The complexation of **cMCH3** with dibenzylammonium **(DBA^+^)** was investigated by NMR titration experiments. K_a1_ and K_a2_ are determined as 248 M^-1^ and 256 M^-1^, respectively. Cooperativity factor (α) is calculated as 4.13, indicating a positive cooperativity effect.

**
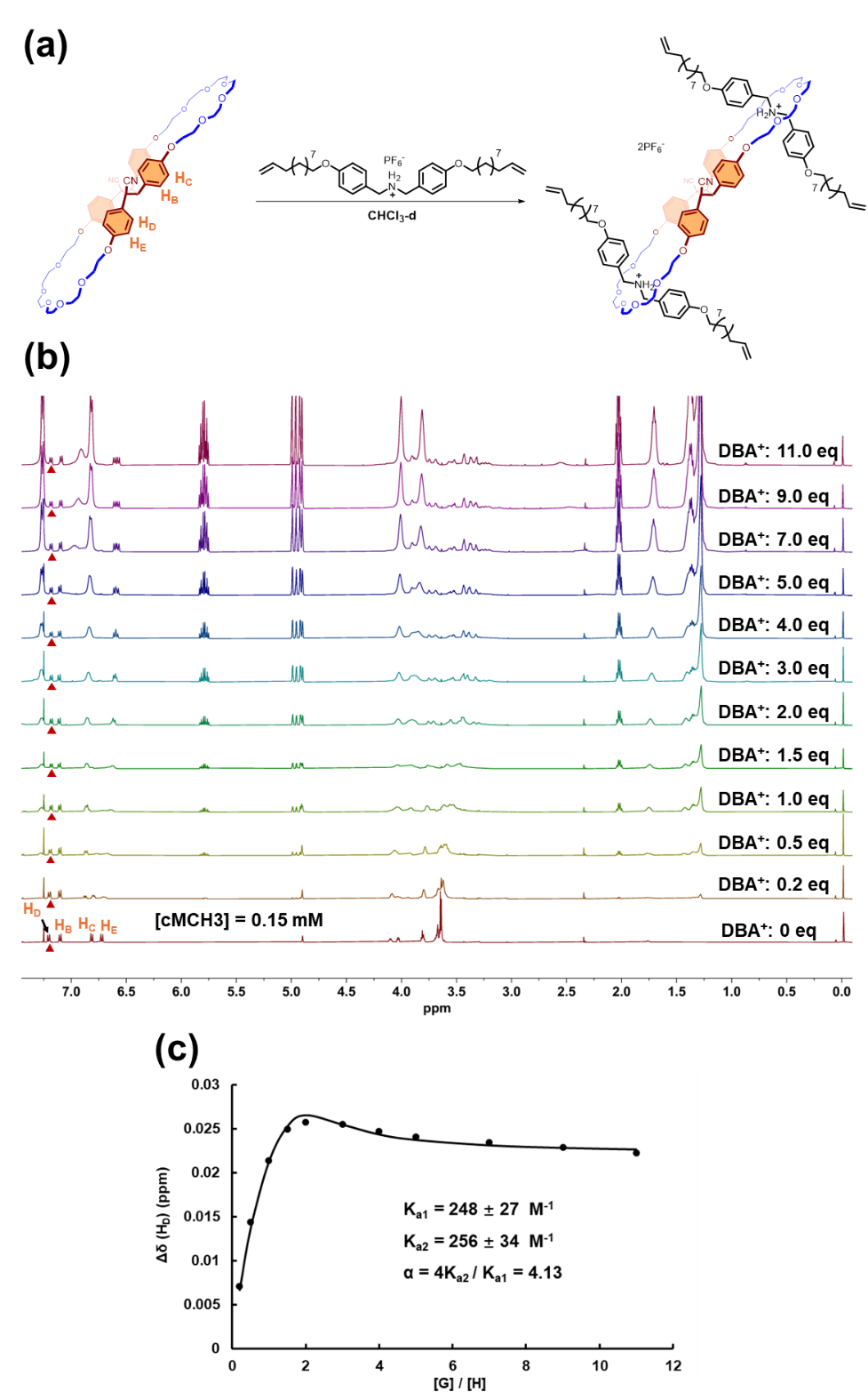
**

**Figure S33.** (a) Association of **cMCH3** and **DBA^+^**. (b) ^1^H NMR spectra of mixture of **cMCH3** and **DBA^+^** with increasing equivalence of **DBA^+^**. (c) Association constant (K_a_) between **cMCH1** and **DBA^+^**.

**Structural analysis oMCH3 by ^1^H NMR, HSQC, HMBC and COSY**

**Figure S34.** ^1^H NMR spectrum of **oMCH3**; 298 K, CHCl_3_-d.

**Figure S35.** HSQC spectrum of **oMCH3**; 298 K, CHCl_3_-d.

**Figure S36.** HMBC spectrum of **oMCH3**; 298 K, CHCl_3_-d.

**Figure S37.** COSY spectrum of **oMCH3**; 298 K, CHCl_3_-d.

**3.** **Reverse topology transformation of cMCH1, cMCH2, and cMCH3 from figure-eight structures to macrocycles**

The compound for NMR analysis was dissolved in 0.6 mL of chloroform-d, and the solution was transferred into an NMR tube, which was then sealed. The sealed NMR tube was immersed in an oil bath maintained at 80 °C or 90 °C, ensuring that the solution was completely submerged. After heating for a predetermined period, the NMR tube was removed from the oil bath and immediately subjected to NMR measurement without further treatment.

To evaluate the reaction kinetics of mechanical unlocking via reverse topology transformation, we monitored the conversion of each system over time under constant heating conditions. The time–conversion data were used to test for first-order kinetics by plotting ln(1−x) versus time, where x is the fractional conversion at each time point.

A linear relationship between ln (1−x) and time was observed for all systems, indicating that the reactions follow pseudo-first-order kinetics under applied conditions. The rate constant k for each system was determined from the slope of the linear regression:

ln(1−x) =kt

The calculated rate constants and correlation coefficients (R²) are shown within each plot.

**3.1 cMCH1**

**Figure S38.** ^1^H NMR spectra of sealed CHCl₃-d solution of **cMCH1** after heating in oil bath at 80 °C for 150 minutes, followed by heating in oil bath at 90 °C for 30 minutes in the solid state; 298 K, CHCl_3_-d.

**Figure S39.** ^1^H NMR spectra of **oMCH1** (i) and sealed CHCl_3_ solution of **oMCH1** after heating in oil bath at 80 °C for 150 minutes (ii); 298 K, CHCl_3_-d.

**3.2 cMCH2**

**Figure S40.** ^1^H NMR spectra of sealed CHCl_3_-d solution of **cMCH2** after heating in oil bath at 80 °C for 150 minutes, followed by heating at 90 °C for 60 minutes in the solid state; 298 K, CHCl_3_-d.

**3.3 cMCH3**

**Figure S41.** ^1^H NMR spectra of sealed CHCl_3_-d solution of **cMCH3** after heating in oil bath at 80 °C for 150 minutes, followed by heating at 90 °C for 30 minutes in the solid state; 298 K, CHCl_3_-d.

**Figure S42.** ^1^H NMR spectra of **oMCH3** (i) and sealed CHCl_3_ solution of **oMCH3** after heating at oil bath at 80 °C for 150 minutes (ii); 298 K, CHCl_3_-d.

**3.4 Comparison of topology transformation rate among cMCH1-3**

**Figure S43.** Conversion-Heating time curve of **cMCH1-3** (a) and k of topology transformation of **cMCH1-3** (b). The asterisk “*****” denotes the complete conversion achieved after additional heating: 30 minutes at 90 °C for **cMCH1** and **cMCH3**, and 60 minutes for **cMCH2**.

**4. Synthesis of chair-like and orthogonal rotaxanes via topology transformation**

**4.1 Synthesis of c[2]RT(cMCH1@G) and o[2]RT(oMCH1@G) by using MCH1 and G**

**Complexation between MCH1 and G**

**Figure S44.** ^1^H NMR spectra of **G** (i), **MCH1** (ii), and mixture of **G** and **MCH1** in a ratio of 3:1 ([**MCH1**] = 15 mM) (iii); 298 K, acetone-d6**.**

**Irradiation of mixture solution of MCH1 and G**

**Figure S45.** ^1^H NMR spectra of **G** (i), crude after irradiating the mixture of **G** (3 eq) and **MCH1** (1 eq, 5 mM) by 365 nm UV light overnight at -78 °C (ii), isolated **c[2]RT(cMCH1@G)** (iii), isolated **o[2]RT(oMCH1@G)** (iv) and **cMCH1** (vi); 298 K, acetone-d6. The asterisk “*****” in spectrum (ii) marks an impurity generated from **G** after it was left standing in acetone solution (See Figure S43).

**Figure S46.** ^1^H NMR spectra of **G** (i) and **G** after being left standing in acetone at -78 °C overnight (ii); 298 K, acetone-d6**.** The synthetic **G** occasionally generates an impurity when dissolved in acetone and left to stand overnight.

**Structural analysis c[2]RT(cMCH1@G) by ^1^H NMR, HSQC, HMBC, COSY and ROESY**

**Figure S47.** ^1^H NMR spectrum of **c[2]RT(cMCH1@G)**; 298 K, acetone-d6**.**

**Figure S48.** HSQC spectrum of **c[2]RT(cMCH1@G)**; 298 K, acetone-d6**.**

**Figure S49.** HMBC spectrum of **c[2]RT(cMCH1@G)**; 298 K, acetone-d6**.**

**Figure S50.** COSY spectrum of **c[2]RT(cMCH1@G)**; 298 K, acetone-d6**.**

**Figure S51.** ROESY spectrum of **c[2]RT(cMCH1@G)**; 298 K, acetone-d6**.**

Patial ROESY spectra of **c[2]RT(cMCH1@G)**; 298 K, acetone-d6**.**

**Structural analysis o[2]RT(oMCH1@G) by ^1^H NMR, HSQC, HMBC, COSY and ROESY**

**Figure S52.** ^1^H NMR spectrum of **o[2]RT(oMCH1@G)**; 298 K, acetone-d6**.**

**Figure S53.** HSQC spectrum of **o[2]RT(oMCH1@G)**; 298 K, acetone-d6**.**

**Figure S54.** HMBC spectrum of **o[2]RT(oMCH1@G)**; 298 K, acetone-d6**.**

**Figure S55.** COSY spectrum of **o[2]RT(oMCH1@G)**; 298 K, acetone-d6**.**

**Figure S56.** ROESY spectrum of **o[2]RT(oMCH1@G)**; 298 K, acetone-d6**.**

Patial ROESY spectrum of **o[2]RT(oMCH1@G)**; 298 K, acetone-d6**.**

**4.2 Synthesis of c[2]RT(cMCH2@G) by using MCH2 and G**

**Figure S57.** ^1^H NMR spectra of **G** (i), **MCH2** (ii), and mixture of **G** and **MCH2** in a ratio of 3:1 ([**MCH2**] = 15 mM) (iii); 298 K, acetone-d6**.**

**Figure S58.** ^1^H NMR spectra of **G** (i), crude after irradiating the mixture of **G** (3 eq) and **MCH2** (1 eq, 5 mM) by 365 nm UV light overnight at -78 °C (ii), isolated **c[2]RT(cMCH2@G)** (iii), and **cMCH2** (iv); 298 K, acetone-d6**.**

**Structural analysis c[2]RT(cMCH2@G) by ^1^H NMR, HSQC, HMBC, COSY and ROESY**

**Figure S59.** ^1^H NMR spectrum of **c[2]RT(cMCH2@G)**; 298 K, acetone-d6**.**

**Figure S60.** HSQC spectrum of **c[2]RT(cMCH2@G)**; 298 K, acetone-d6**.**

**Figure S61.** HMBC spectrum of **c[2]RT(cMCH2@G)**; 298 K, acetone-d6**.**

**Figure S62.** COSY spectrum of **c[2]RT(cMCH2@G)**; 298 K, acetone-d6**.**

**Figure S63.** ROESY spectrum of **c[2]RT(cMCH2@G)**; 298 K, acetone-d6**.**

Patial ROESY spectra of **c[2]RT(cMCH2@G)**; 298 K, acetone-d6**.**

**4.3 Synthesis of bis-c[2]RT(cMCH3@G) and bis-oRT(oMCH3@G) by using MCH3 and G**

**Figure S64.** ^1^H NMR spectra of **G** (i), **MCH3** (ii), and mixture of **G** and **MCH3** in a ratio of 4:1 ([**MCH2**] = 15 mM) (iii); 298 K, acetone-d6**.**

**Figure S65.** ^1^H NMR spectra of **G** (i), crude after irradiating the mixture of **G** (4 eq) and **MCH3** (1 eq, 5 mM) by 365 nm UV light overnight at -78 °C (ii), isolated **bis-c[2]RT(cMCH3@G)** (iii), isolated **bis-oRT(oMCH3@G)** (iv) and **cMCH3** (vi); 298 K, acetone-d6**.** The asterisk “*****” in spectrum (ii) marks an impurity generated from **G** after it was left standing in acetone solution (See Figure S43).

**Structural analysis bis-c[2]RT(cMCH3@G) by ^1^H NMR, HSQC, HMBC, COSY and ROESY**

**Figure S66.** ^1^H NMR spectrum of **bis-c[2]RT(cMCH3@G)**; 298 K, acetone-d6**.**

**Figure S67.** HSQC spectrum of **bis-c[2]RT(cMCH3@G)**; 298 K, acetone-d6**.**

**Figure S68.** HMBC spectrum of **bis-c[2]RT(cMCH3@G)**; 298 K, acetone-d6**.**

**Figure S69.** COSY spectrum of **bis-c[2]RT(cMCH3@G)**; 298 K, acetone-d6**.**

**Figure S70.** ROESY spectrum of **bis-c[2]RT(cMCH3@G)**; 298 K, acetone-d6**.**

Patial ROESY spectra of **bis-c[2]RT(cMCH3@G)**; 298 K, acetone-d6**.**

**Structural analysis bis-oRT(oMCH3@G) by ^1^H NMR, COSY and ROESY**

**Figure S71.** ^1^H NMR spectrum of **bis-oRT(oMCH3@G)**; 298 K, acetone-d6**.**

**Figure S72.** COSY spectrum of **bis-oRT(oMCH3@G)**; 298 K, acetone-d6**.**

**Figure S73.** ROESY spectrum of **bis-oRT(oMCH3@G)**; 298 K, acetone-d6**.**

Patial ROESY spectra of **o[3]RT(cMCH2@G)**; 298 K, acetone-d6**.**

**5. Transition from chair-like rotaxanes to *pseudo*-rotaxanes via reverse topology transformation**

The compound for NMR analysis was dissolved in 0.6 mL of chloroform-d, and the solution was transferred into an NMR tube, which was then sealed. The sealed NMR tube was immersed in an oil bath maintained at 80 °C or 90 °C, ensuring that the solution was completely submerged. After heating for a predetermined period, the NMR tube was removed from the oil bath and immediately subjected to NMR measurement without further treatment.

**c[2]RT(cMCH1@G)** and **c[2]RT(cMCH2@G)** show clean conversion to their pseudo-rotaxane form. The typical split glycol protons due to complexation with DBA^+^ were observed by ^1^H NMR spectrum. The MALDI-TOF MS spectrum also shows the existence of free macrocycles, free **G** and macrocycle-**G** complex.

Heating **bis[2]RT(cMCH3@G)** proceed characteristic signals for **MCH3**, while no split glycol protons were observed, although the MALDI-TOF MS spectrum show the existence of macrocycle-**G** complex.

**5.1 Transition from c[2]RT(cMCH1@G) to *pseudo*-rotaxane**

**Figure S74.** ^I^H NMR spectra of sealed CHCl_3_ solution of **c[2]RT(cMCH1@G)** after heating in oil bath at 80 °C for 150 minutes; 298 K, CHCl_3_-d.

**Figure S75.** ^I^H NMR spectra of DMSO solution of **c[2]RT(cMCH1@G)** after heating at 40 °C for 1 day; 298 K, DMSO-d_6_.

**5.2 Transition from c[2]RT(cMCH2@G) to *pseudo*-rotaxane**

**Figure S76.** ^1^H NMR spectra of sealed CHCl_3_ solution of **c[2]RT(cMCH2@G)** after heating at 80 °C for 150 minutes, followed by heating in 90 °C bath for additional 30 minutes; 298 K, CHCl_3_-d.

**5.3 Transition from bis-c[2]RT(cMCH3@G) to *pseudo*-rotaxane**

**Figure S77.** ^1^H NMR spectra of sealed CHCl_3_ solution of **bis-c[2]RT(cMCH3@G)** after heating at 80 °C for 150 minutes, followed by heating at 90 °C bath for additional 60 minutes; 298 K, CHCl_3_-d.

**5.4 Comparison of topology transformation rate of all chair-like compounds**

**Figure S78.** k of all chair-like compounds

**5.5 Thermal stability of orthogonal rotaxanes**

**Figure S79.** ^1^H NMR spectra of **o[2]RT(oMCH1@G)** (i) and sealed CHCl_3_ solution of **o[2]RT(oMCH1@G)** after heating in oil bath at 80 °C for 150 minutes; 298 K, CHCl_3_-d.

**Figure S80.** ^1^H NMR spectra of **bis-oRT(oMCH3@G)** (i) and sealed CHCl_3_ solution of **bis-oRT(oMCH3@G)** after heating in oil bath at 80 °C for 150 minutes; 298 K, CHCl_3_-d.

**6. References**

1. Y. Liu, X. Li, Q. Liu, X. Li, H. Liu, *Org. Lett.*, **2022**, *24*, 6604–6608.

2. P. Kokkala, A. Mpakali, F. X. Mauvais, A. Papakyriakou, I. Daskalaki, I. Petropoulou, D. Georgiadis, *J. Med. Chem.*, **2016**, *59*, 9107–9123.

3. W. G. Kong, H. J. An, Q. L. Song, *Chem. Commun.*, **2017**, *53*, 8968–8971.

4. M. S. Palmquist, M. C. Gruschka, J. M. Dorsainvil, A. O. Delawder, T. M. Saak, M. K. Danielson, J. C. Barnes, *Polym. Chem.*, **2022**, *13*, 2115–2122.

5. A. Balamurugan, M. L. P. Reddy, M. Jayakannan, *J. Mater. Chem. A*, **2013**, *1*, 2256–2266.

6. A. B. Jamdade, D. V. Sutar, B. Gnanaprakasam, *Org. Lett.*, **2023**, *25*, 9058–9063.

7. M. Shan, K. E. Carlson, A. Bujotzek, A. Wellner, R. Gust, M. Weber, R. Haag, *ACS Chem. Biol.*, **2013**, *8*, 707–715.

8. Crysalispro, 1.171.41.122, **2021**, Rigaku OD.

9. G. M. Sheldrick, *Acta Crystallogr. A*, **2015**, *71*, 3–8.

10. G. M. Sheldrick, *Acta Crystallogr. C*, **2015**, *71*, 3–8.

11. O. V. Dolomanov, L. J. Bourhis, R. J. Gildea, J. A. Howard, H. Puschmann, *J. Appl. Crystallogr.*, **2009**, *42*, 339–341.

12. J. W. Chung, Y. You, H. S. Huh, B. K. An, S. J. Yoon, S. H. Kim, S. W. Lee, S. Y. Park, *J. Am. Chem. Soc.*, **2009**,*131*, 8163-8172.

13. P. F. Wei, J.-X. Zhang, Z. Zhao, Y. Chen, X. He, M. Chen, J. Gong, H. H.-Y. Sung, I. D. Williams, J. W. Y. Lam, B. Z. Tang, *J. Am. Chem. Soc.*, **2018**,*140*, 1966-1975.

14. T. Dünnebacke, K. K. Kartha, J. M. Wahl, R. Q. Albuquerque, G. Fernández., *Chem. Sci.*, **2020**, 11, 10405-10413.
